# Supplementary material for: From comparative gene content and gene order to ancestral contigs, chromosomes and karyotypes
Source: Sci Rep. 2023 Apr 13;13:6095. doi: 10.1038/s41598-023-33029-x (PMC10102168; doi:10.1038/s41598-023-33029-x)

Supplementary material for:

From comparative gene content and gene order to ancestral contigs, chromosomes and karyotypes

Qiaoji Xu[1], Lingling Jin[2], Chunfang Zheng[1], Xiaomeng Zhang[1], James Leebens-Mack[3] and David Sankoff[1*]

[1*]Department of Mathematics and Statistics, University of Ottawa, Ottawa, Ontario K1N 6N5, Canada.
[2]Department of Computer Science, University of Saskatchewan, Saskatoon, Saskatchewan S7N 5C9, Canada.
[3]Department of Plant Biology, University of Georgia, Athens, Georgia 30602, USA.

*Corresponding author(s). E-mail(s): sankoff@uottawa.ca;
Contributing authors: qxu062@uottawa.ca;
lingling.jin@cs.usask.ca; chunfang313@gmail.com;
xzhan408@uottawa.ca; jleebensmack@uga.edu;

A. Heat maps, Eleven orders, all ancestors $g = 20$

B. Evaluation graphs. Eleven orders

C. Gap statistics. For $g = 20$ ***each ancestor, eleven orders***

D. Painted extant genomes $g = 20$

E. PCA clustering $g = 20$

Supplement A. Heat maps, Eleven orders, all ancestors $g = 20$

Heat maps also available for 15-mers, 30-mers and 40-mers.

Ancestor 1 with 40-Mers

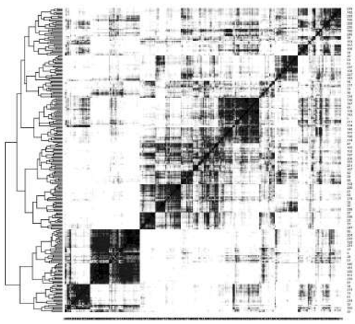

Ancestor 2 with 40-Mers

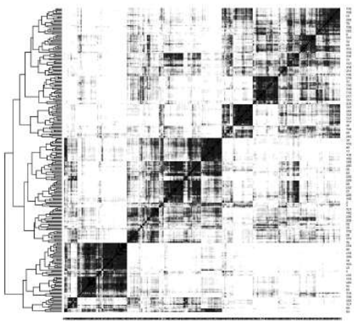

Ancestor 3 with 40-Mers

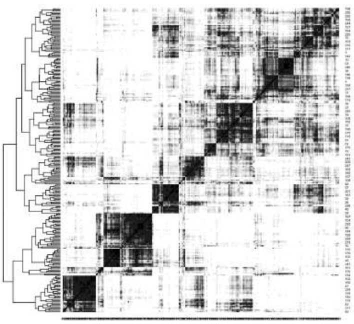

Ancestor 4 with 40-Mers

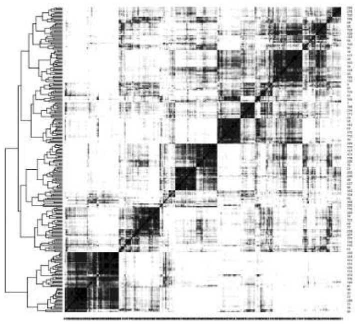

Ancestor 5 with 40-Mers

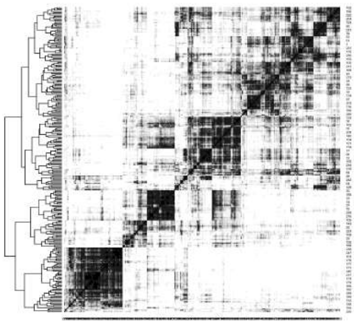

Ancestor 6 with 40-Mers

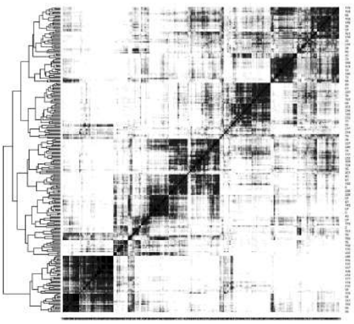

Ancestor 7 with 40-Mers

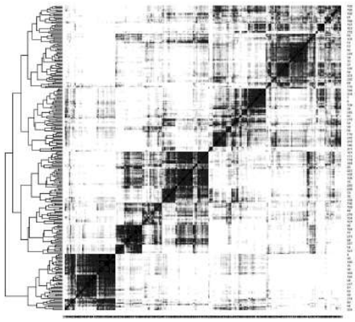

Fagales

Ancestor 1 with 20-Mers

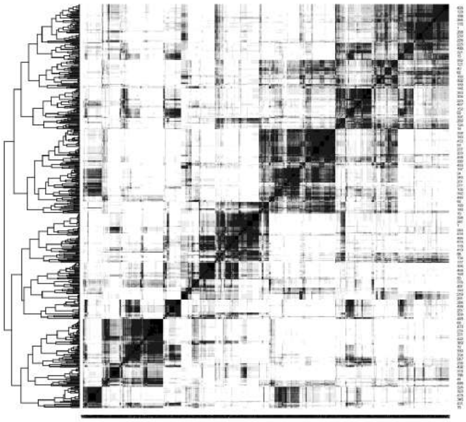

Ancestor 2 with 20-Mers

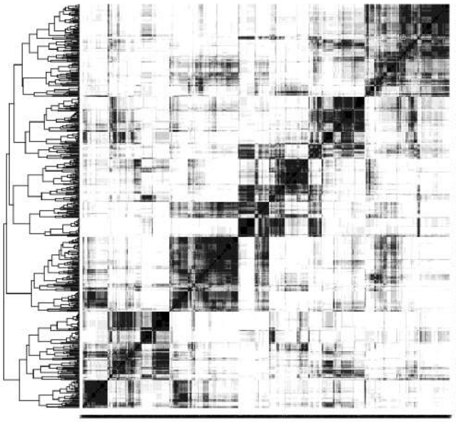

Ancestor 3 with 20-Mers

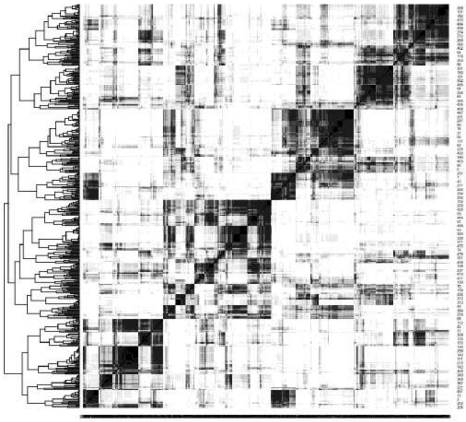

Ancestor 4 with 20-Mers

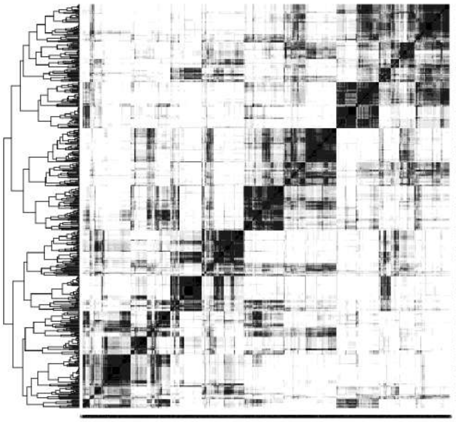

Ancestor 5 with 20-Mers

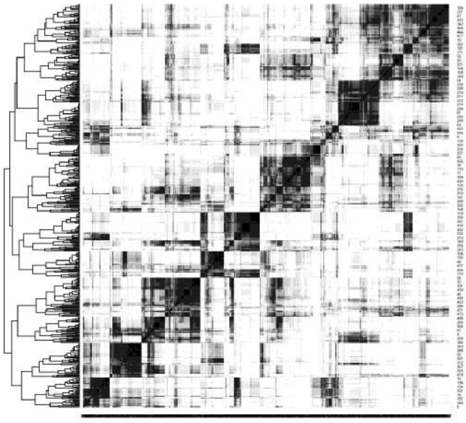

Cucurb

Ancestor 1 with 30-Mers

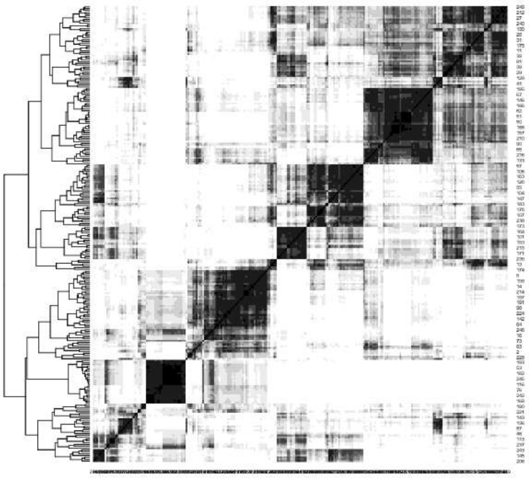

Ancestor 2 with 30-Mers

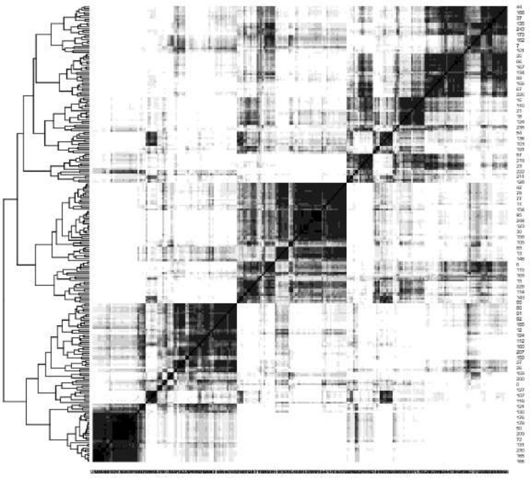

Ancestor 3 with 30-Mers

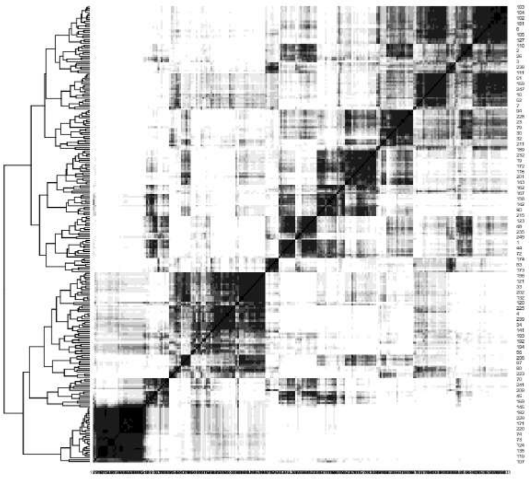

Ancestor 4 with 30-Mers

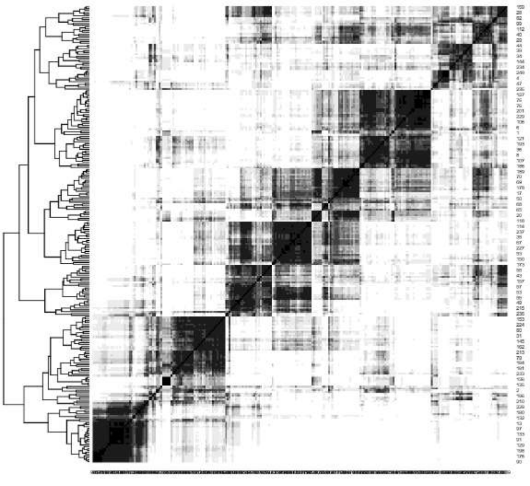

Malpighiales

Ancestor 1 with 20-Mers

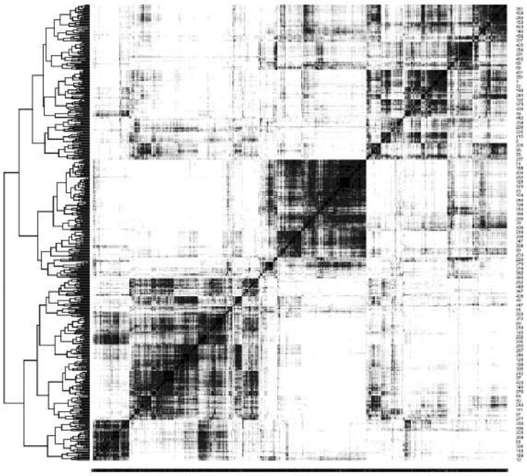

Ancestor 2 with 20-Mers

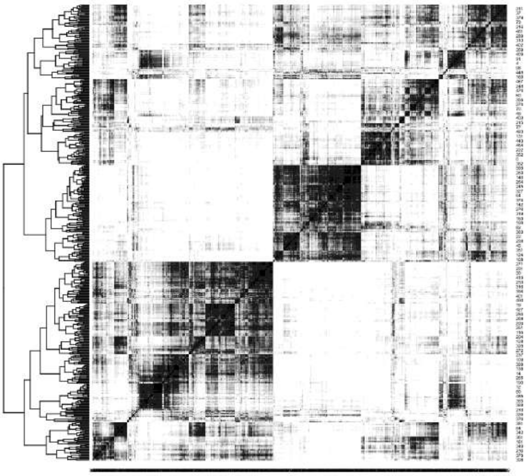

Ancestor 3 with 20-Mers

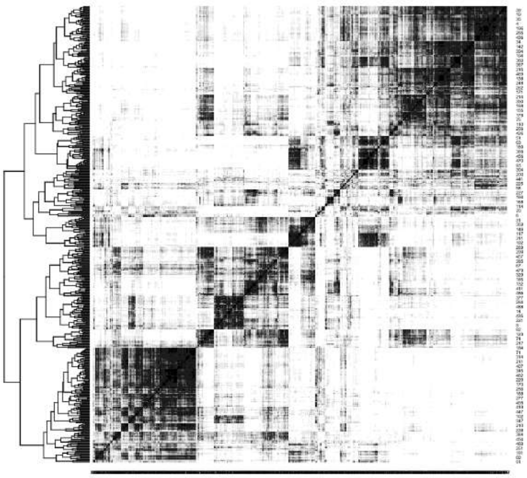

Myrtales

Ancestor 1 with 30-Mers

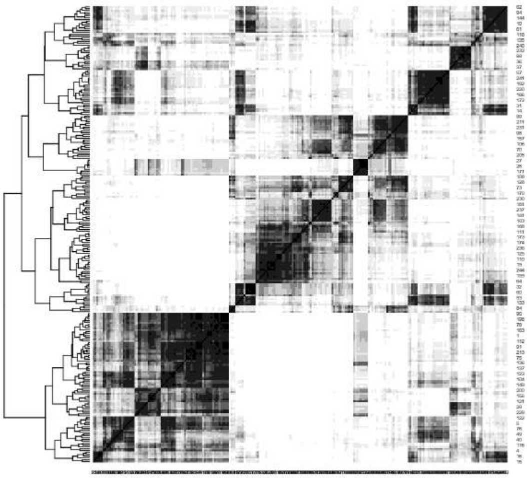

Ancestor 2 with 30-Mers

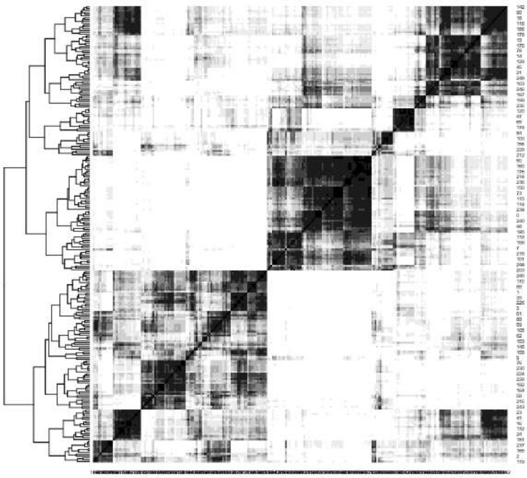

Ancestor 3 with 30-Mers

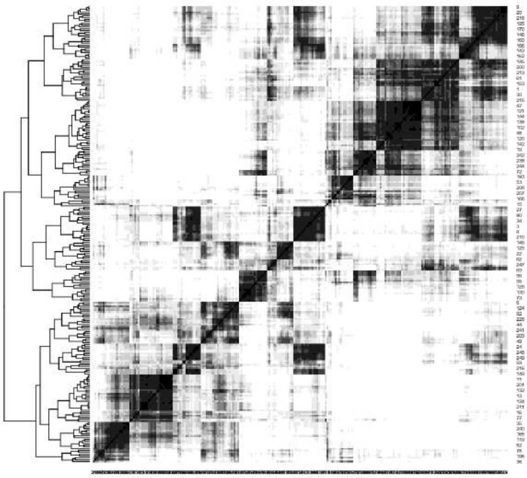

Ancestor 4 with 30-Mers

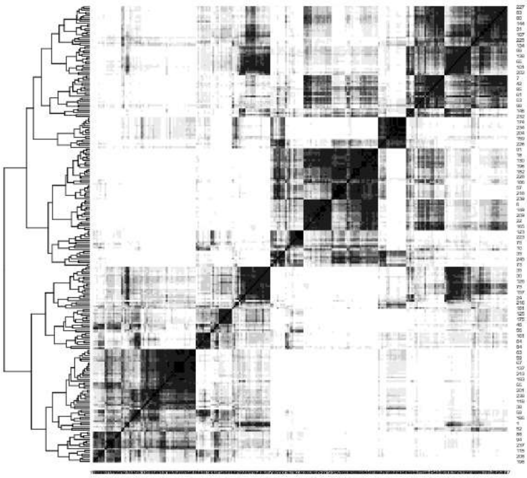

Malvales

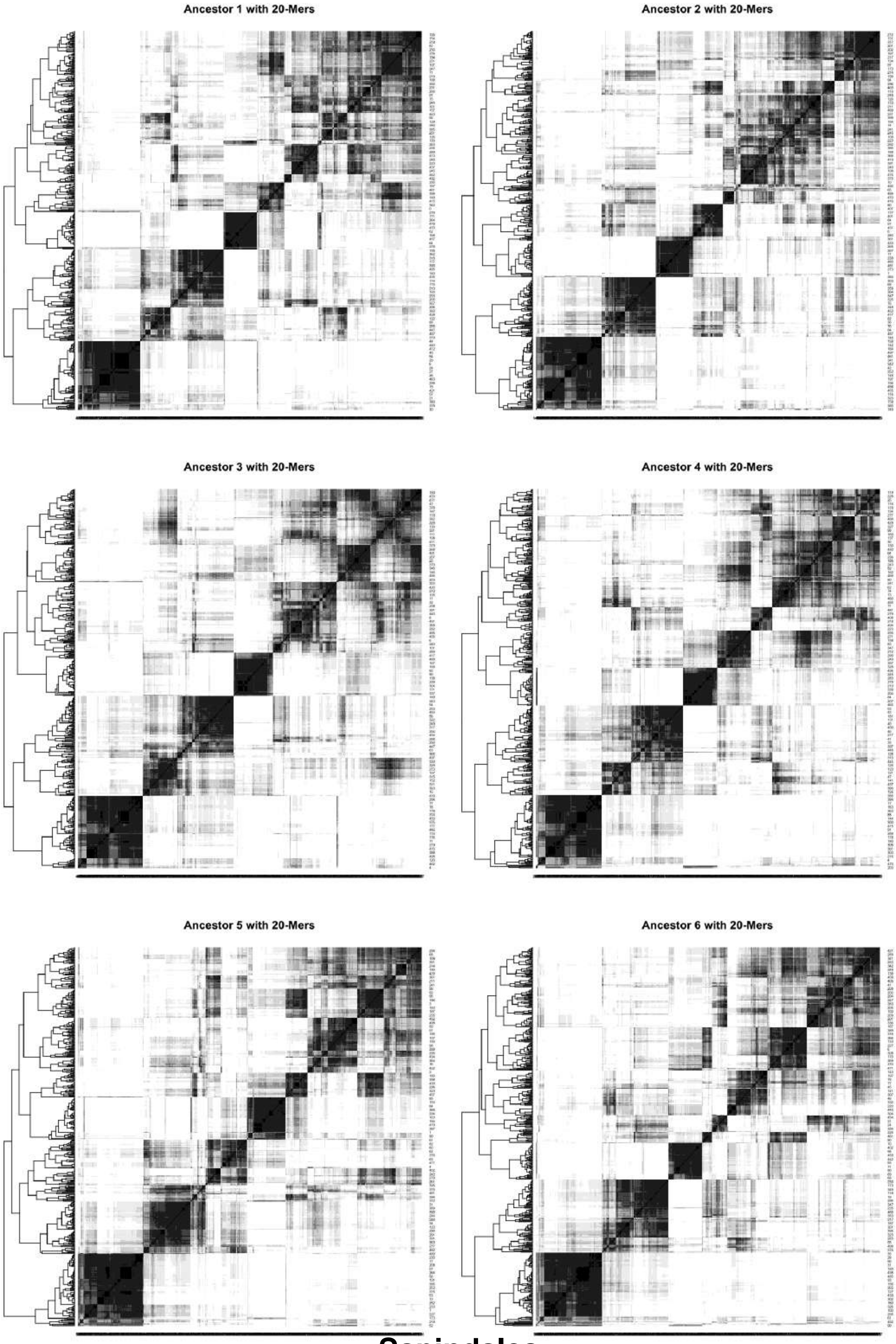
Ancestor 1 with 20-Mers
Ancestor 2 with 20-Mers
Ancestor 3 with 20-Mers
Ancestor 4 with 20-Mers
Ancestor 5 with 20-Mers
Ancestor 6 with 20-Mers

Sapindales

Ancestor 1 with 20-Mers

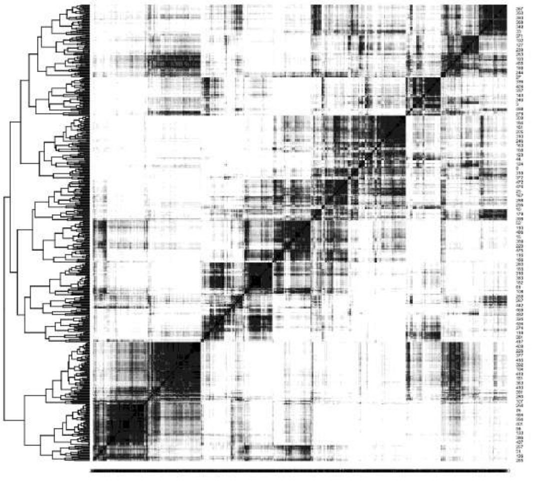

Ancestor 2 with 20-Mers

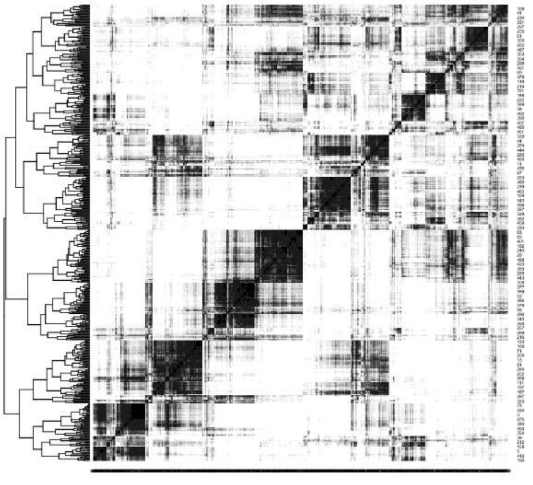

Ancestor 3 with 20-Mers

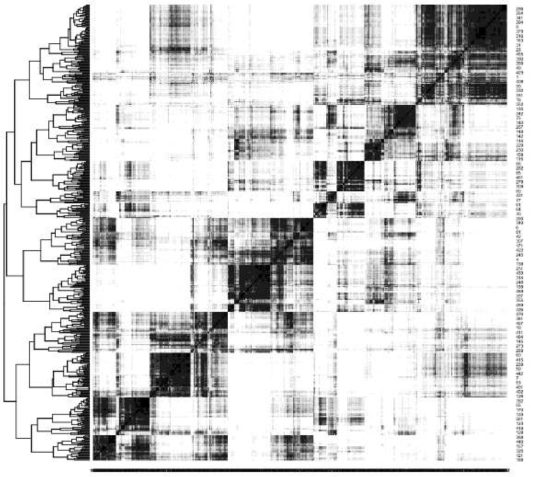

Ancestor 4 with 20-Mers

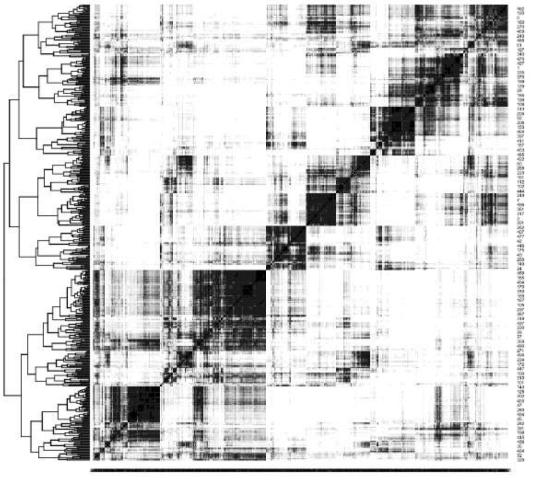

Asterales

Ancestor 1 with 40-Mers

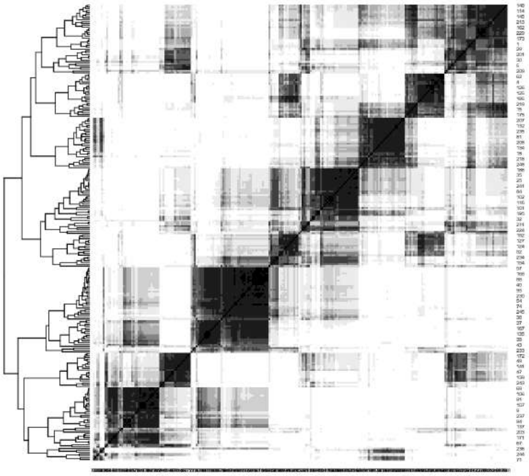

Ancestor 2 with 40-Mers

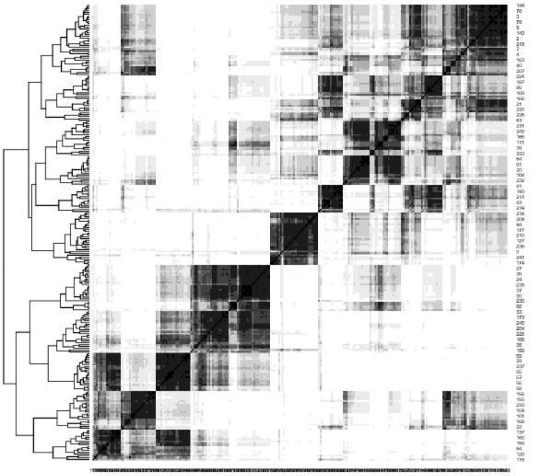

Ancestor 3 with 40-Mers

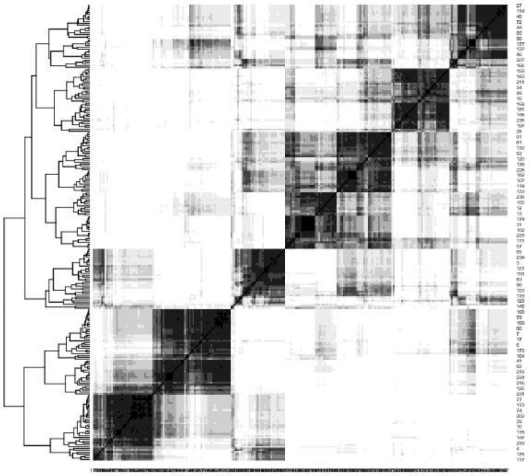

Ancestor 4 with 40-Mers

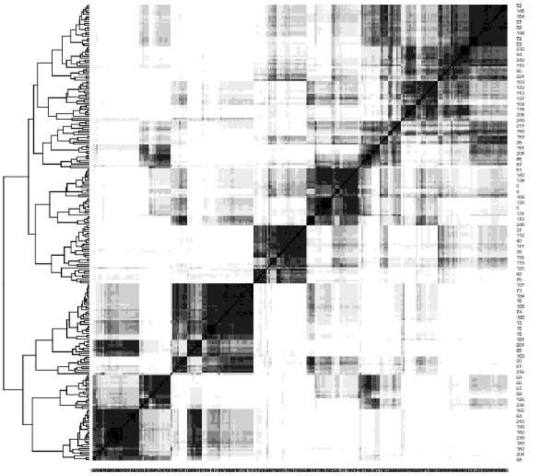

Gentianales

Ancestor 1 with 20-Mers

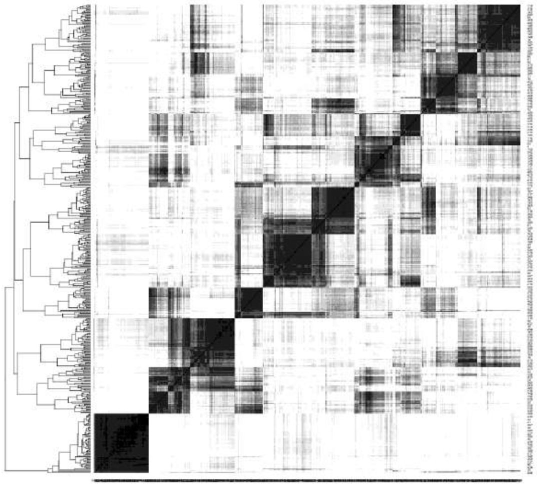

Ancestor 2 with 20-Mers

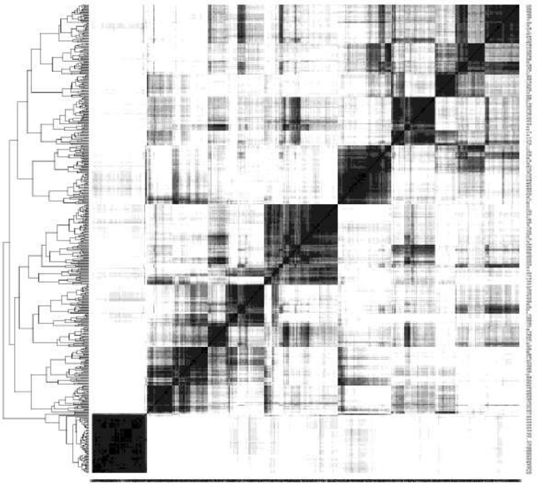

Ancestor 3 with 20-Mers

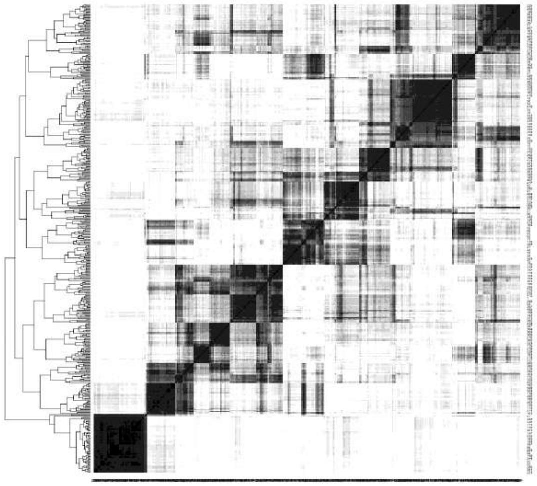

Ancestor 4 with 20-Mers

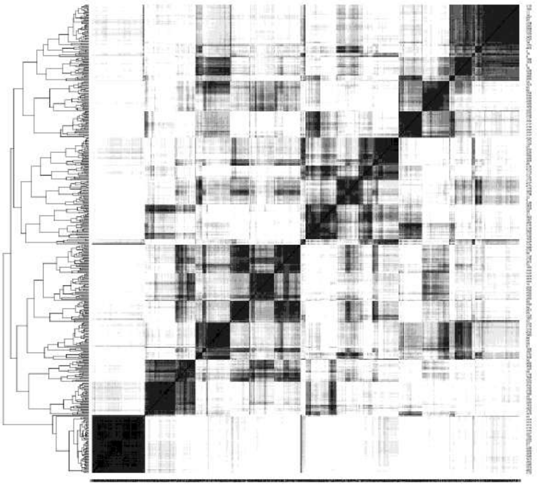

Lamiales

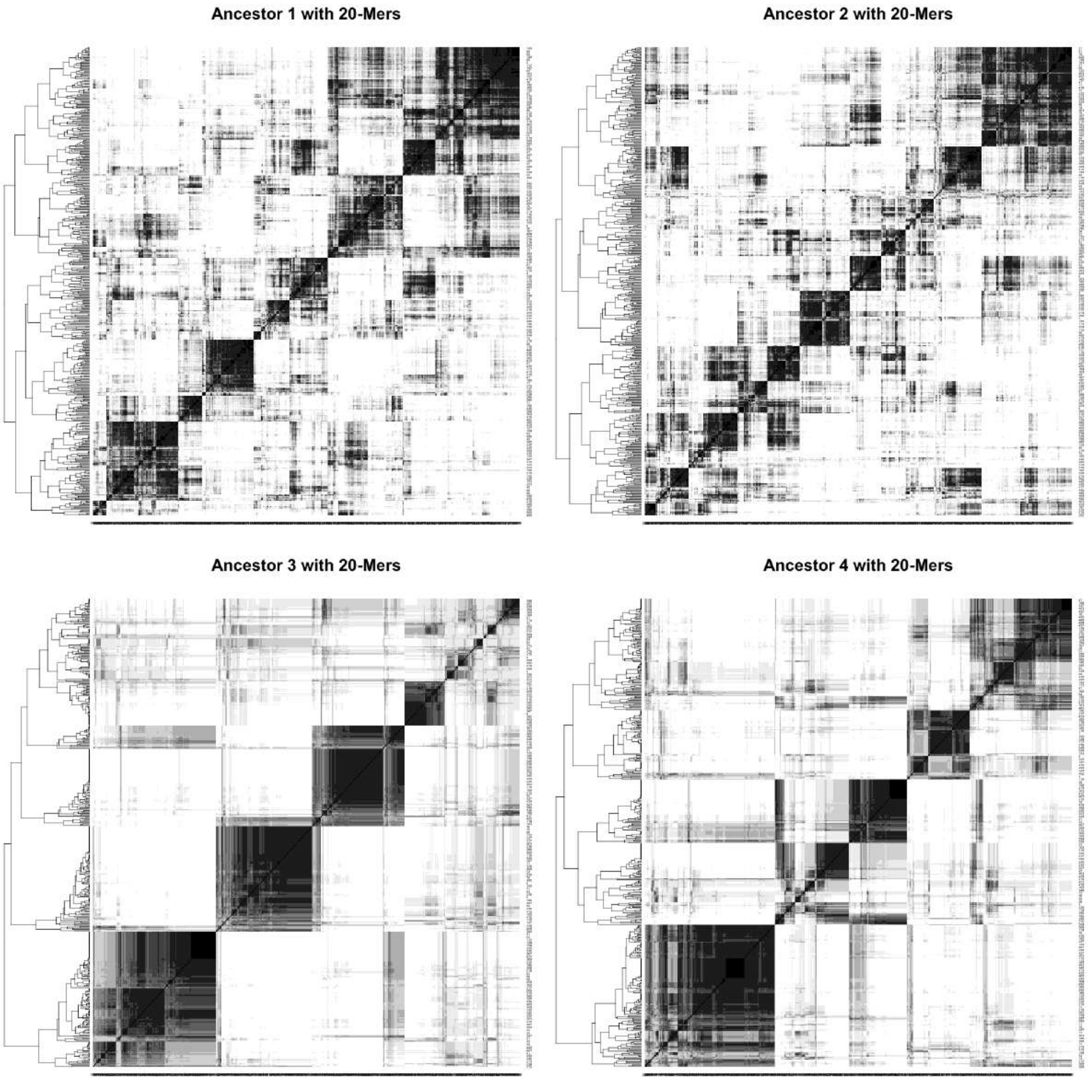
Ancestor 1 with 20-Mers
Ancestor 2 with 20-Mers
Ancestor 3 with 20-Mers
Ancestor 4 with 20-Mers

Solanales

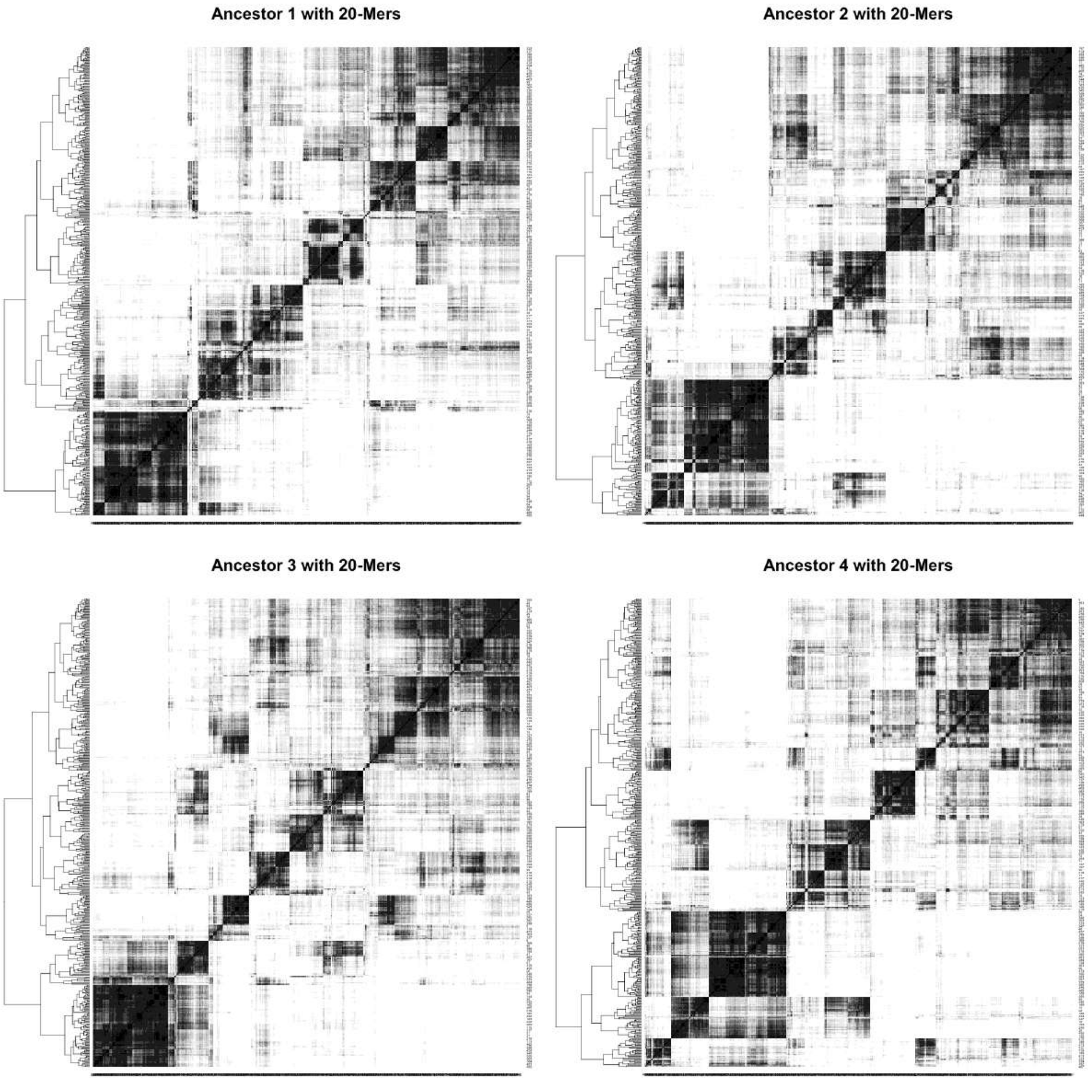
Ancestor 1 with 20-Mers
Ancestor 2 with 20-Mers
Ancestor 3 with 20-Mers
Ancestor 4 with 20-Mers

Ericales

Supplement B. Evaluation graphs. Eleven orders

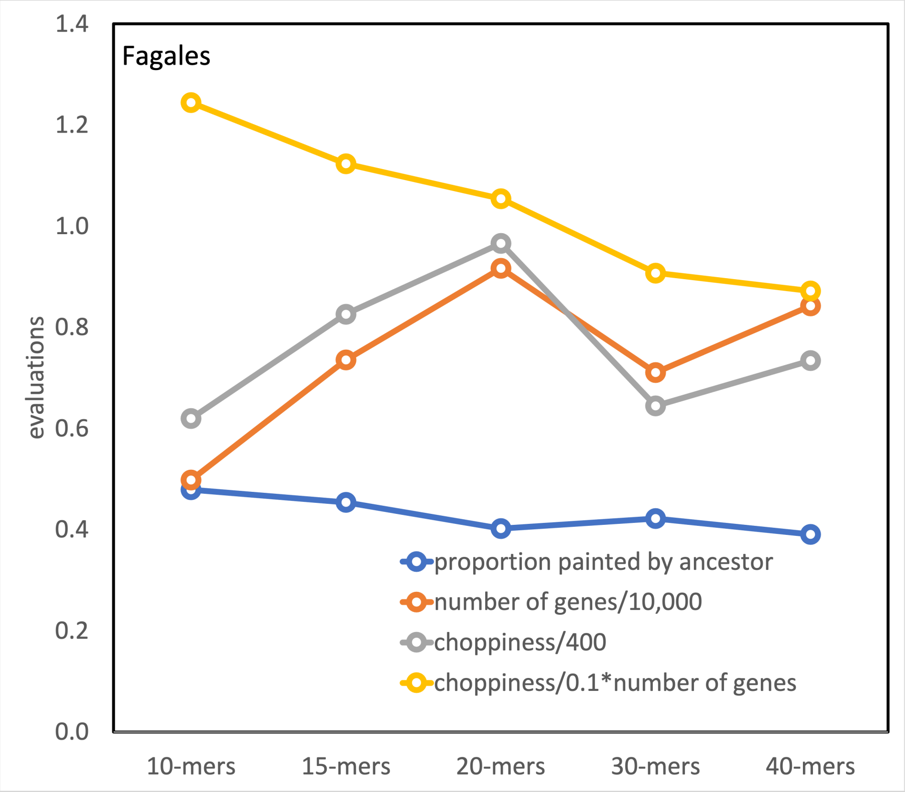

Fagales
evaluations
1.4
1.2
1.0
0.8
0.6
0.4
0.2
0.0
10-mers
15-mers
20-mers
30-mers
40-mers
proportion painted by ancestor
number of genes/10,000
choppiness/400
choppiness/0.1*number of genes

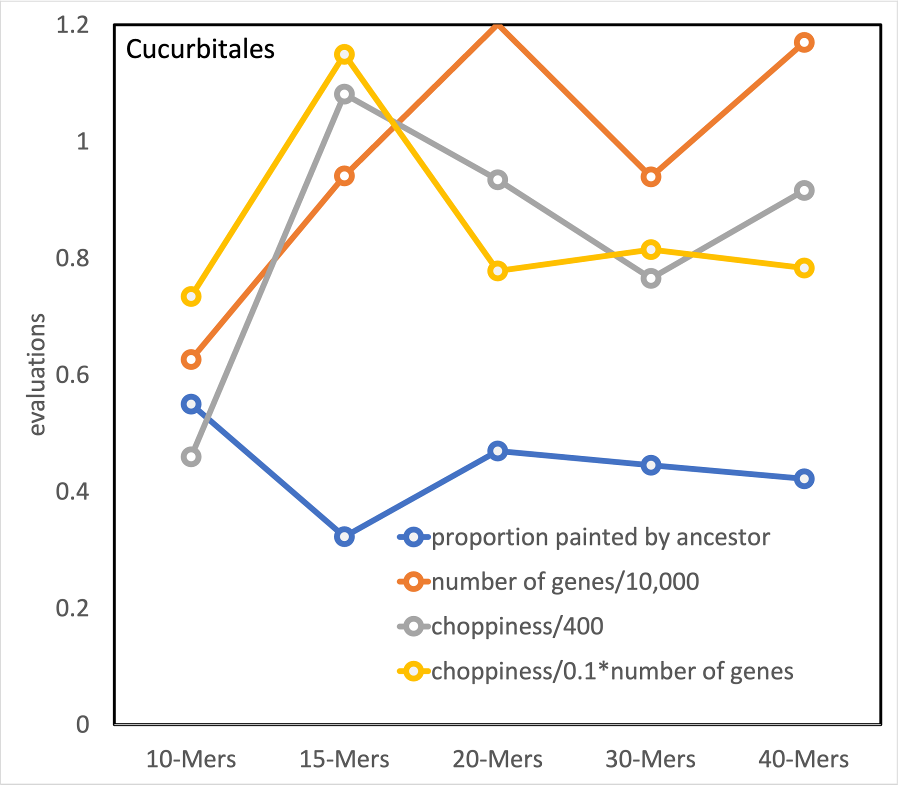
Cucurbitales
evaluations
1.2
1
0.8
0.6
0.4
0.2
0
10-Mers
15-Mers
20-Mers
30-Mers
40-Mers
proportion painted by ancestor
number of genes/10,000
choppiness/400
choppiness/0.1*number of genes

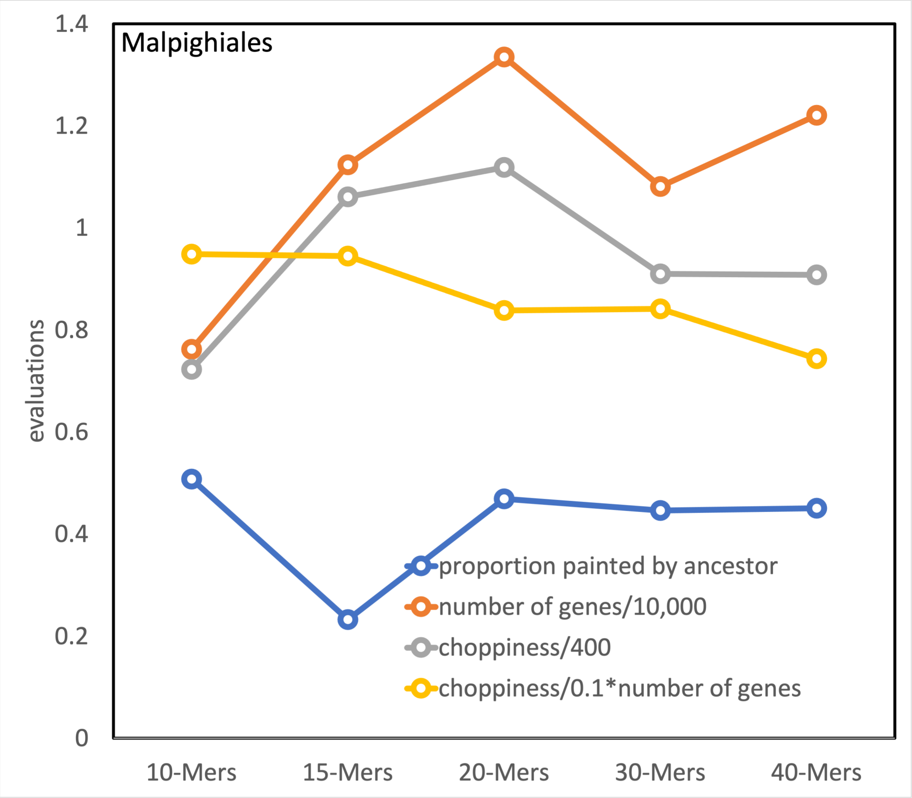

Malpighiales
evaluations
1.4
1.2
1
0.8
0.6
0.4
0.2
0
10-Mers
15-Mers
20-Mers
30-Mers
40-Mers
proportion painted by ancestor
number of genes/10,000
choppiness/400
choppiness/0.1*number of genes

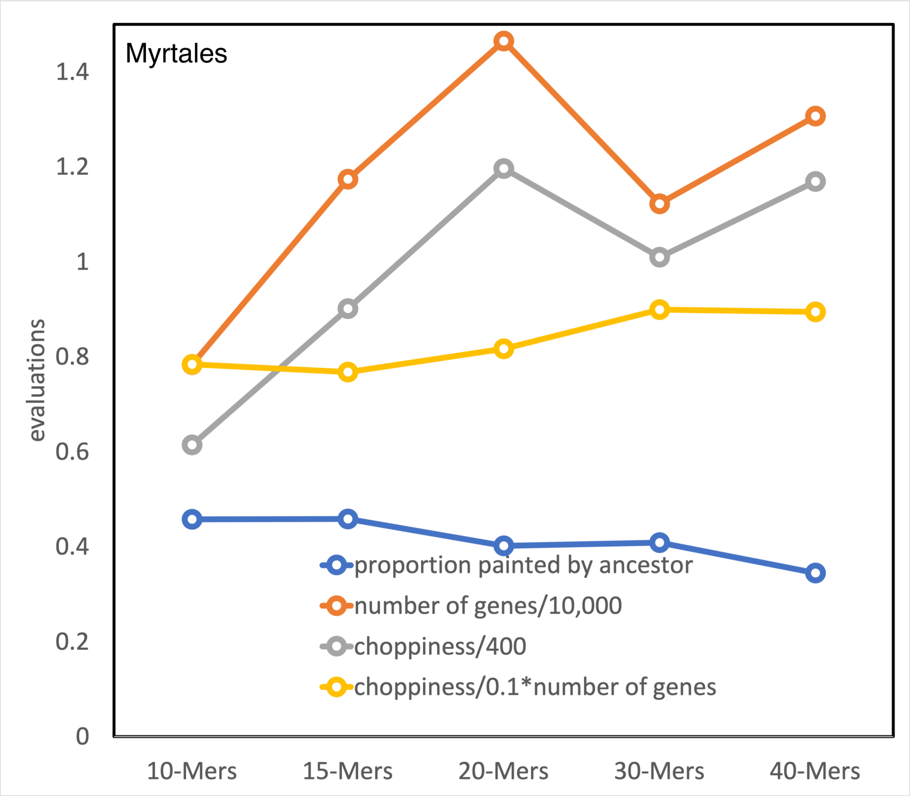

Myrtales
evaluations
1.4
1.2
1
0.8
0.6
0.4
0.2
0
10-Mers
15-Mers
20-Mers
30-Mers
40-Mers
proportion painted by ancestor
number of genes/10,000
choppiness/400
choppiness/0.1*number of genes

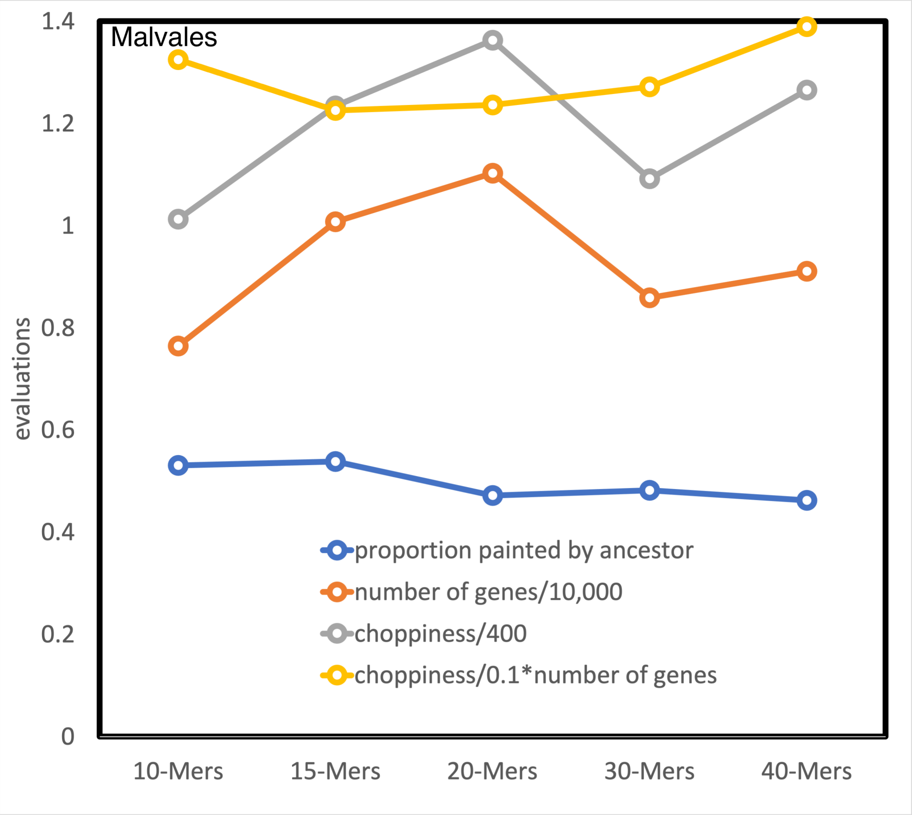

Malvales
evaluations
1.4
1.2
1
0.8
0.6
0.4
0.2
0
10-Mers
15-Mers
20-Mers
30-Mers
40-Mers
proportion painted by ancestor
number of genes/10,000
choppiness/400
choppiness/0.1*number of genes

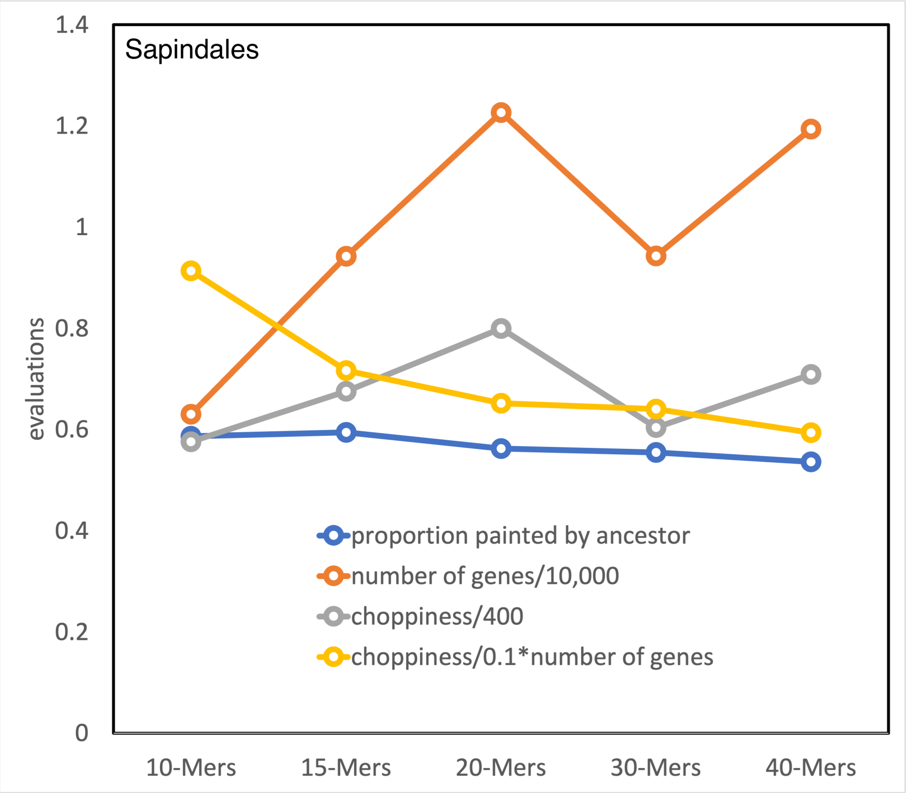

Sapindales
evaluations
1.4
1.2
1
0.8
0.6
0.4
0.2
0
10-Mers
15-Mers
20-Mers
30-Mers
40-Mers
proportion painted by ancestor
number of genes/10,000
choppiness/400
choppiness/0.1*number of genes

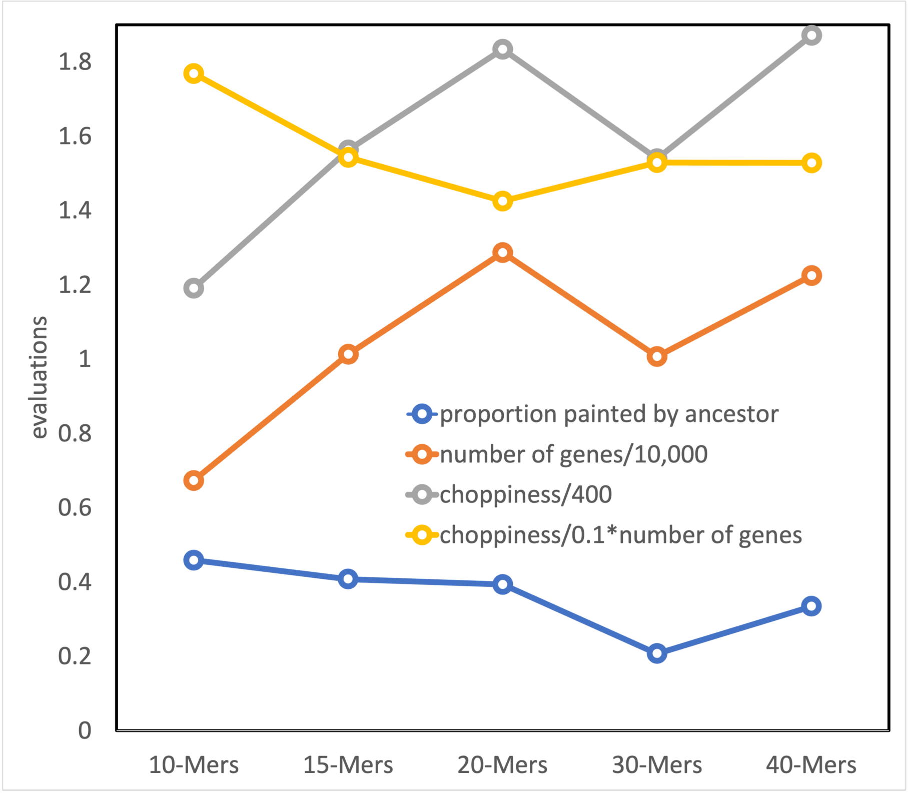

1.8
1.6
1.4
1.2
1
0.8
0.6
0.4
0.2
0
evaluations
proportion painted by ancestor
number of genes/10,000
choppiness/400
choppiness/0.1*number of genes
10-Mers
15-Mers
20-Mers
30-Mers
40-Mers

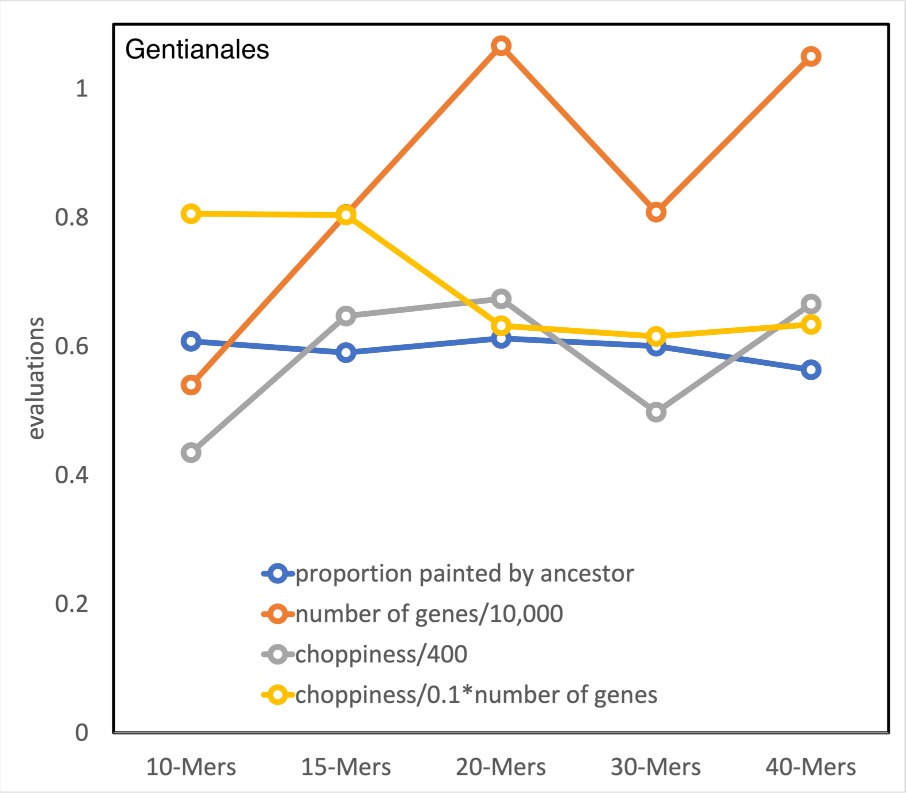
Gentianales
evaluations
1
0.8
0.6
0.4
0.2
0
10-Mers
15-Mers
20-Mers
30-Mers
40-Mers
proportion painted by ancestor
number of genes/10,000
choppiness/400
choppiness/0.1*number of genes

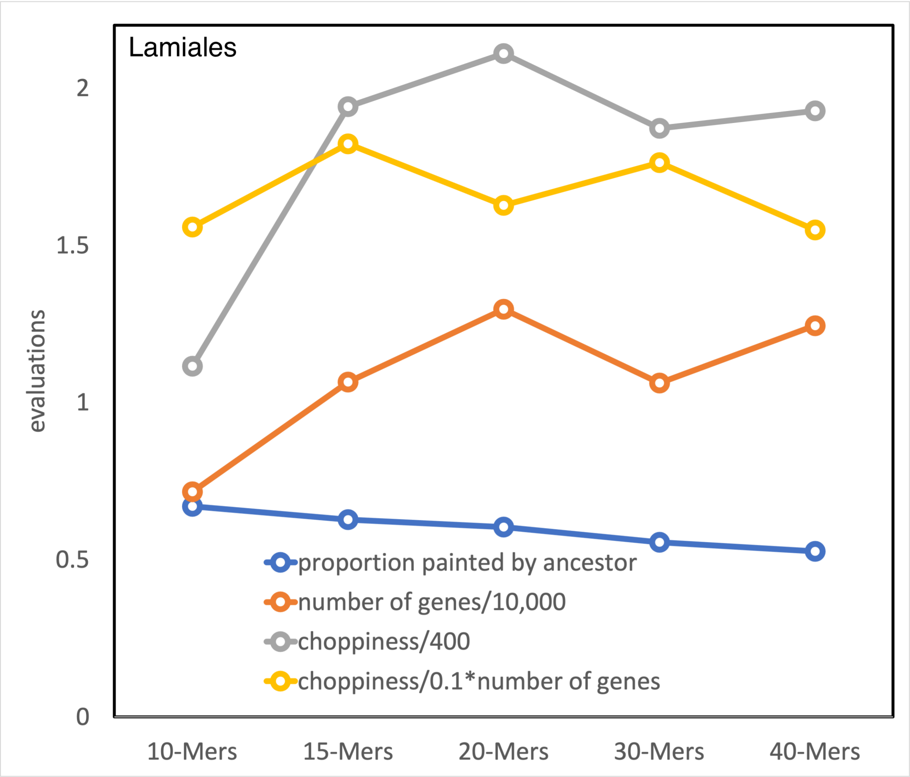
Lamiales
evaluations
2
1.5
1
0.5
0
10-Mers
15-Mers
20-Mers
30-Mers
40-Mers
proportion painted by ancestor
number of genes/10,000
choppiness/400
choppiness/0.1*number of genes

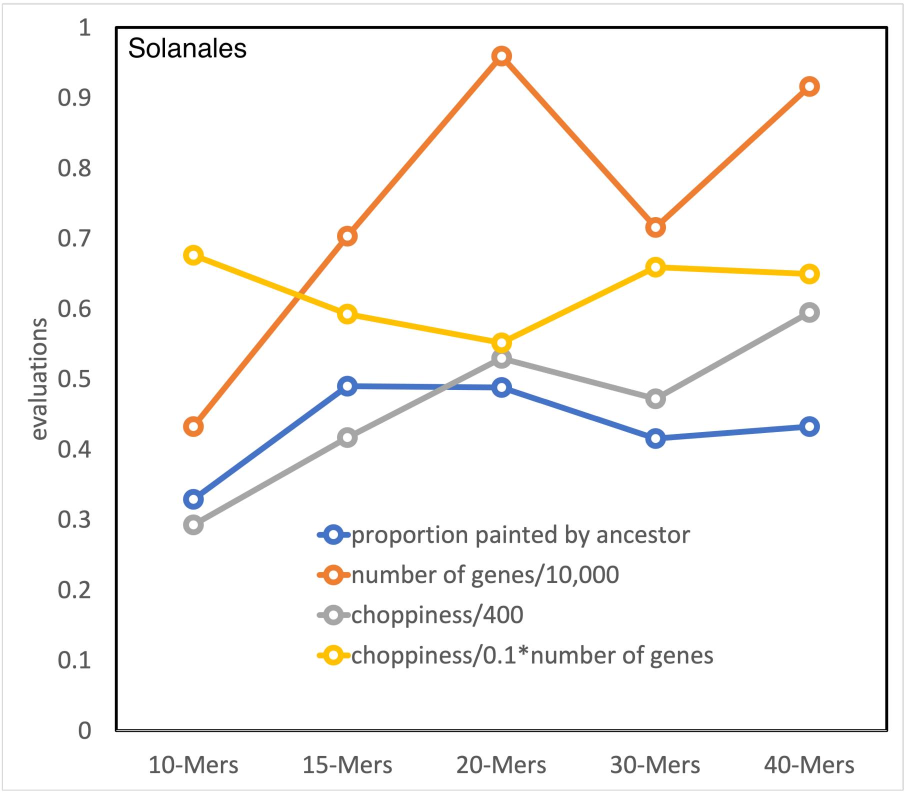
Solanales
evaluations
1
0.9
0.8
0.7
0.6
0.5
0.4
0.3
0.2
0.1
0
10-Mers
15-Mers
20-Mers
30-Mers
40-Mers
proportion painted by ancestor
number of genes/10,000
choppiness/400
choppiness/0.1*number of genes

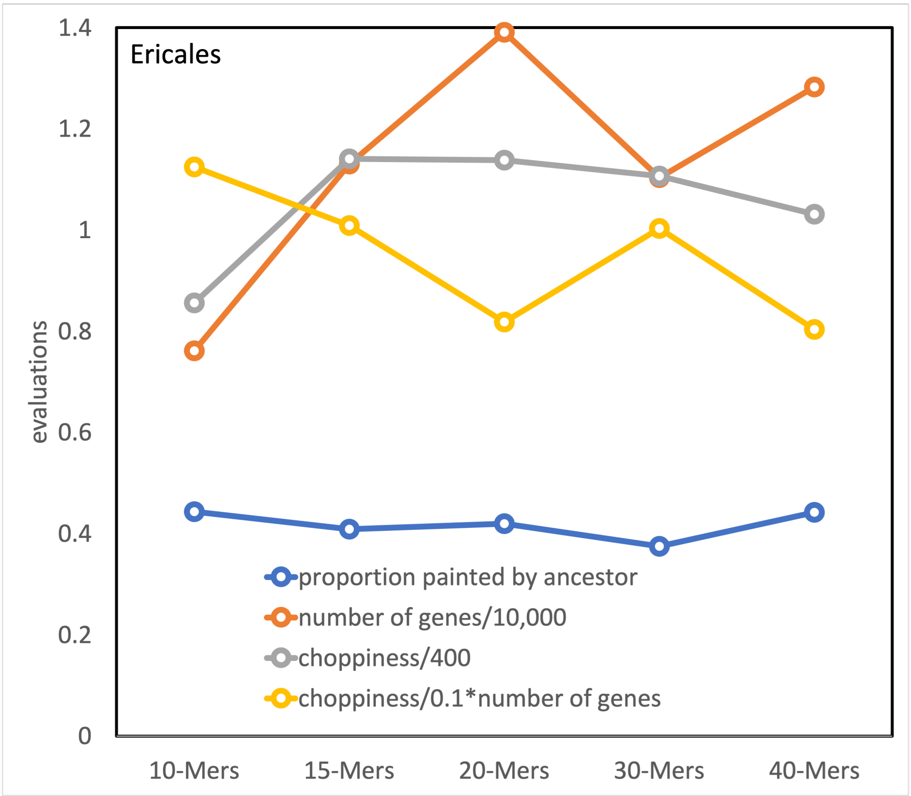
Ericales
evaluations
1.4
1.2
1
0.8
0.6
0.4
0.2
0
10-Mers
15-Mers
20-Mers
30-Mers
40-Mers
proportion painted by ancestor
number of genes/10,000
choppiness/400
choppiness/0.1*number of genes

Supplement C. Gap statistics. For $g = 20$ each ancestor, eleven orders

Gap statistics plots also available for 15-mers, 30-mers and 40-mers.

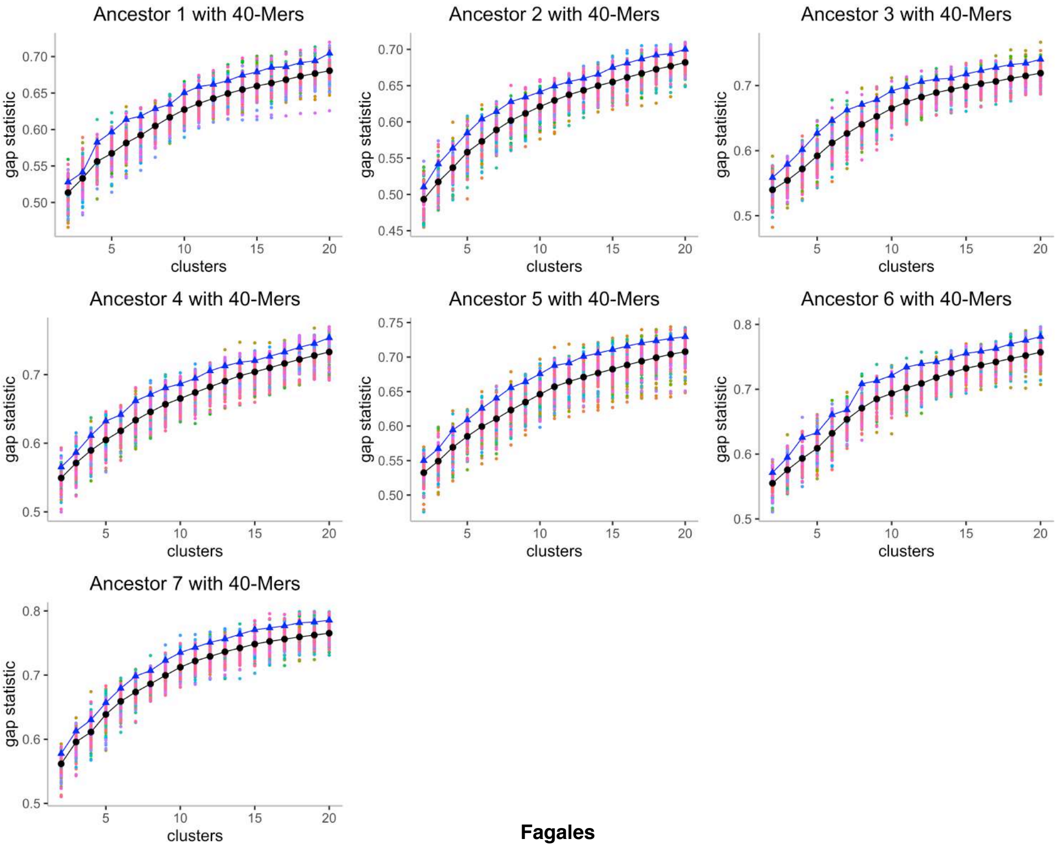
Ancestor 1 with 40-Mers
Ancestor 2 with 40-Mers
Ancestor 3 with 40-Mers
Ancestor 4 with 40-Mers
Ancestor 5 with 40-Mers
Ancestor 6 with 40-Mers
Ancestor 7 with 40-Mers
gap statistic
clusters

Fagales

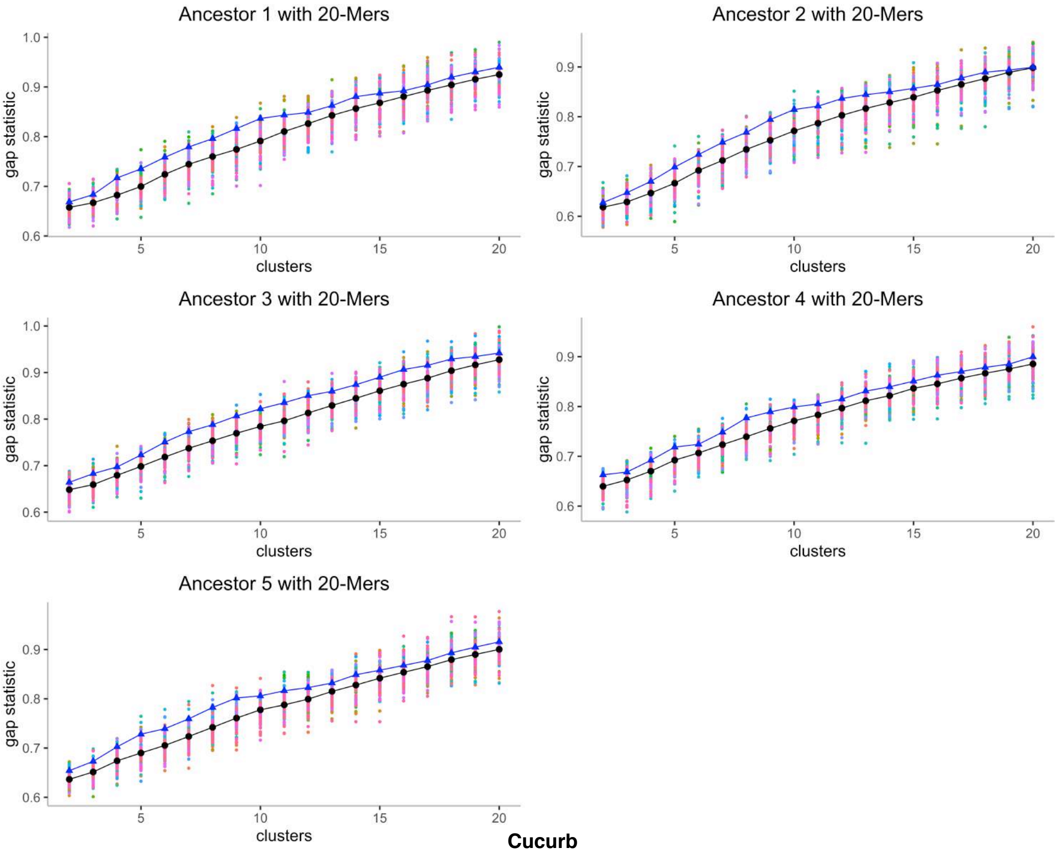

Ancestor 1 with 20-Mers
Ancestor 2 with 20-Mers
Ancestor 3 with 20-Mers
Ancestor 4 with 20-Mers
Ancestor 5 with 20-Mers
gap statistic
clusters

Cucurb

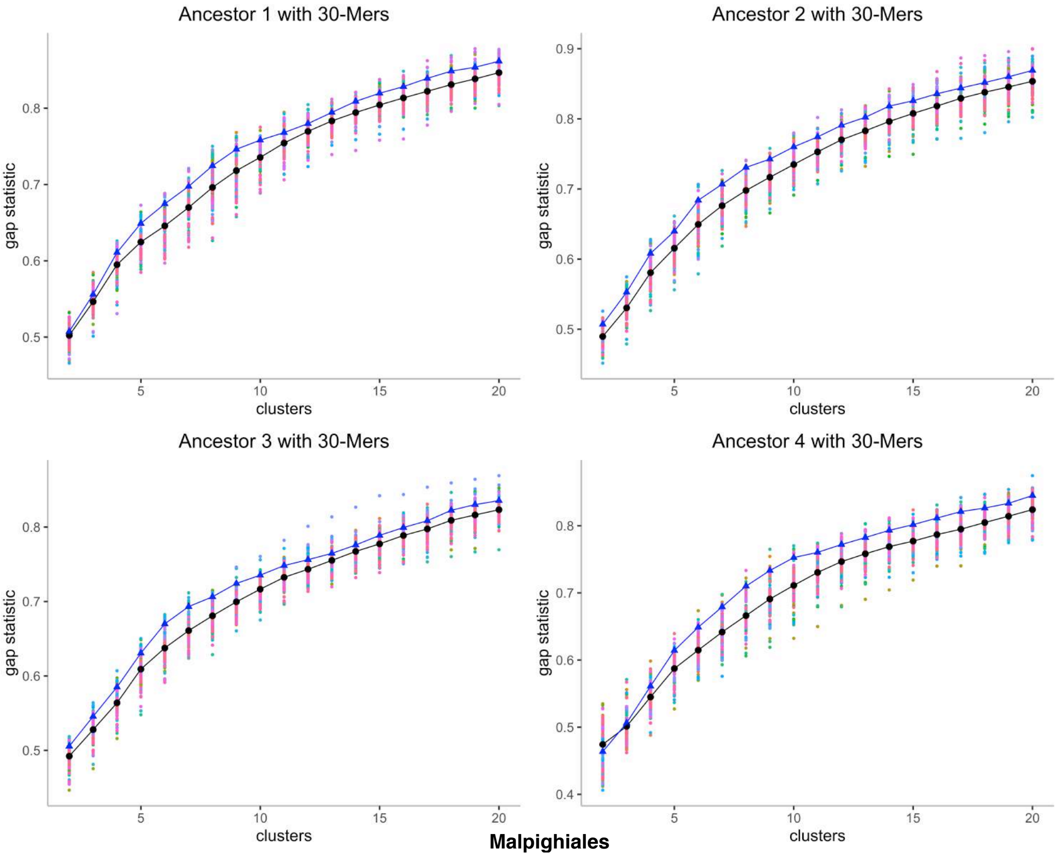

Malpighiales

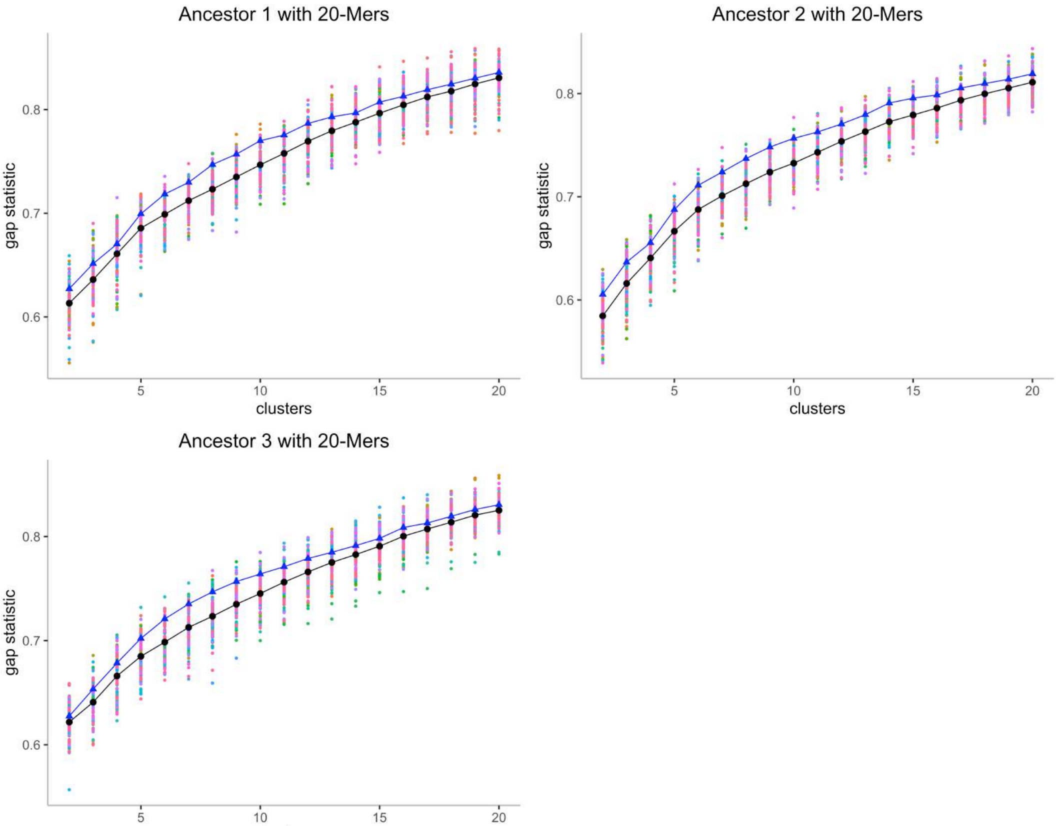
Ancestor 1 with 20-Mers
gap statistic
clusters
Ancestor 2 with 20-Mers
gap statistic
clusters
Ancestor 3 with 20-Mers
gap statistic
clusters

Myrtales

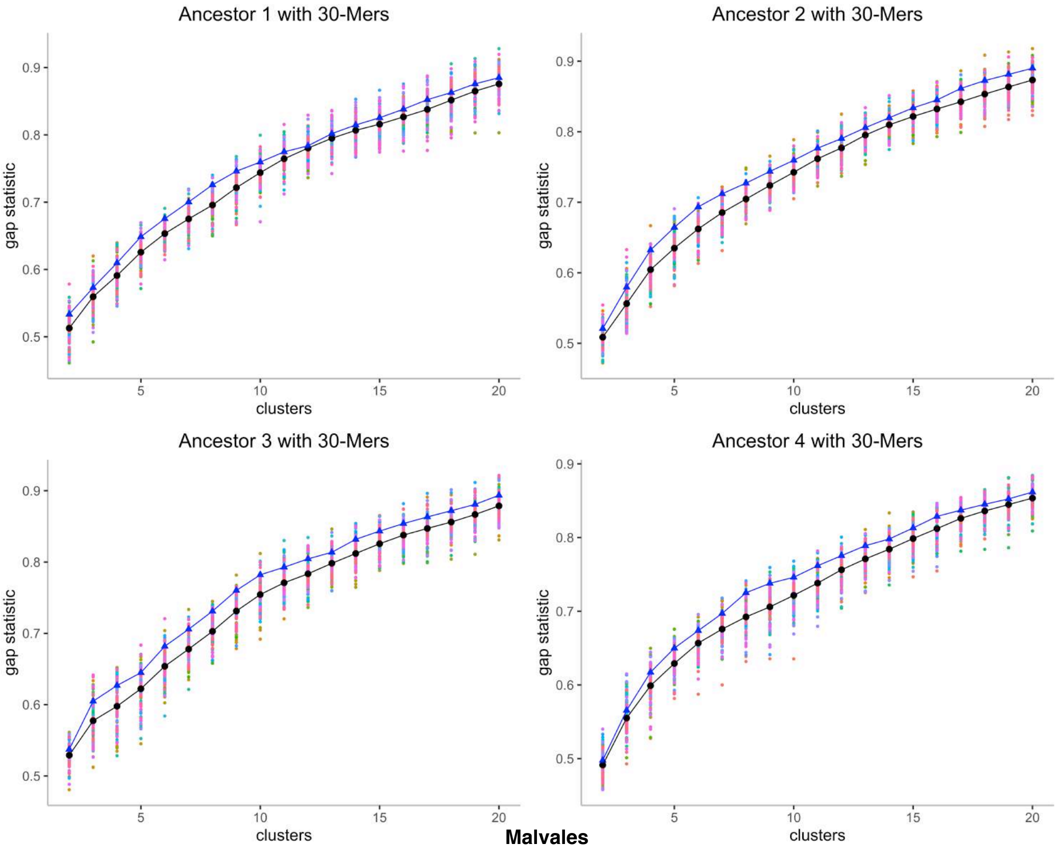

Ancestor 1 with 30-Mers
Ancestor 2 with 30-Mers
Ancestor 3 with 30-Mers
Ancestor 4 with 30-Mers
gap statistic
clusters
0.5
0.6
0.7
0.8
0.9
5
10
15
20

Malvales

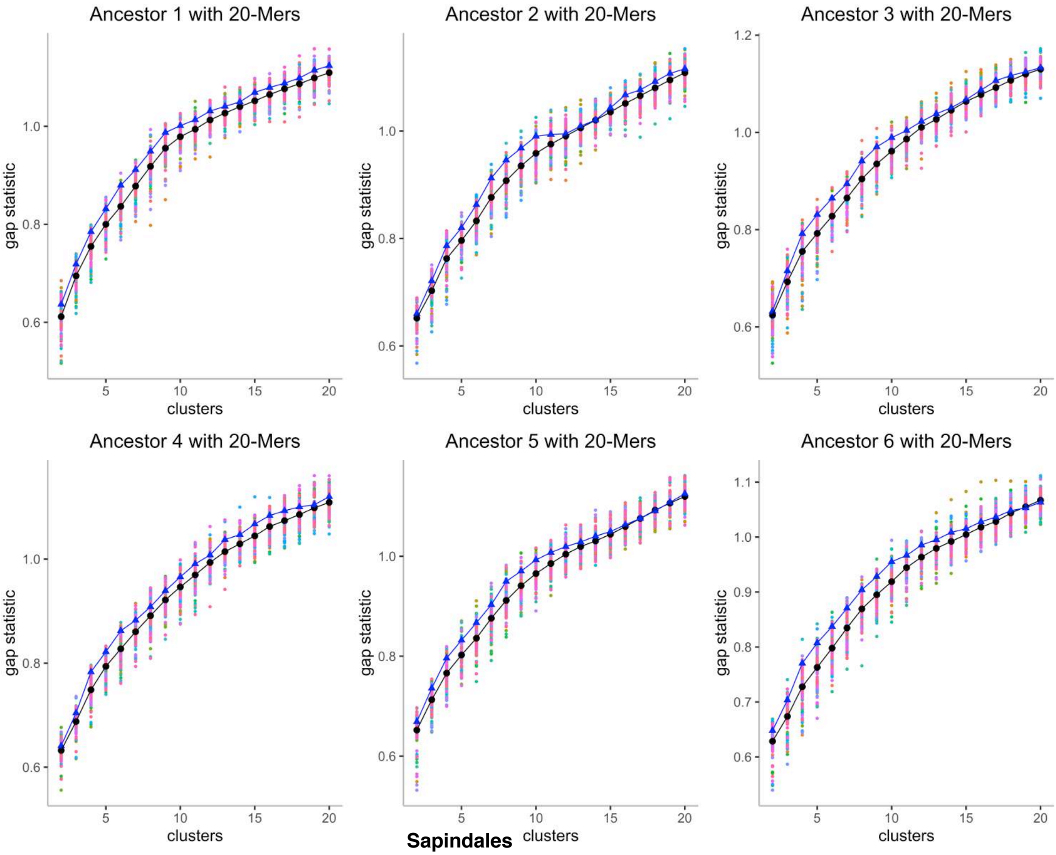
Ancestor 1 with 20-Mers
Ancestor 2 with 20-Mers
Ancestor 3 with 20-Mers
Ancestor 4 with 20-Mers
Ancestor 5 with 20-Mers
Ancestor 6 with 20-Mers
gap statistic
clusters

Sapindales

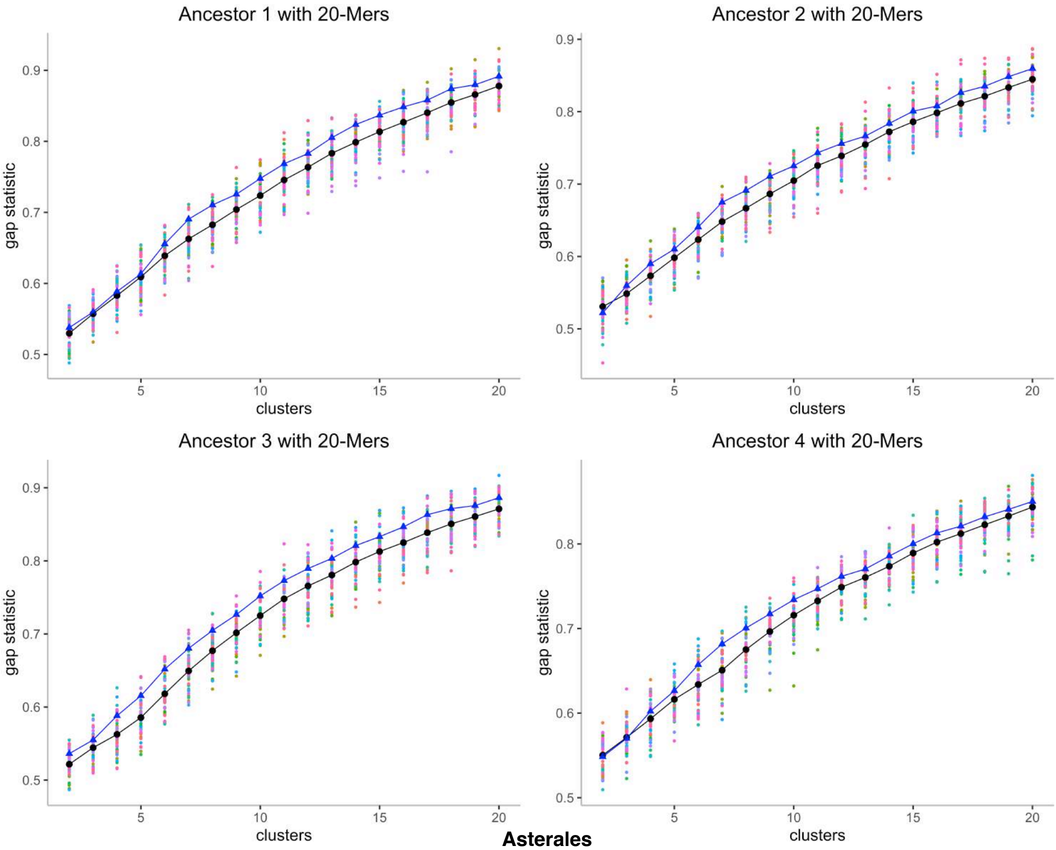
Ancestor 1 with 20-Mers
Ancestor 2 with 20-Mers
Ancestor 3 with 20-Mers
Ancestor 4 with 20-Mers
gap statistic
clusters
0.5
0.6
0.7
0.8
0.9
5
10
15
20

Asterales

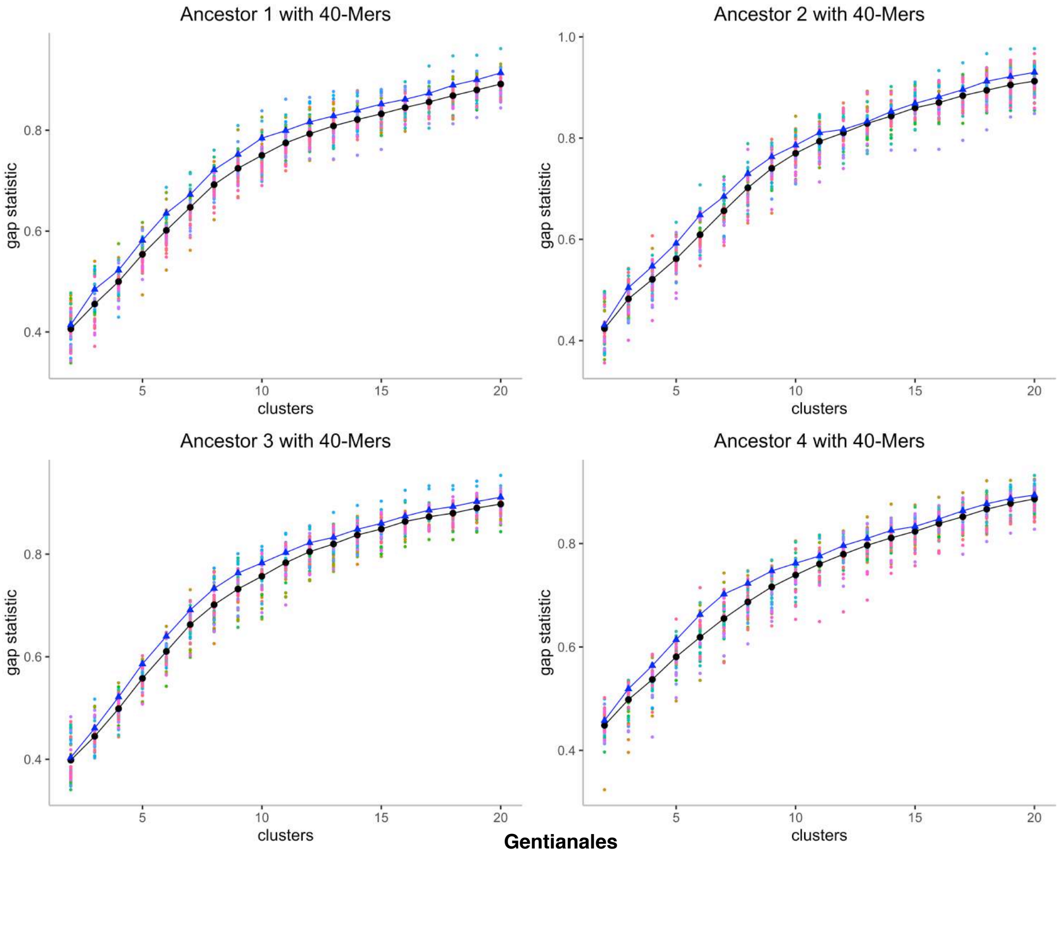

Gentianales

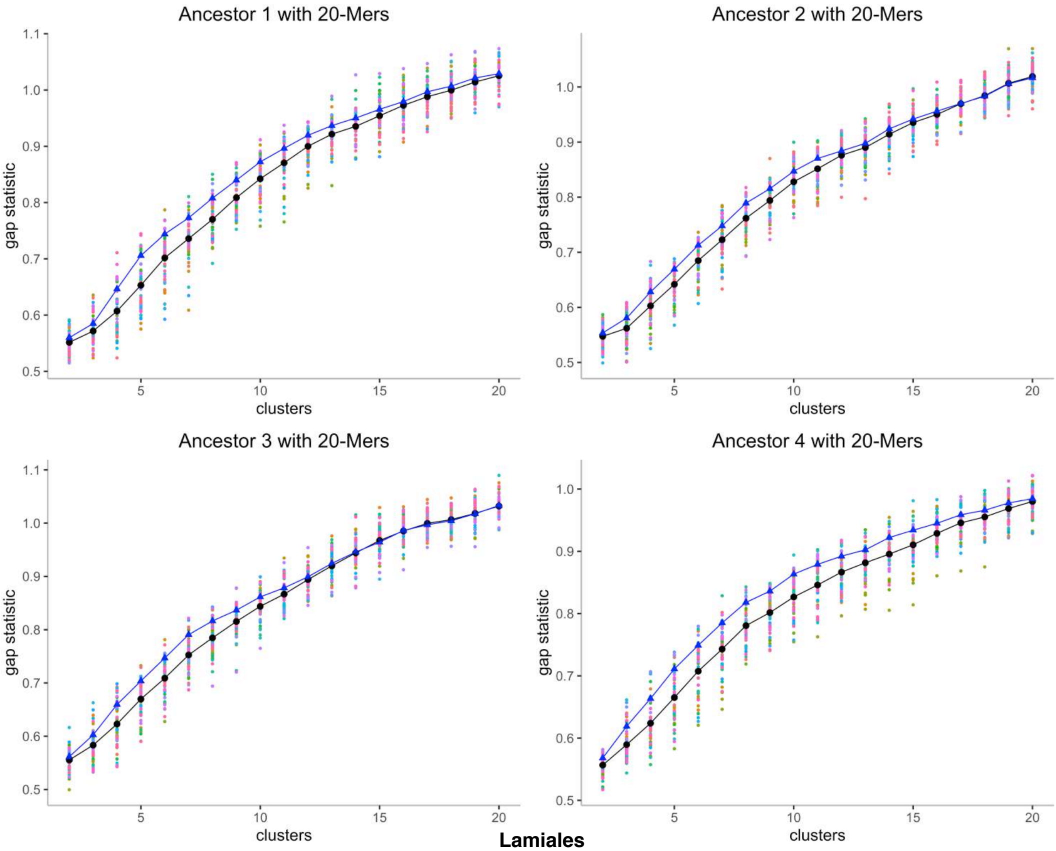
Ancestor 1 with 20-Mers
Ancestor 2 with 20-Mers
Ancestor 3 with 20-Mers
Ancestor 4 with 20-Mers
gap statistic
clusters

Lamiales

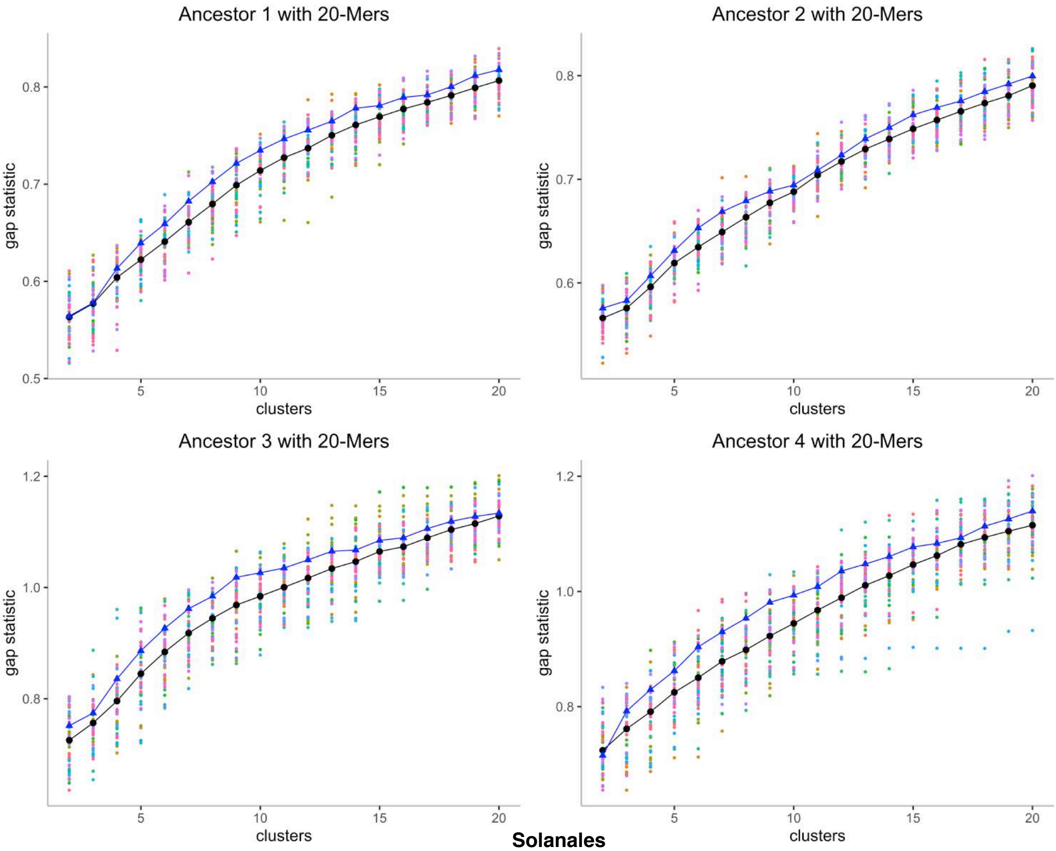

Ancestor 1 with 20-Mers
Ancestor 2 with 20-Mers
Ancestor 3 with 20-Mers
Ancestor 4 with 20-Mers
gap statistic
clusters

Solanales

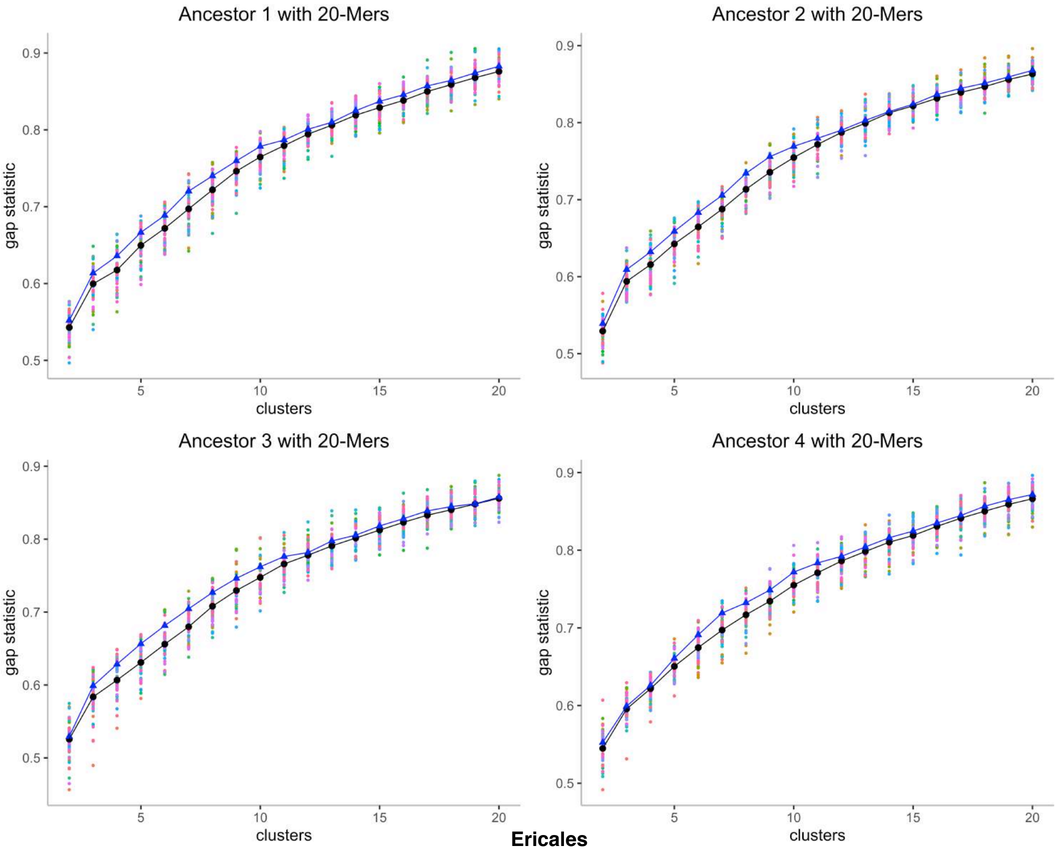
Ancestor 1 with 20-Mers
Ancestor 2 with 20-Mers
Ancestor 3 with 20-Mers
Ancestor 4 with 20-Mers
gap statistic
clusters
0.5
0.6
0.7
0.8
0.9
5
10
15
20

Ericales

Supplement D. Painted extant genomes $g = 20$

Missing genomes where chromosome-level assemblies not available. Paintings = available for 15-mers, 20-mers, 30-mers and 40-mers.

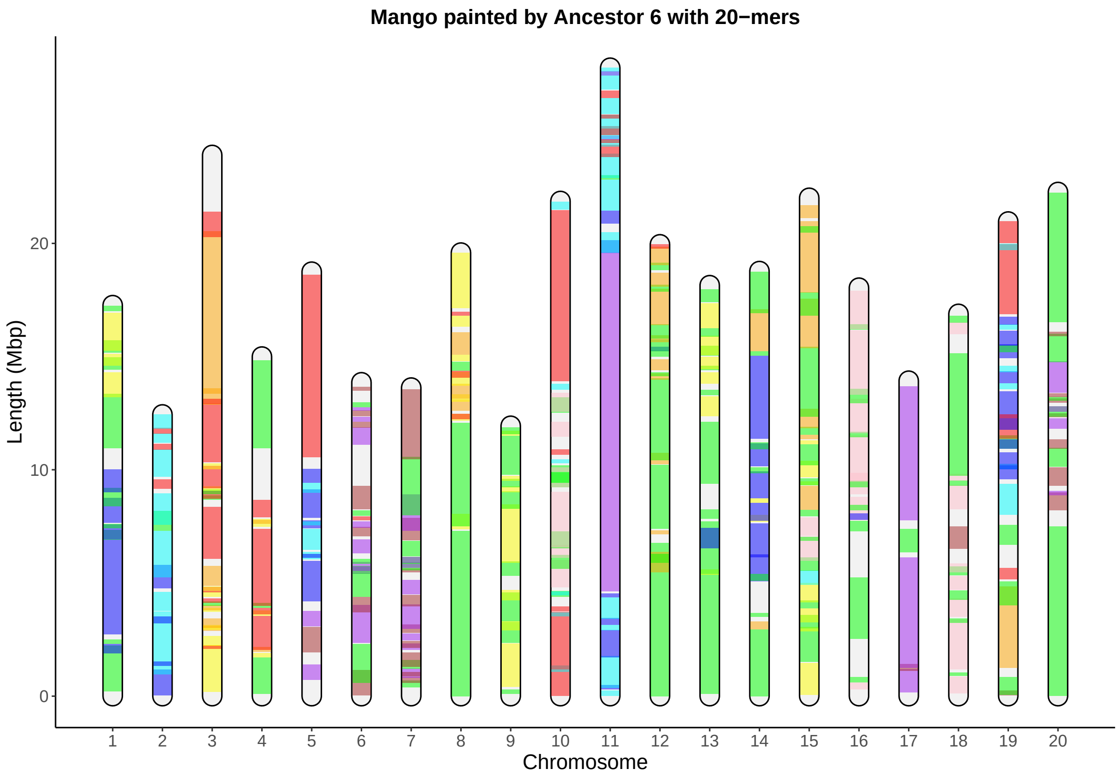

Mango painted by Ancestor 6 with 20−mers
Length (Mbp)
0
10
20
Chromosome
1
2
3
4
5
6
7
8
9
10
11
12
13
14
15
16
17
18
19
20

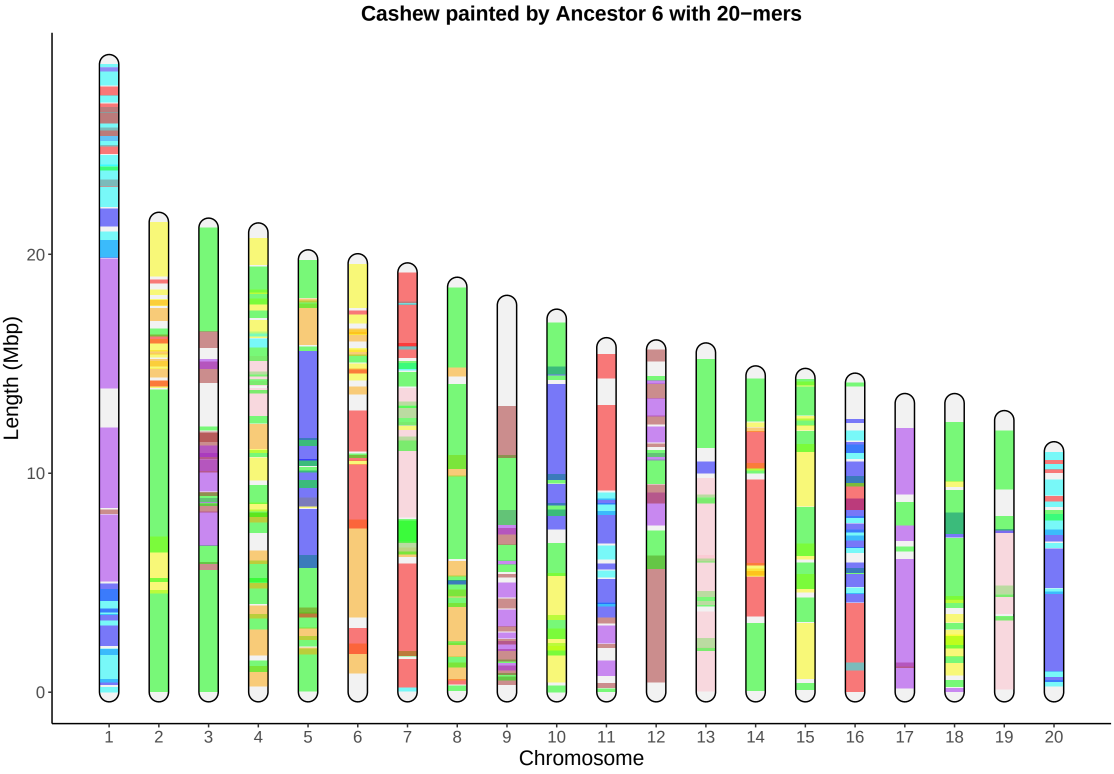
Cashew painted by Ancestor 6 with 20−mers
Length (Mbp)
0
10
20
Chromosome
1
2
3
4
5
6
7
8
9
10
11
12
13
14
15
16
17
18
19
20

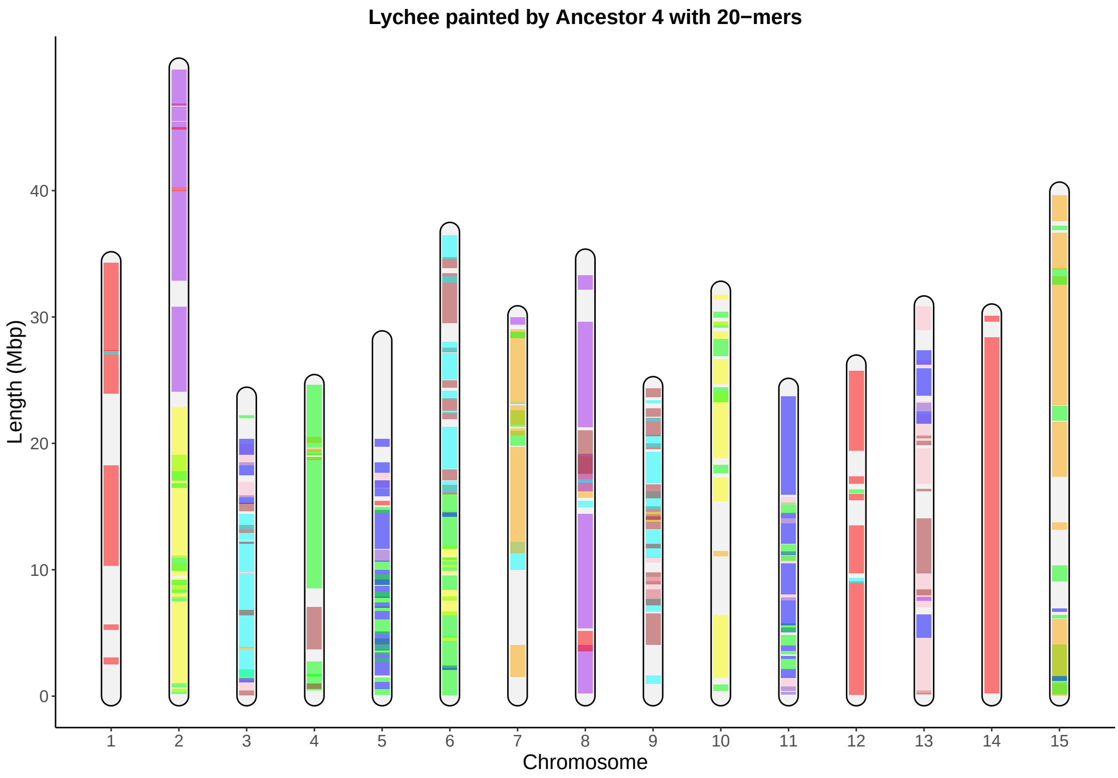

Lychee painted by Ancestor 4 with 20−mers
Length (Mbp)
0
10
20
30
40
1
2
3
4
5
6
7
8
9
10
11
12
13
14
15
Chromosome

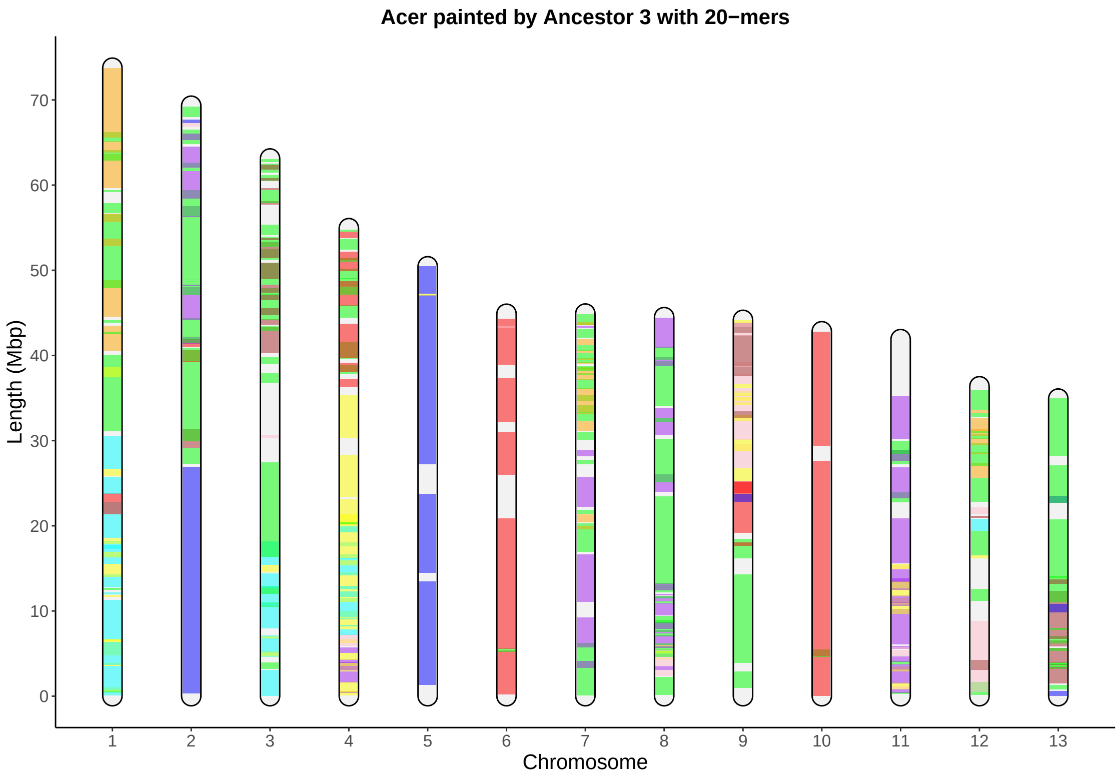

Acer painted by Ancestor 3 with 20−mers
Length (Mbp)
0
10
20
30
40
50
60
70
1
2
3
4
5
6
7
8
9
10
11
12
13
Chromosome

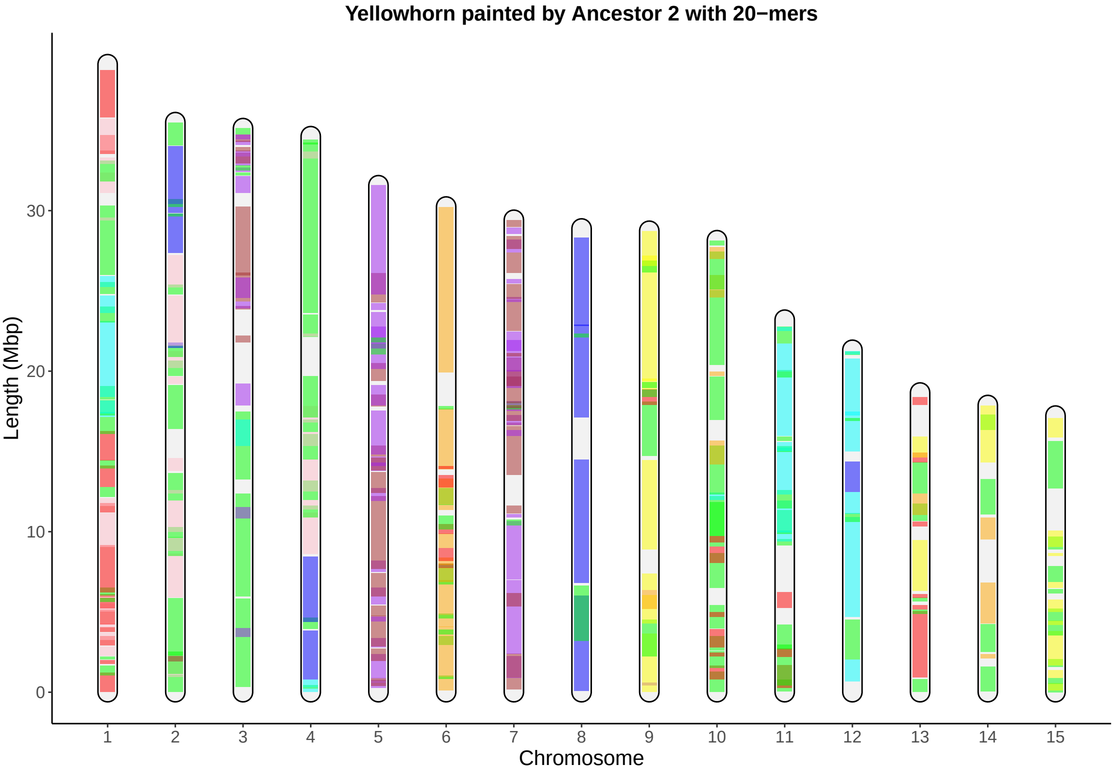

Yellowhorn painted by Ancestor 2 with 20−mers
Length (Mbp)
0
10
20
30
1
2
3
4
5
6
7
8
9
10
11
12
13
14
15
Chromosome

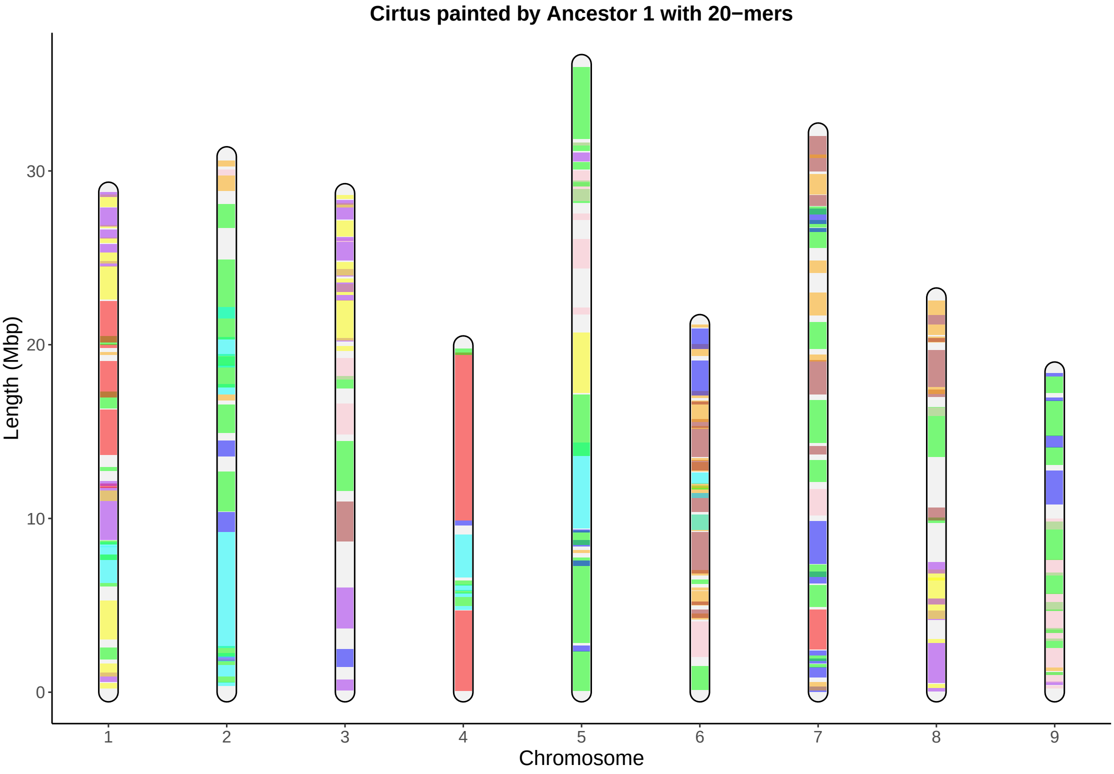
Cirtus painted by Ancestor 1 with 20−mers
Length (Mbp)
0
10
20
30
Chromosome
1
2
3
4
5
6
7
8
9

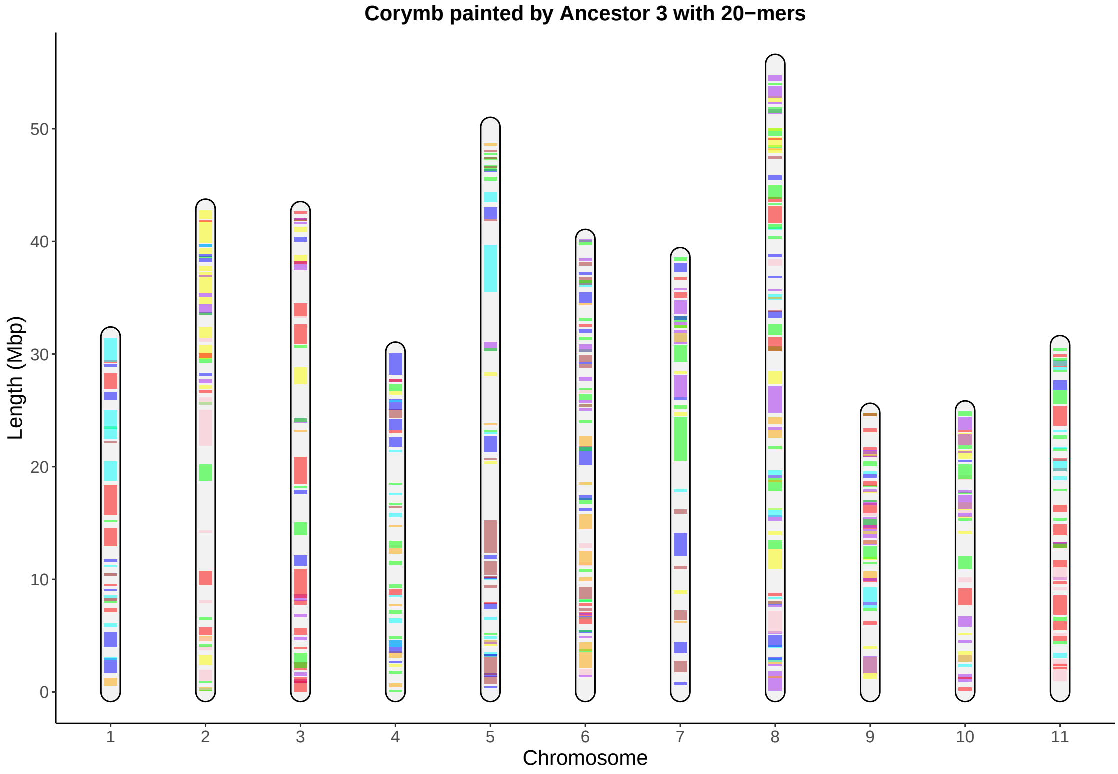

Corymb painted by Ancestor 3 with 20−mers
Length (Mbp)
0
10
20
30
40
50
1
2
3
4
5
6
7
8
9
10
11
Chromosome

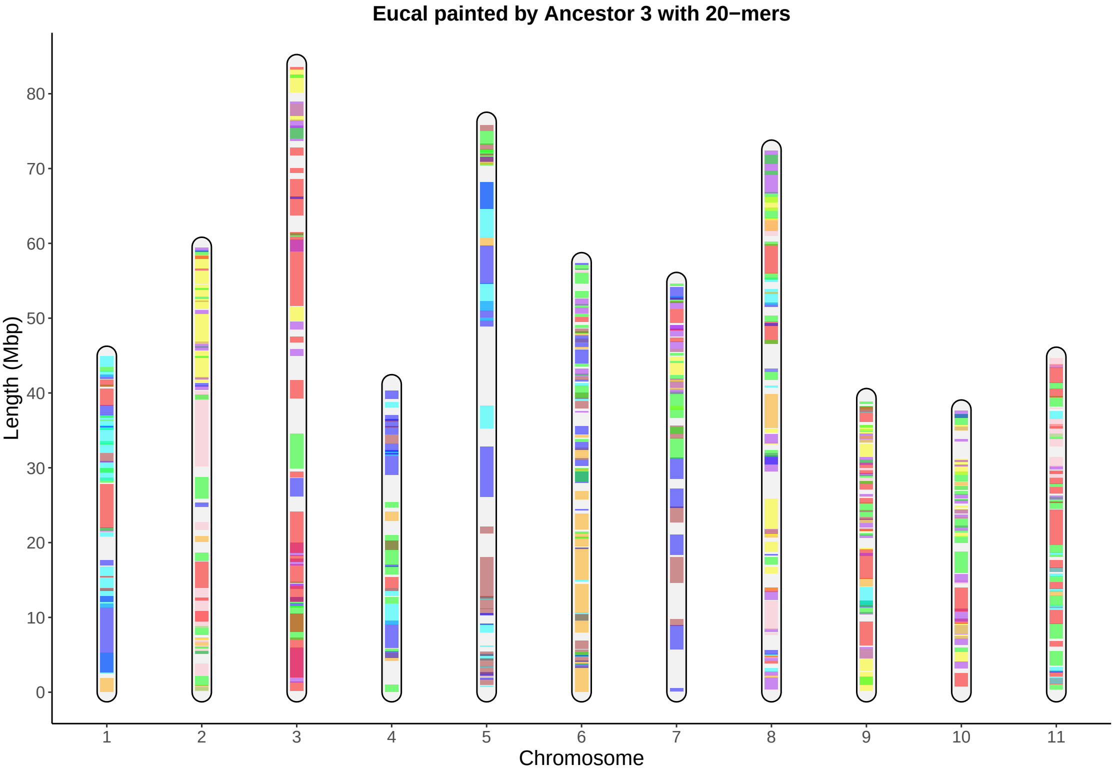

Eucal painted by Ancestor 3 with 20−mers
Length (Mbp)
0
10
20
30
40
50
60
70
80
1
2
3
4
5
6
7
8
9
10
11
Chromosome

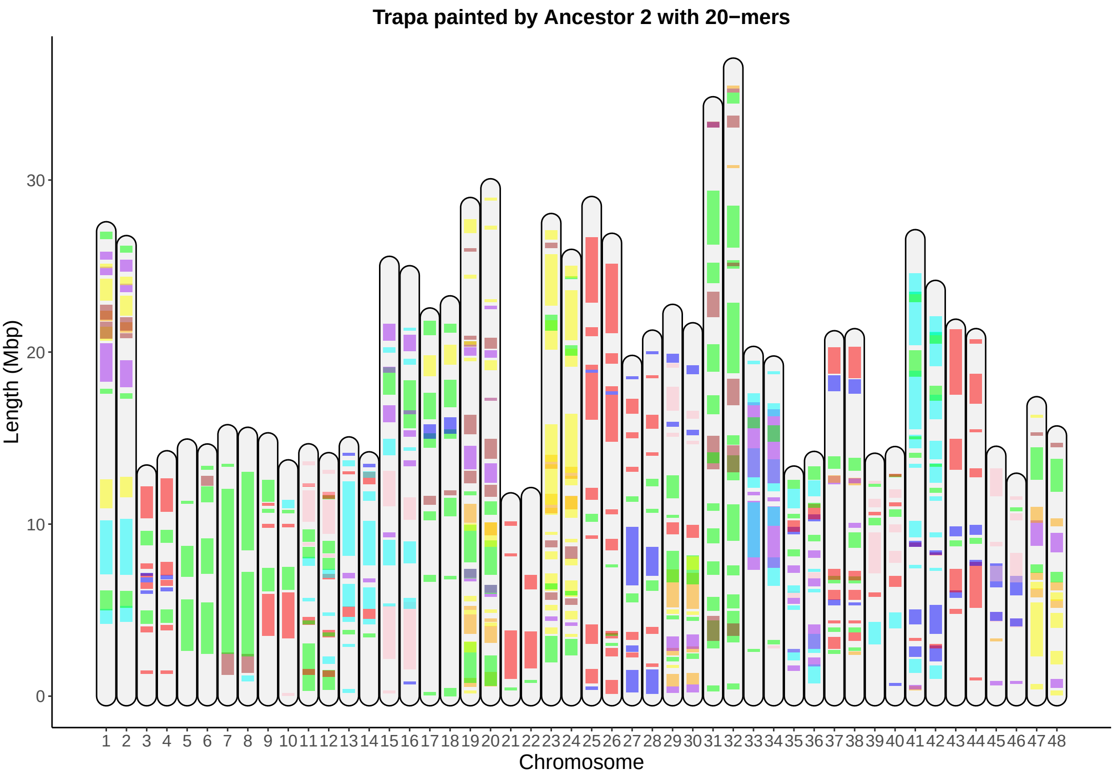

Trapa painted by Ancestor 2 with 20−mers
Length (Mbp)
0
10
20
30
Chromosome
1 2 3 4 5 6 7 8 9 10 11 12 13 14 15 16 17 18 19 20 21 22 23 24 25 26 27 28 29 30 31 32 33 34 35 36 37 38 39 40 41 42 43 44 45 46 47 48

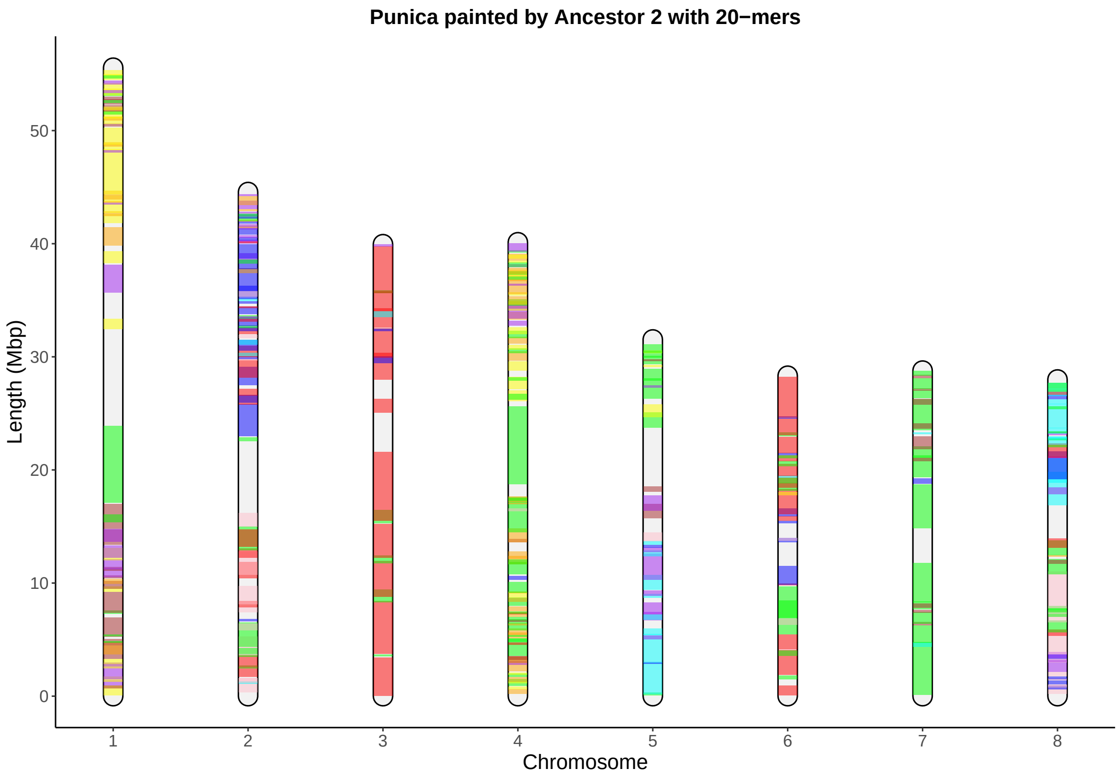

Punica painted by Ancestor 2 with 20−mers
Length (Mbp)
0
10
20
30
40
50
1
2
3
4
5
6
7
8
Chromosome

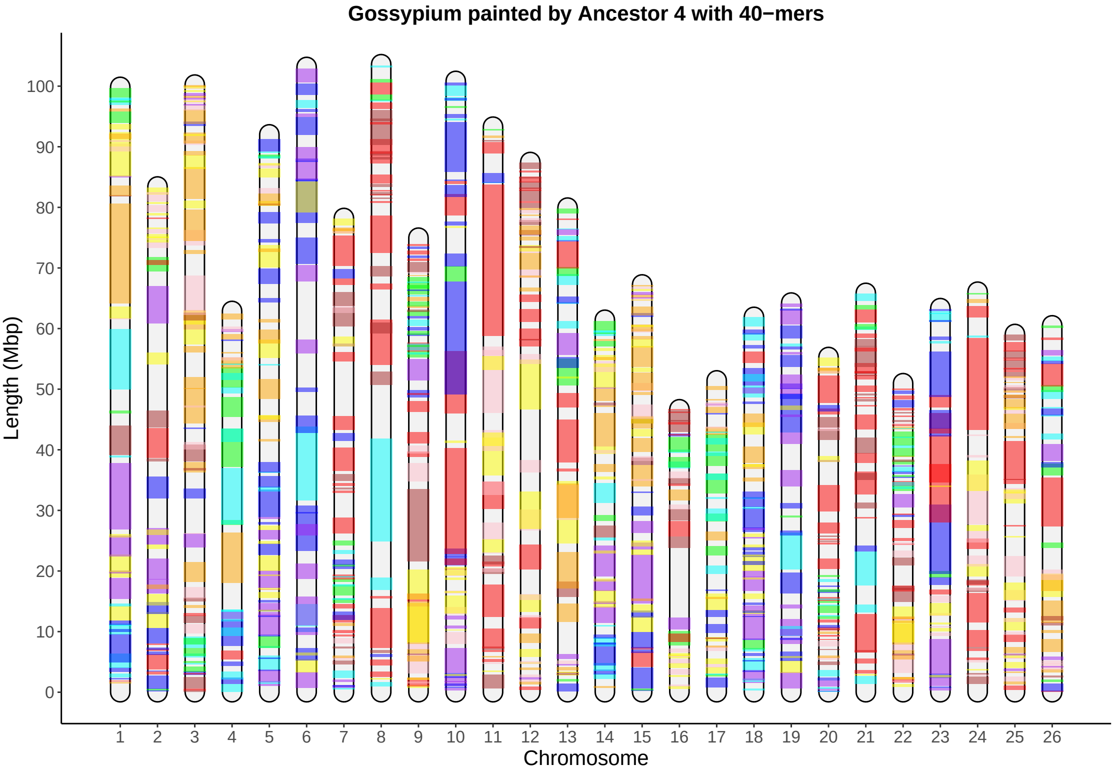
Gossypium painted by Ancestor 4 with 40−mers
Length (Mbp)
0
10
20
30
40
50
60
70
80
90
100
Chromosome
1
2
3
4
5
6
7
8
9
10
11
12
13
14
15
16
17
18
19
20
21
22
23
24
25
26

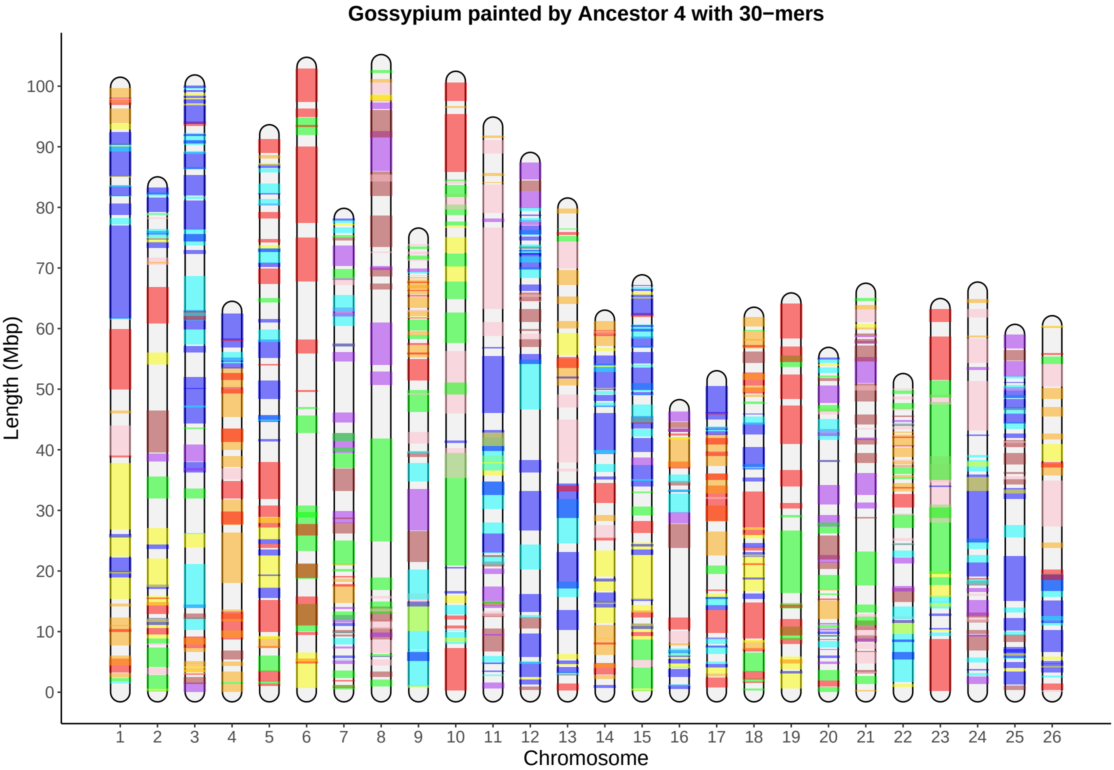
Gossypium painted by Ancestor 4 with 30−mers
Length (Mbp)
100
90
80
70
60
50
40
30
20
10
0
1 2 3 4 5 6 7 8 9 10 11 12 13 14 15 16 17 18 19 20 21 22 23 24 25 26
Chromosome

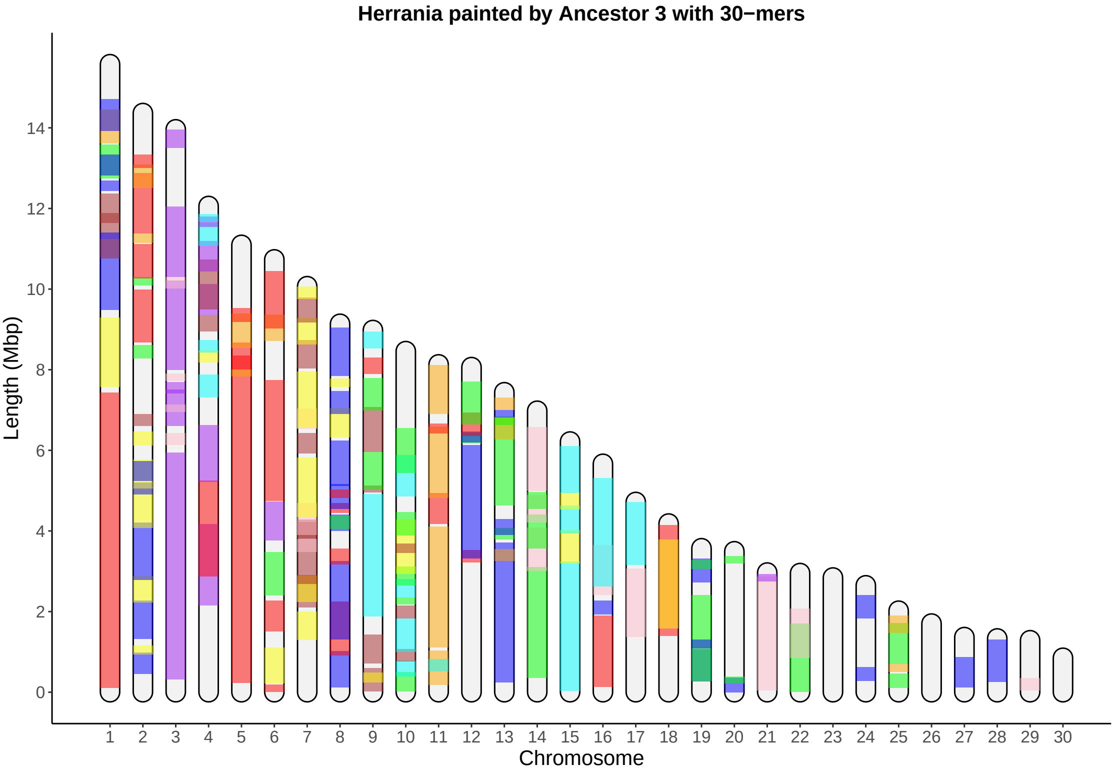
Herrania painted by Ancestor 3 with 30−mers
Length (Mbp)
0
2
4
6
8
10
12
14
1
2
3
4
5
6
7
8
9
10
11
12
13
14
15
16
17
18
19
20
21
22
23
24
25
26
27
28
29
30
Chromosome

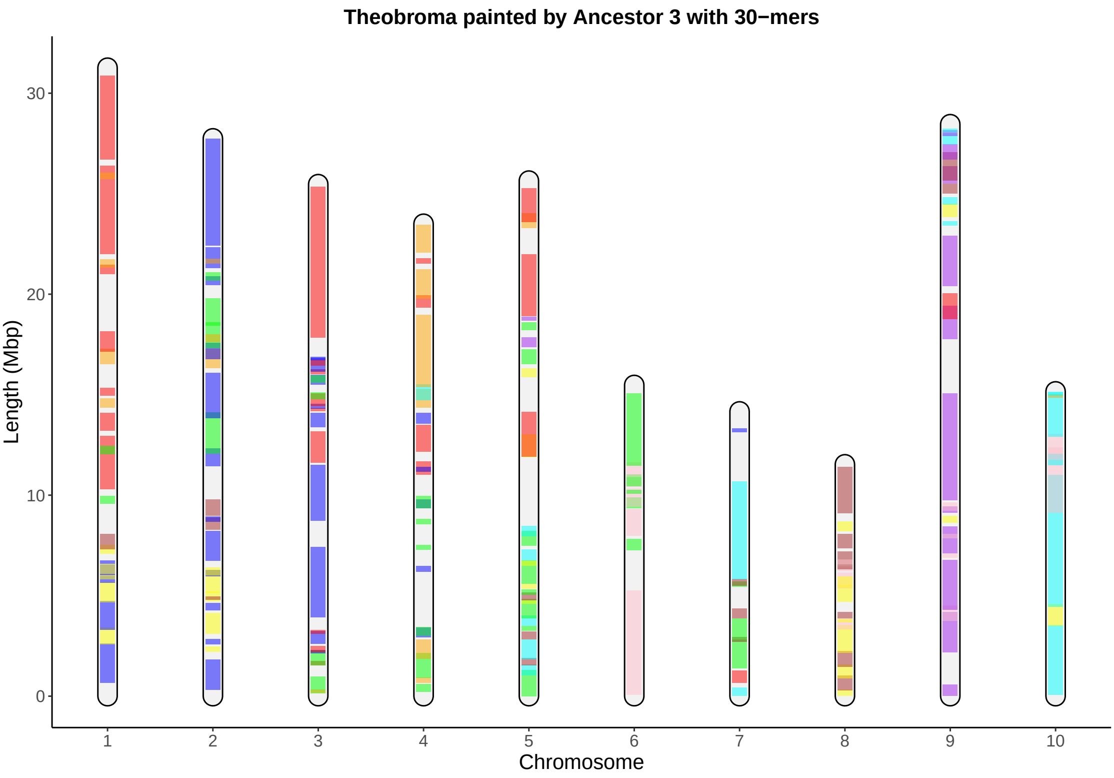
Theobroma painted by Ancestor 3 with 30−mers
Length (Mbp)
0
10
20
30
1
2
3
4
5
6
7
8
9
10
Chromosome

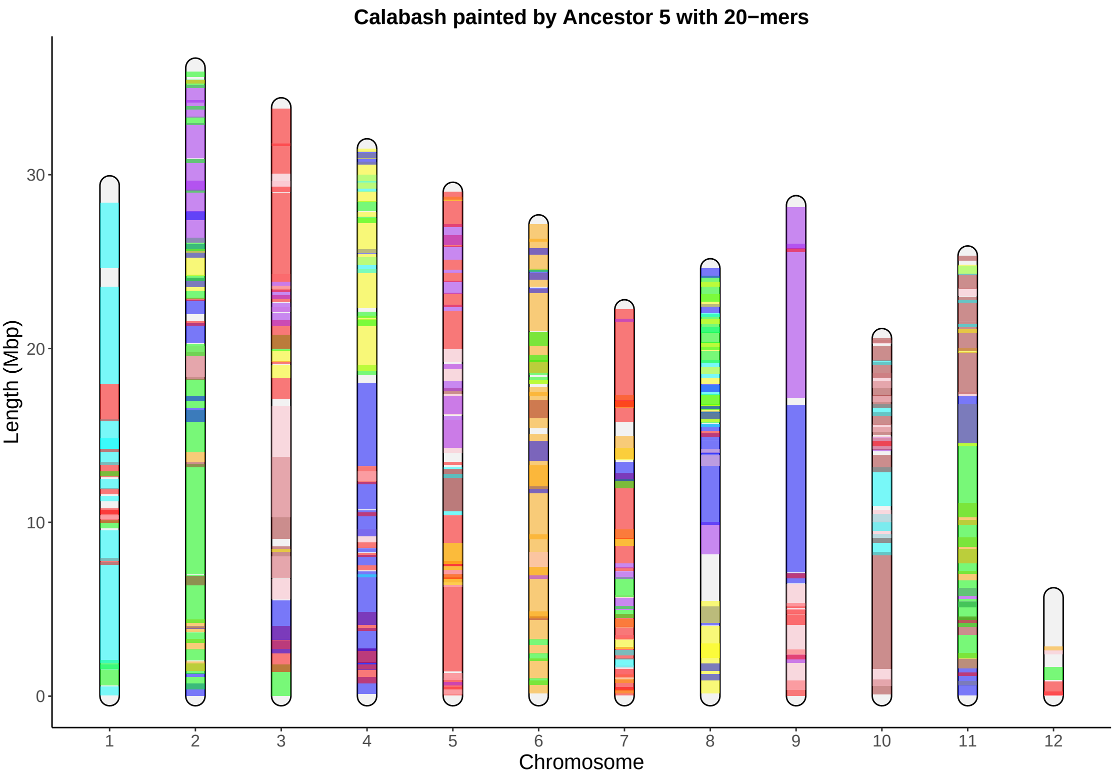
Calabash painted by Ancestor 5 with 20−mers
Length (Mbp)
30
20
10
0
1
2
3
4
5
6
7
8
9
10
11
12
Chromosome

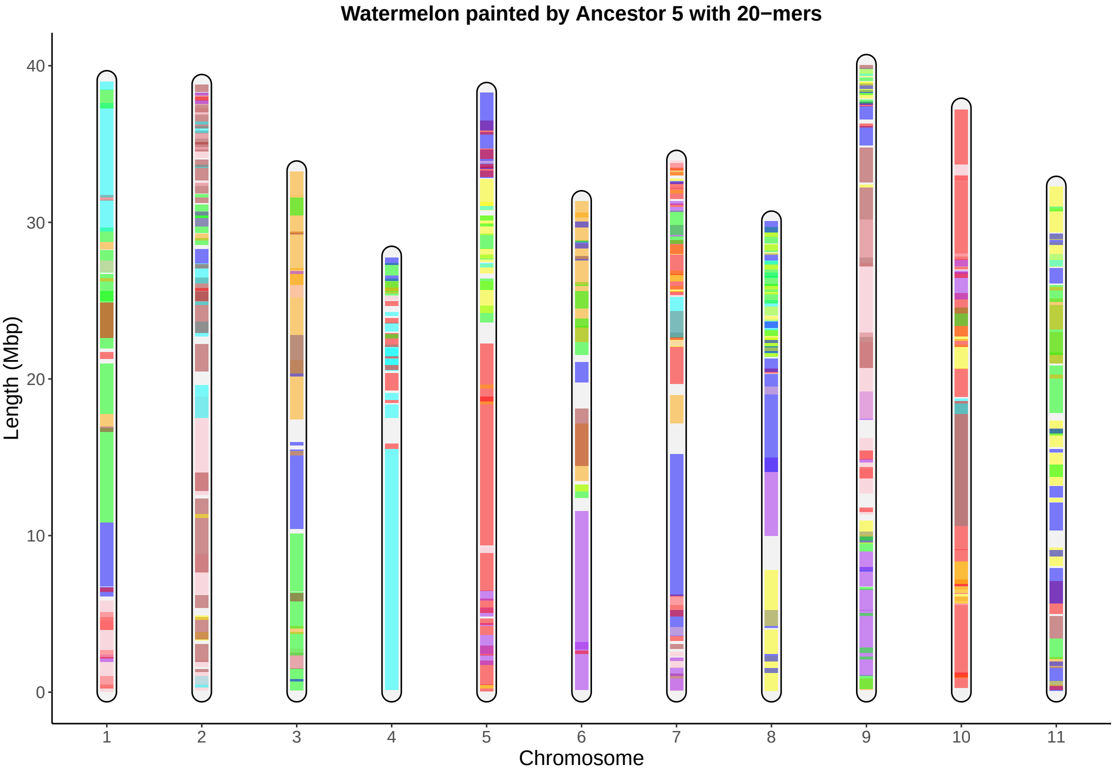

Watermelon painted by Ancestor 5 with 20−mers
Length (Mbp)
40
30
20
10
0
1
2
3
4
5
6
7
8
9
10
11
Chromosome

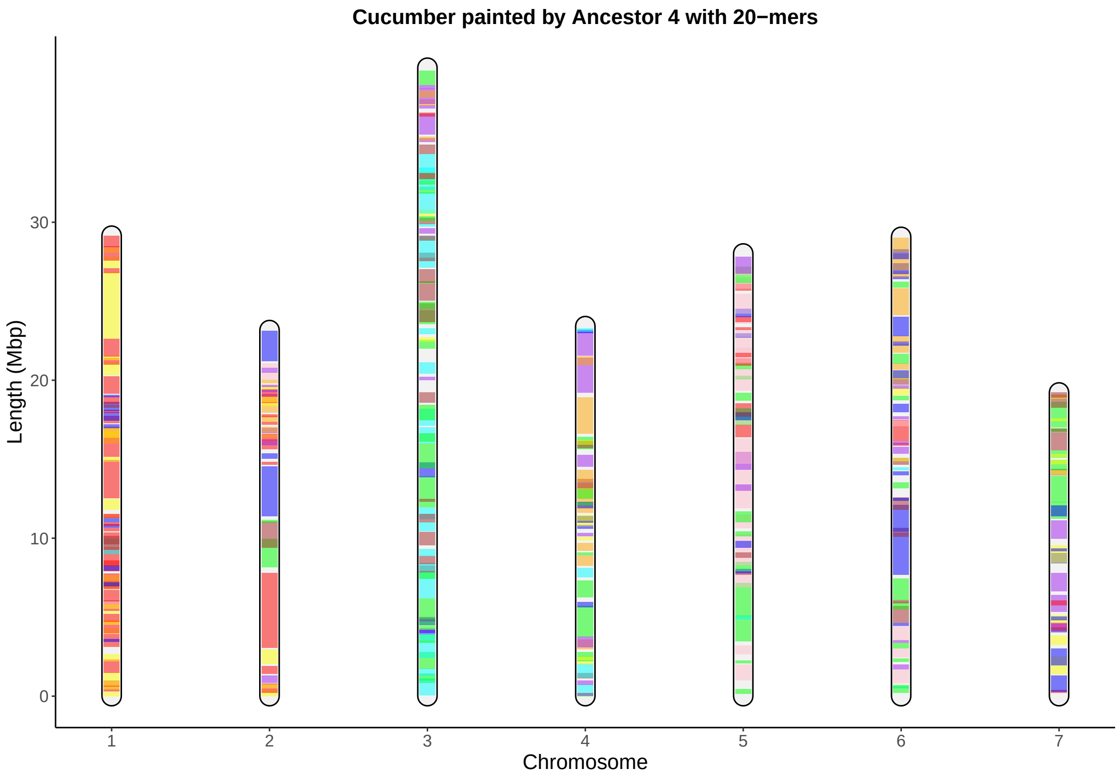
Cucumber painted by Ancestor 4 with 20−mers
Length (Mbp)
0
10
20
30
Chromosome
1
2
3
4
5
6
7

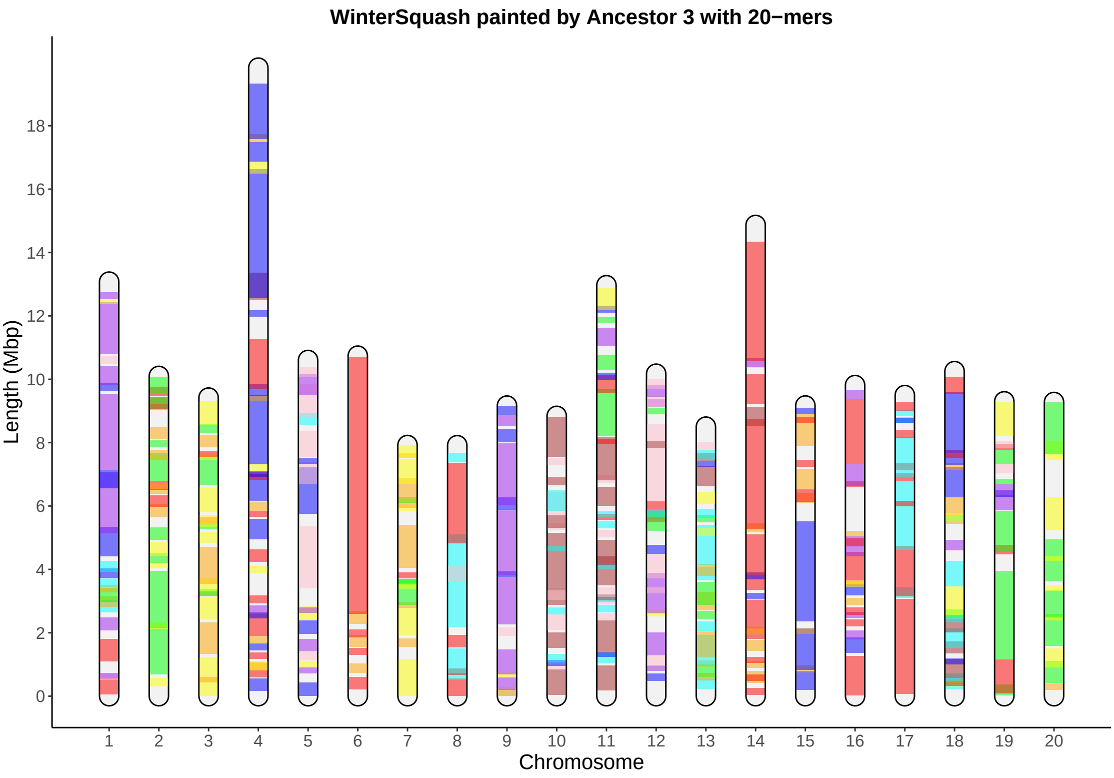

WinterSquash painted by Ancestor 3 with 20−mers
Length (Mbp)
0
2
4
6
8
10
12
14
16
18
1 2 3 4 5 6 7 8 9 10 11 12 13 14 15 16 17 18 19 20
Chromosome

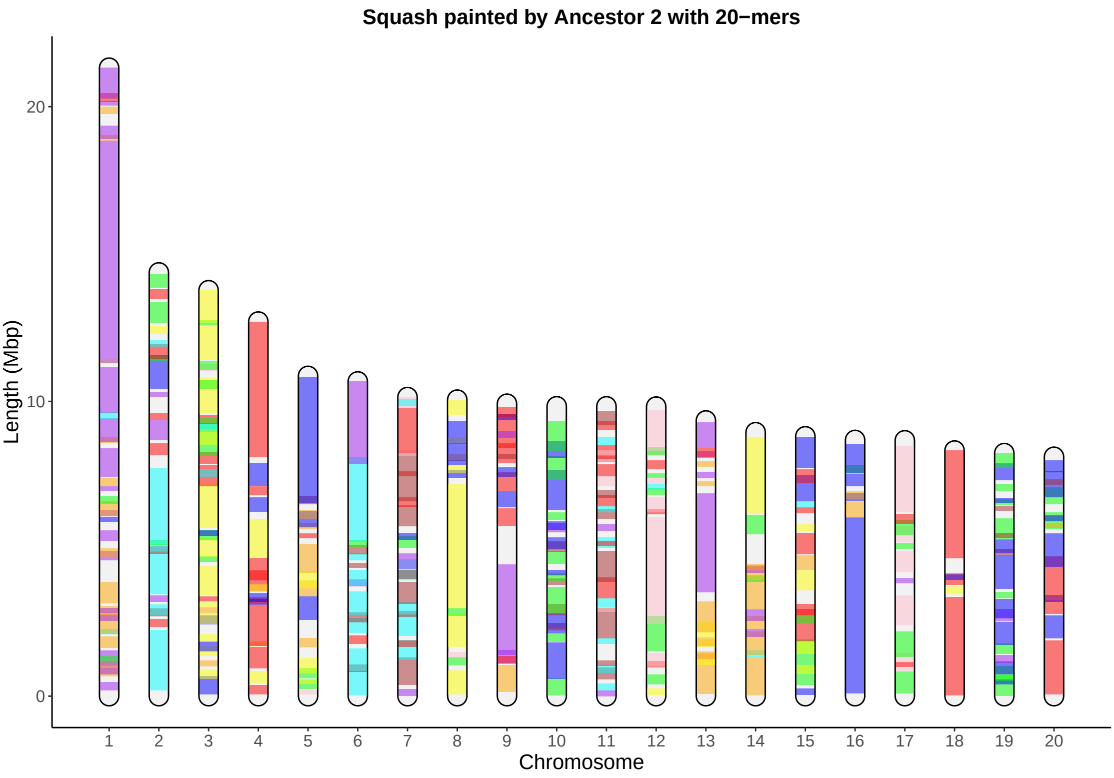

Squash painted by Ancestor 2 with 20−mers
Length (Mbp)
20
10
0
1
2
3
4
5
6
7
8
9
10
11
12
13
14
15
16
17
18
19
20
Chromosome

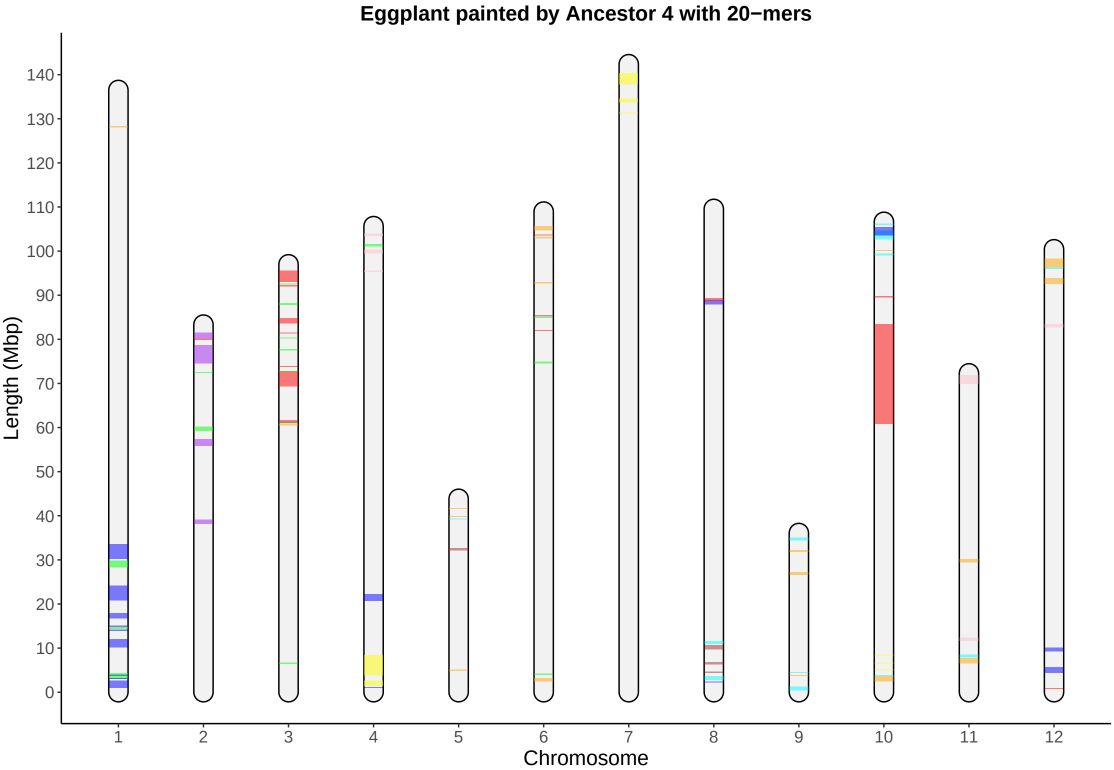
Eggplant painted by Ancestor 4 with 20−mers
Length (Mbp)
0
10
20
30
40
50
60
70
80
90
100
110
120
130
140
1
2
3
4
5
6
7
8
9
10
11
12
Chromosome

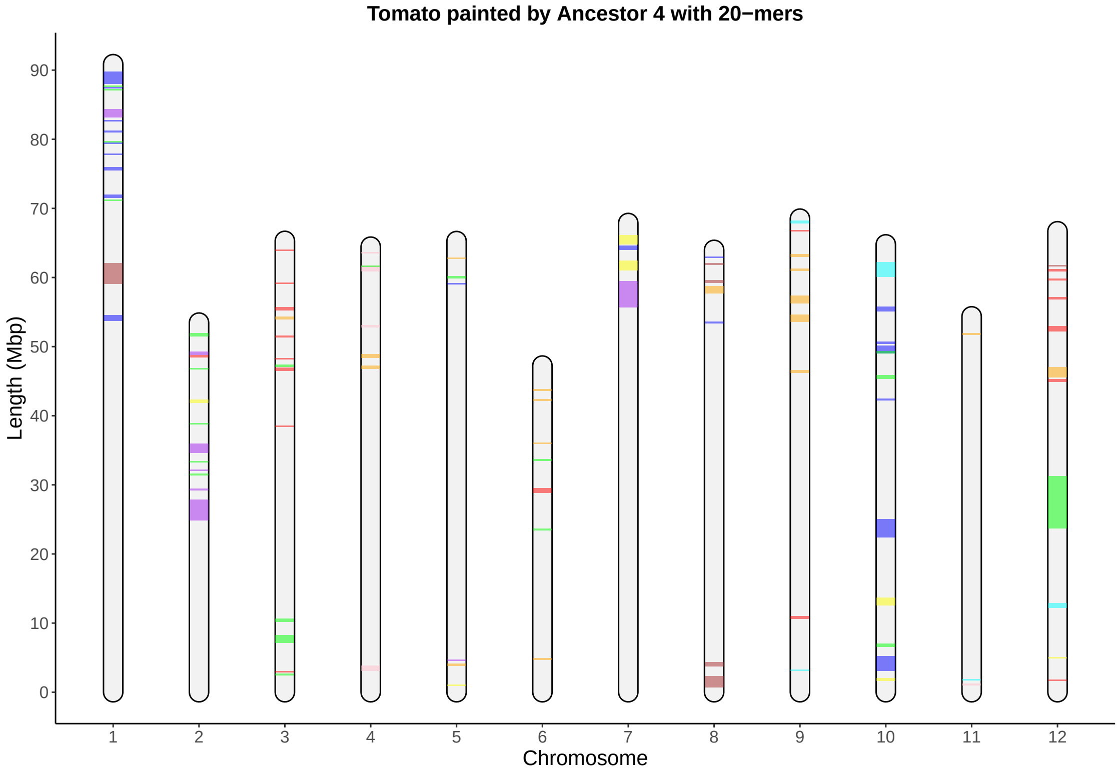
Tomato painted by Ancestor 4 with 20−mers
Length (Mbp)
0
10
20
30
40
50
60
70
80
90
1
2
3
4
5
6
7
8
9
10
11
12
Chromosome

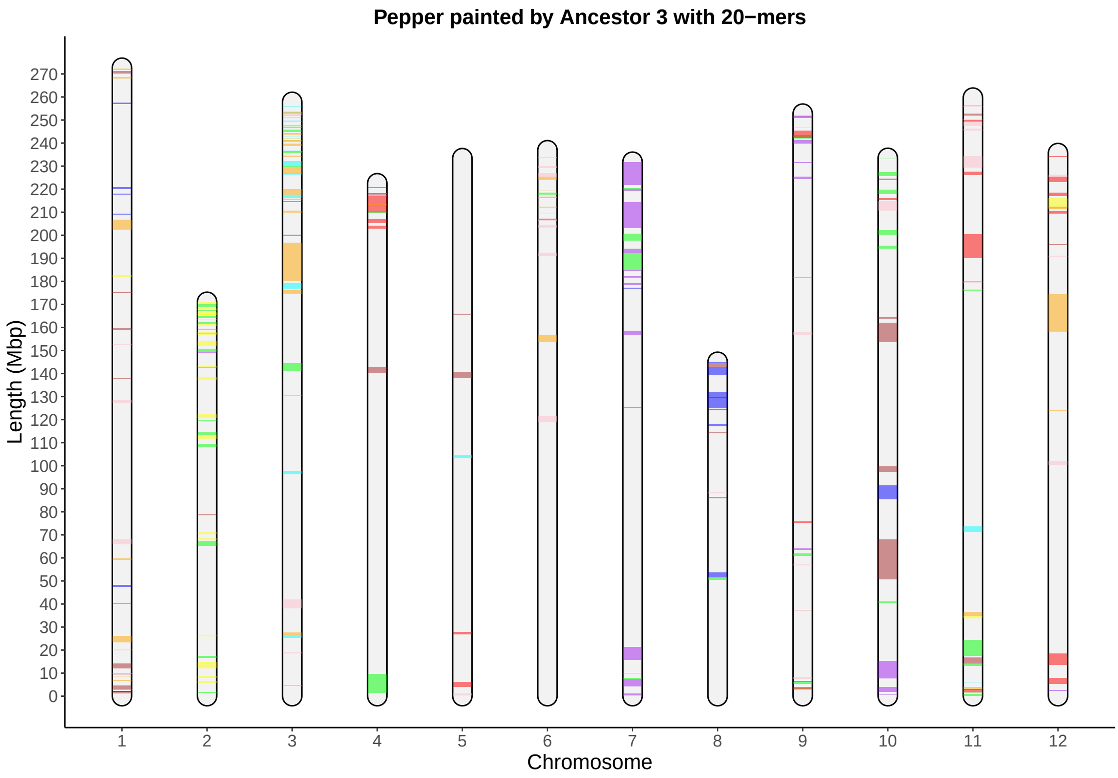
Pepper painted by Ancestor 3 with 20−mers
Length (Mbp)
270
260
250
240
230
220
210
200
190
180
170
160
150
140
130
120
110
100
90
80
70
60
50
40
30
20
10
0
1
2
3
4
5
6
7
8
9
10
11
12
Chromosome

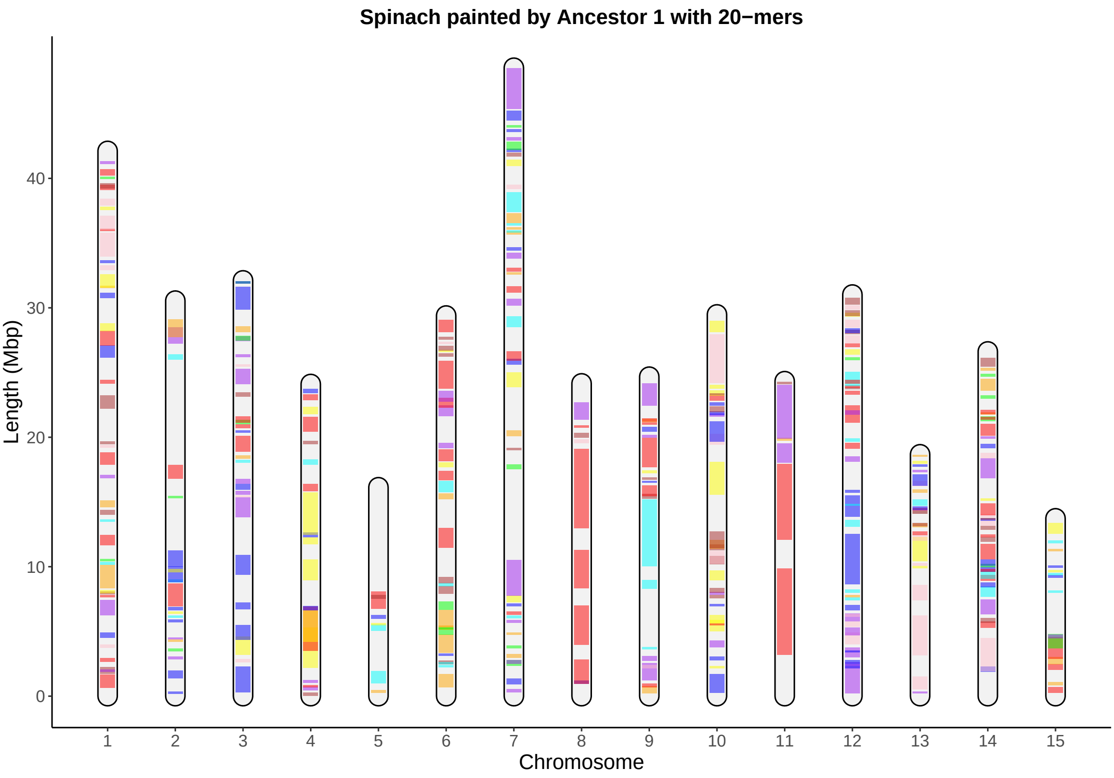
Spinach painted by Ancestor 1 with 20−mers
Length (Mbp)
0
10
20
30
40
Chromosome
1
2
3
4
5
6
7
8
9
10
11
12
13
14
15

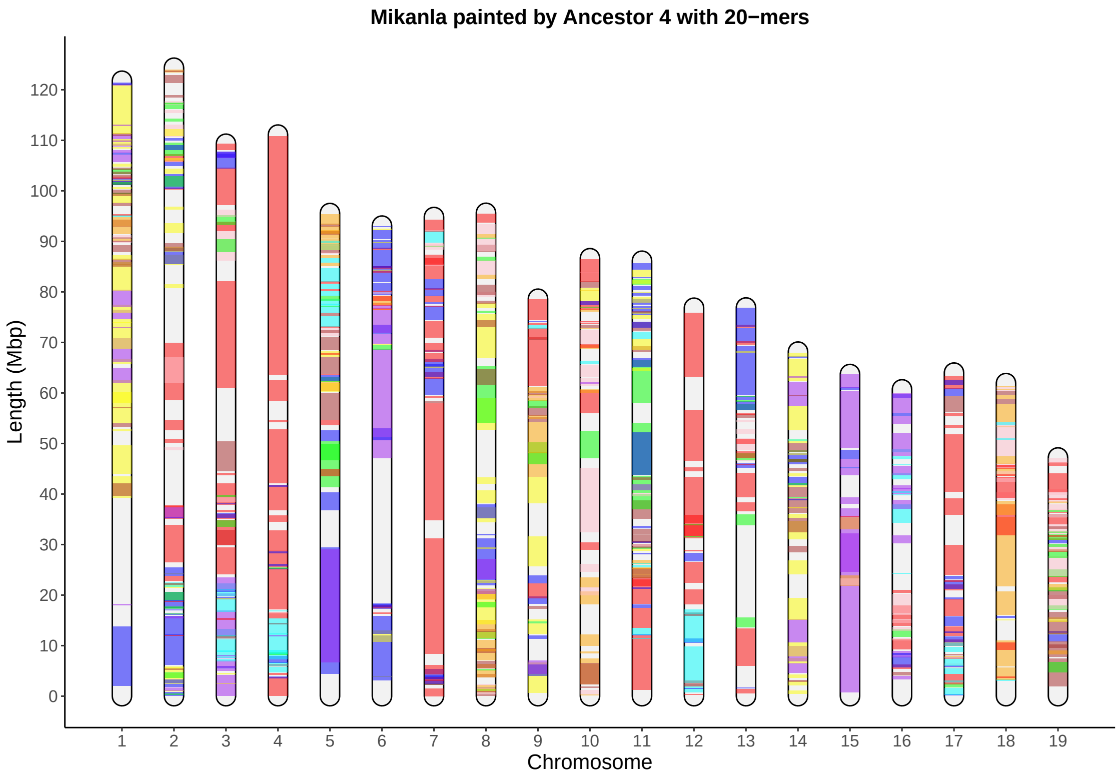
Mikanla painted by Ancestor 4 with 20−mers
Length (Mbp)
0
10
20
30
40
50
60
70
80
90
100
110
120
Chromosome
1
2
3
4
5
6
7
8
9
10
11
12
13
14
15
16
17
18
19

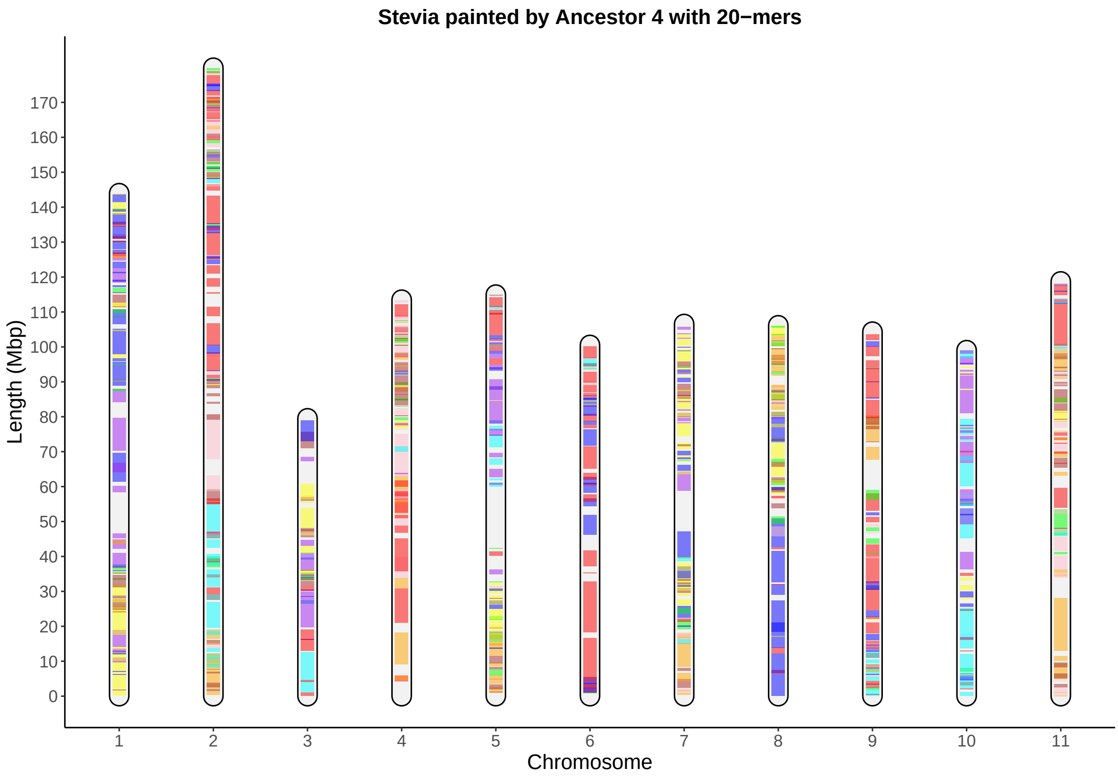
Stevia painted by Ancestor 4 with 20−mers
Length (Mbp)
0
10
20
30
40
50
60
70
80
90
100
110
120
130
140
150
160
170
Chromosome
1
2
3
4
5
6
7
8
9
10
11

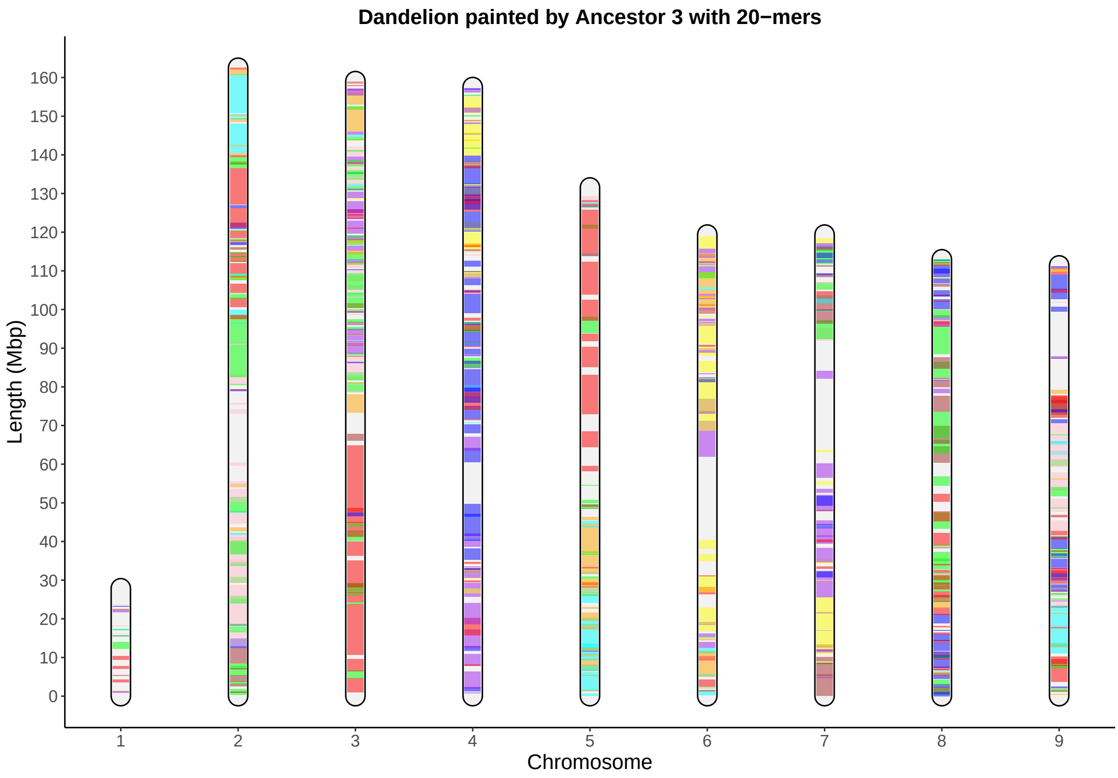

Dandelion painted by Ancestor 3 with 20−mers
Length (Mbp)
0
10
20
30
40
50
60
70
80
90
100
110
120
130
140
150
160
Chromosome
1
2
3
4
5
6
7
8
9

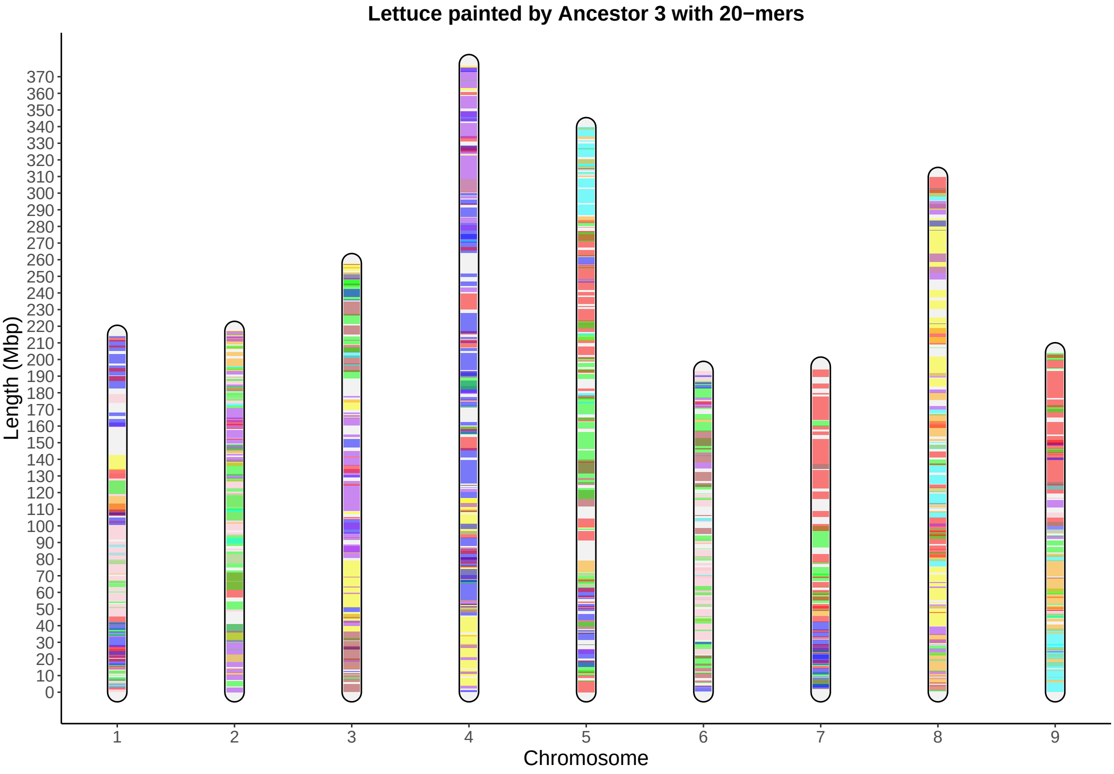

Lettuce painted by Ancestor 3 with 20−mers
Length (Mbp)
0
10
20
30
40
50
60
70
80
90
100
110
120
130
140
150
160
170
180
190
200
210
220
230
240
250
260
270
280
290
300
310
320
330
340
350
360
370
1
2
3
4
5
6
7
8
9
Chromosome

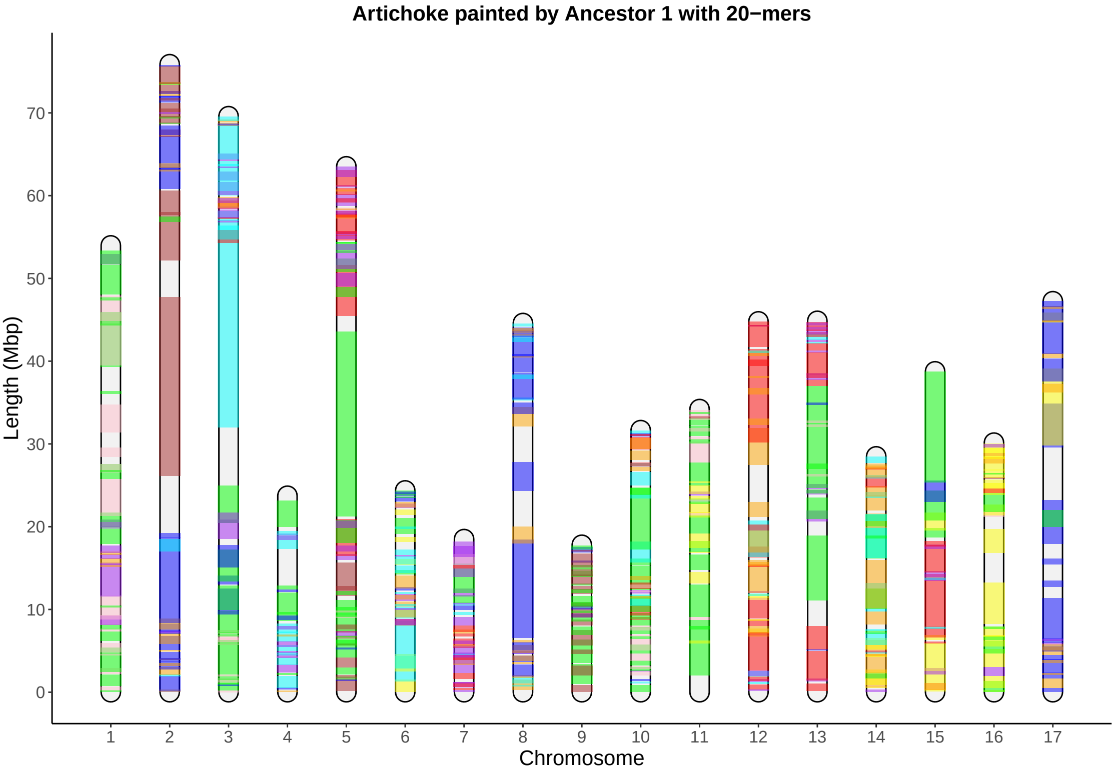

Artichoke painted by Ancestor 1 with 20−mers
Length (Mbp)
0
10
20
30
40
50
60
70
Chromosome
1
2
3
4
5
6
7
8
9
10
11
12
13
14
15
16
17

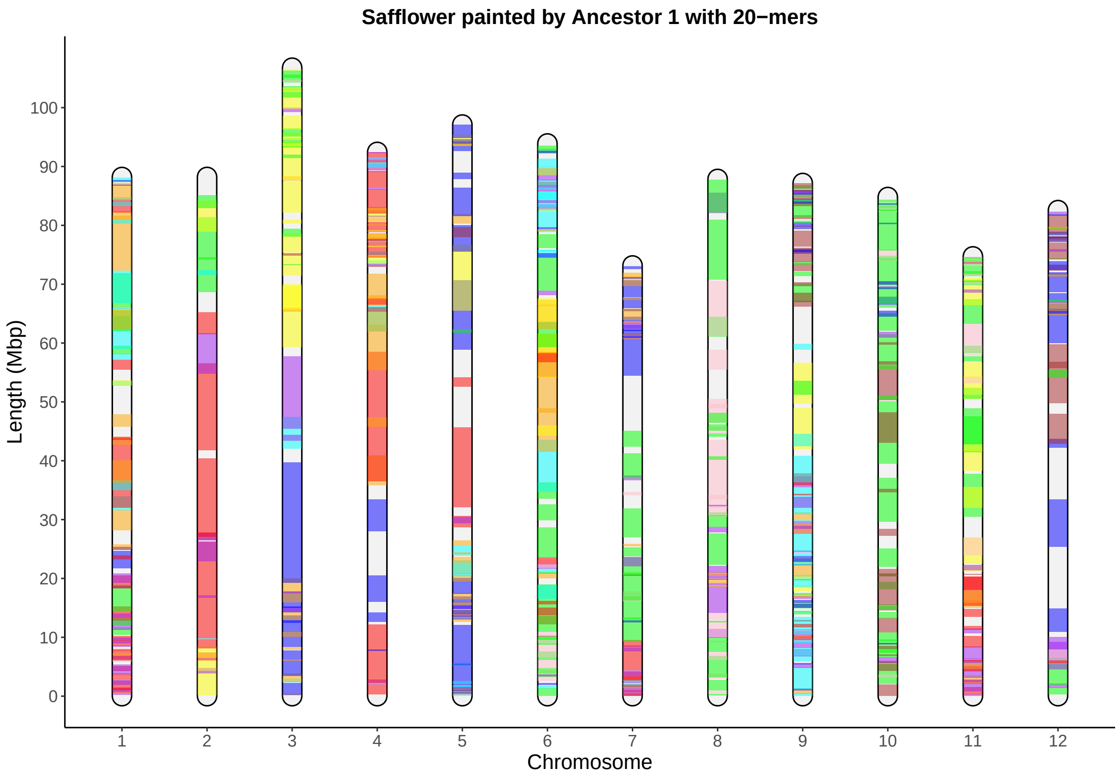

Safflower painted by Ancestor 1 with 20−mers
Length (Mbp)
0
10
20
30
40
50
60
70
80
90
100
Chromosome
1
2
3
4
5
6
7
8
9
10
11
12

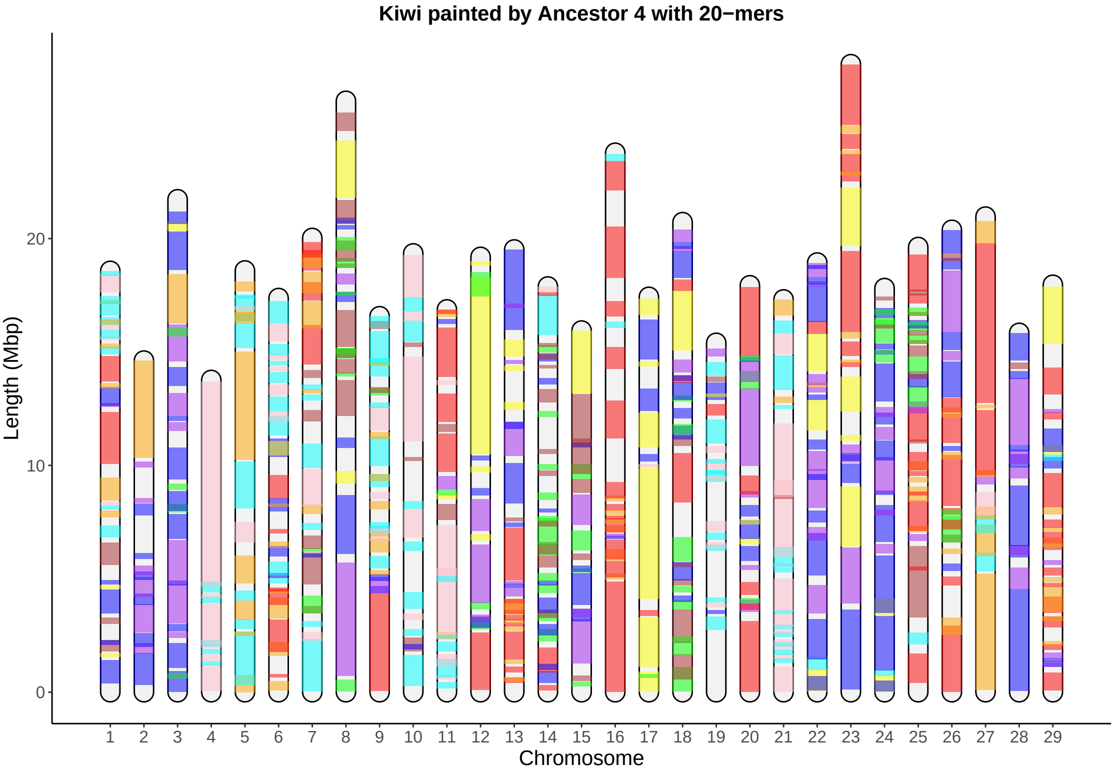

Kiwi painted by Ancestor 4 with 20−mers
Length (Mbp)
0
10
20
1
2
3
4
5
6
7
8
9
10
11
12
13
14
15
16
17
18
19
20
21
22
23
24
25
26
27
28
29
Chromosome

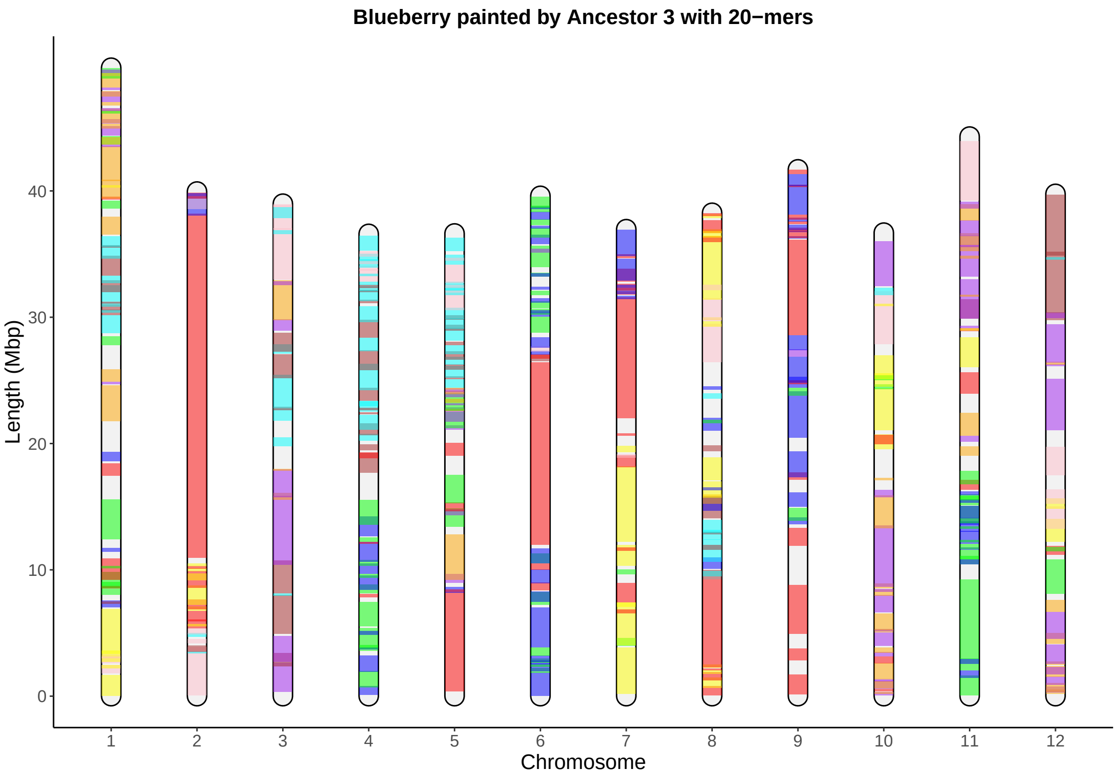
Blueberry painted by Ancestor 3 with 20−mers
Length (Mbp)
0
10
20
30
40
Chromosome
1
2
3
4
5
6
7
8
9
10
11
12

Persimon painted by Ancestor 1 with 20−mers

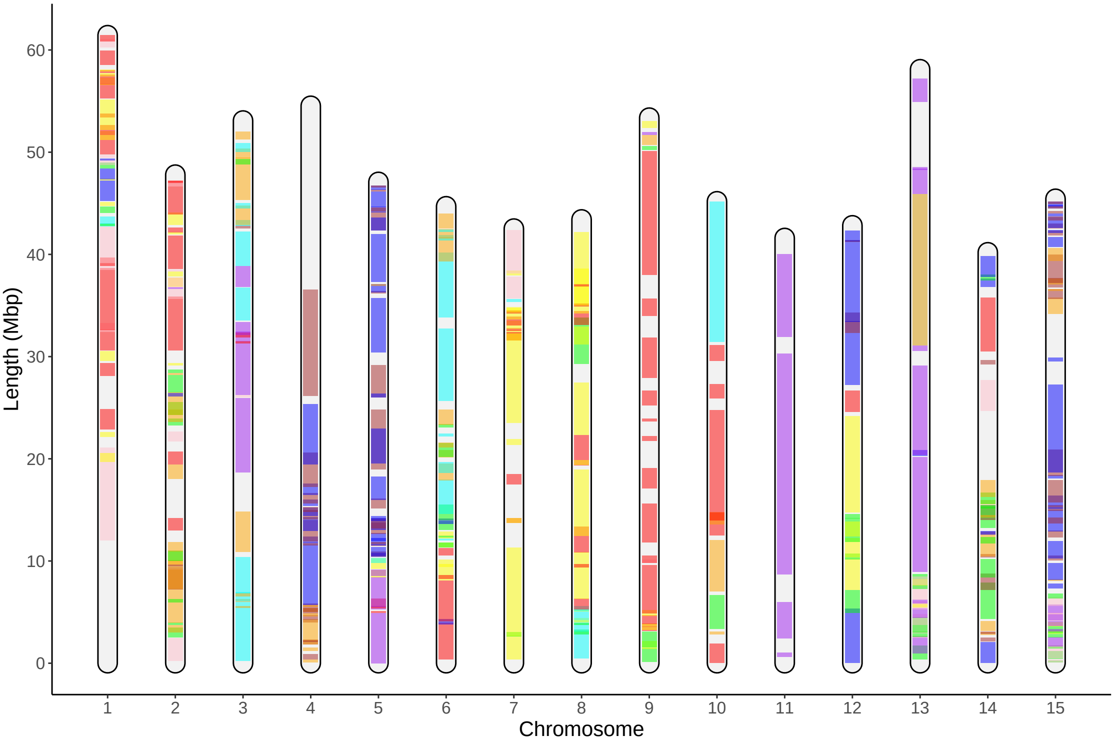

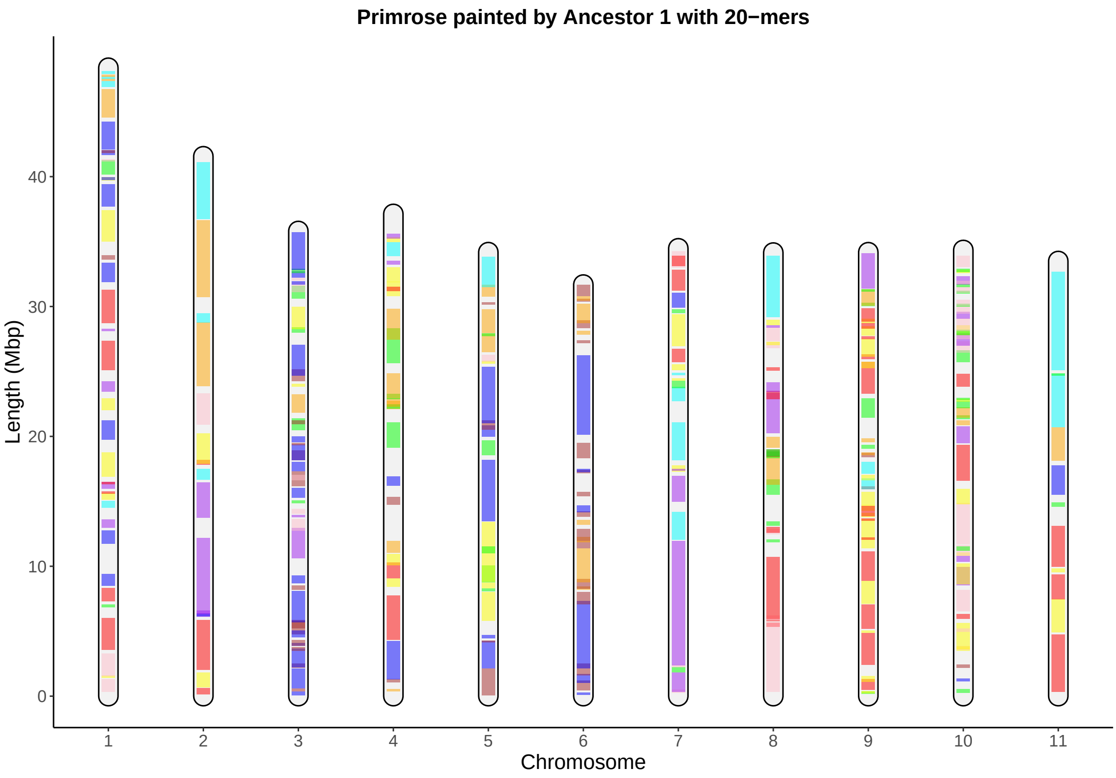

Primrose painted by Ancestor 1 with 20−mers
Length (Mbp)
0
10
20
30
40
1
2
3
4
5
6
7
8
9
10
11
Chromosome

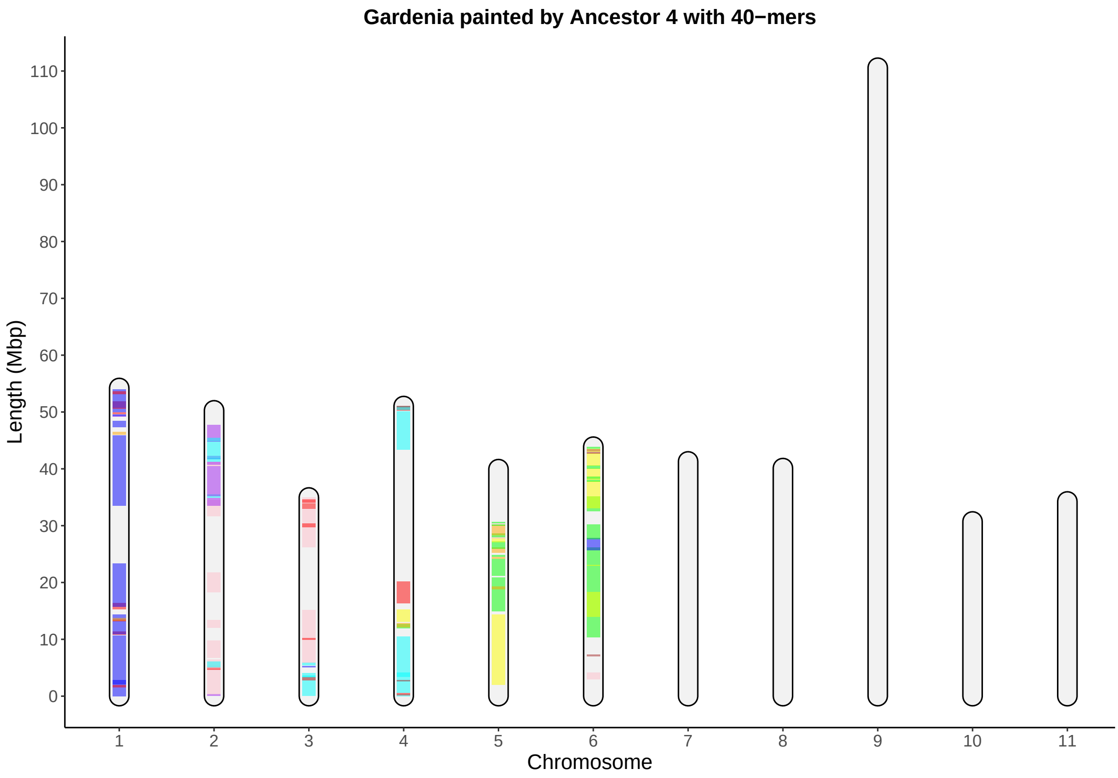

Gardenia painted by Ancestor 4 with 40−mers
Length (Mbp)
0
10
20
30
40
50
60
70
80
90
100
110
Chromosome
1
2
3
4
5
6
7
8
9
10
11

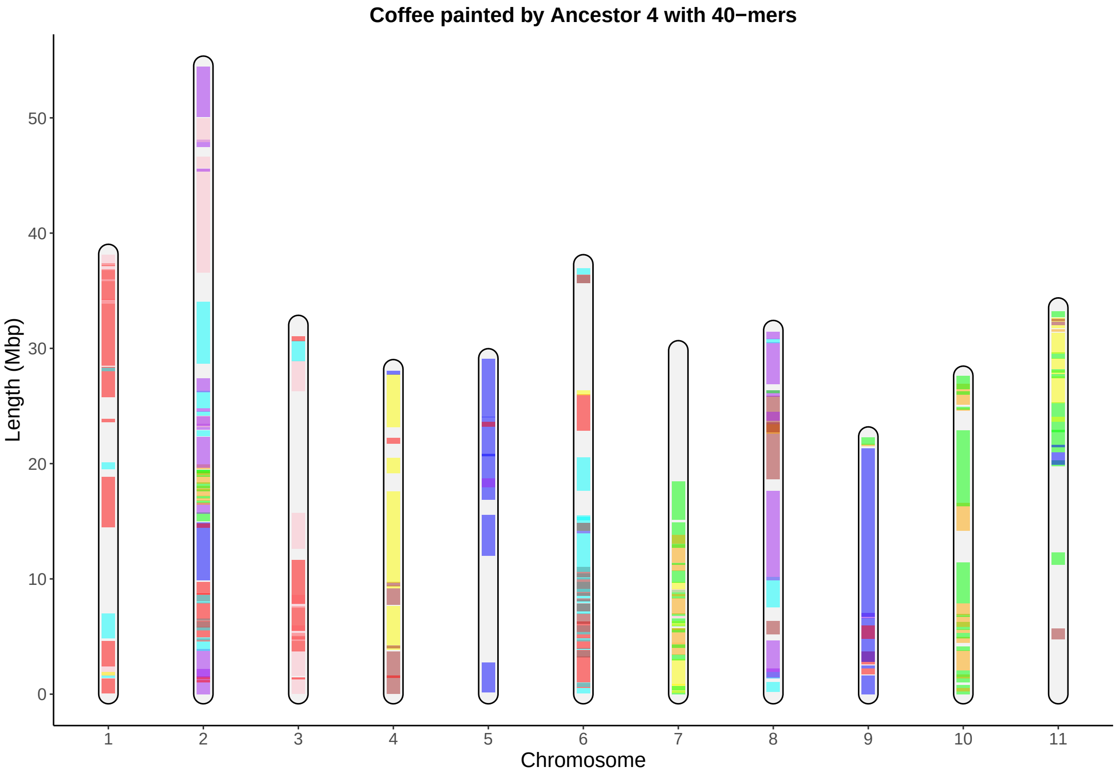
Coffee painted by Ancestor 4 with 40−mers
Length (Mbp)
0
10
20
30
40
50
1
2
3
4
5
6
7
8
9
10
11
Chromosome

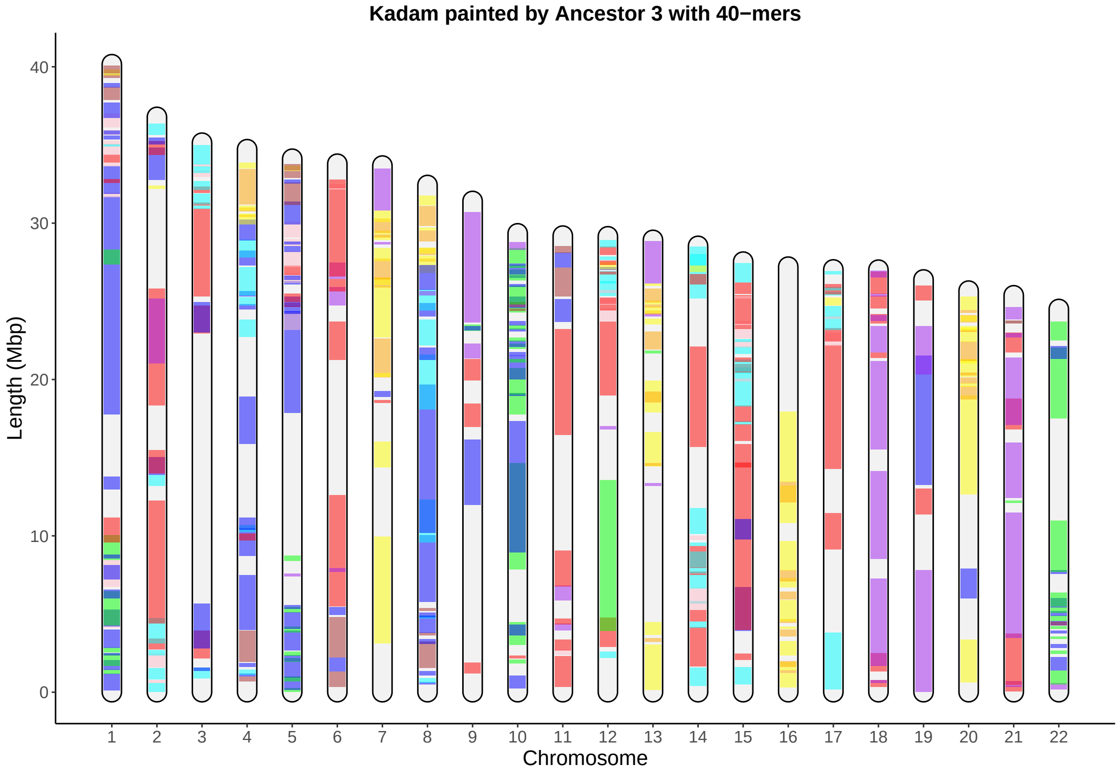
Kadam painted by Ancestor 3 with 40−mers
Length (Mbp)
0
10
20
30
40
1
2
3
4
5
6
7
8
9
10
11
12
13
14
15
16
17
18
19
20
21
22
Chromosome

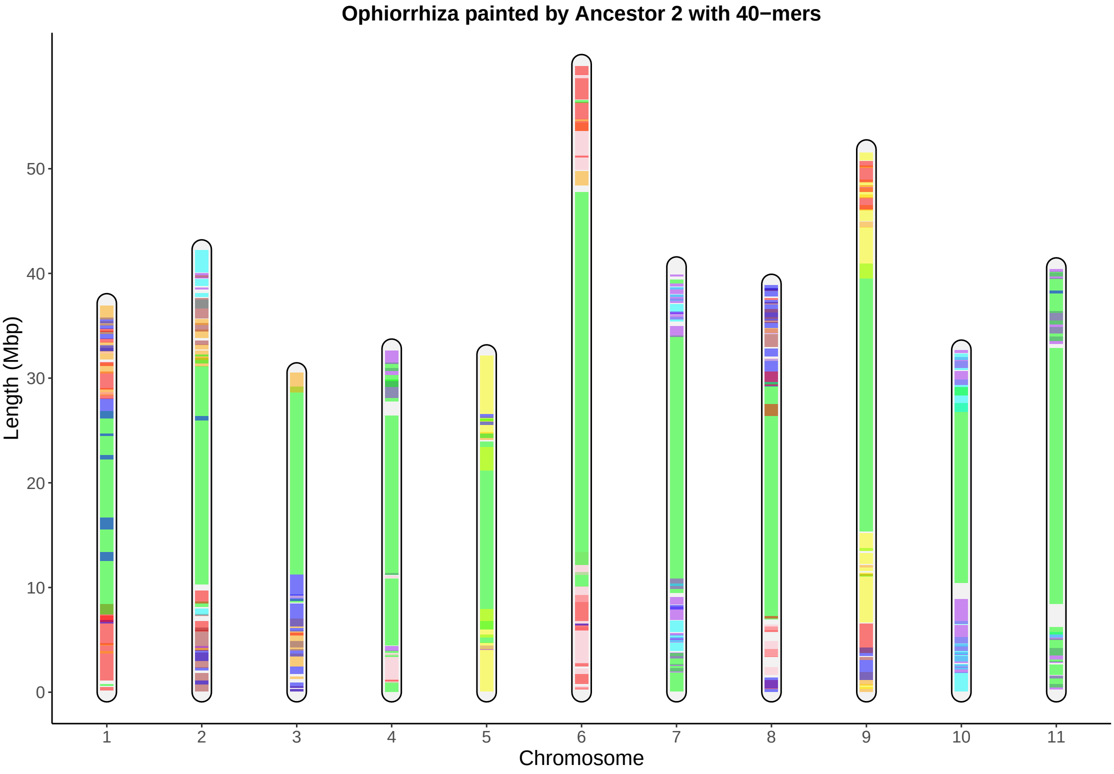

Ophiorrhiza painted by Ancestor 2 with 40−mers
Length (Mbp)
0
10
20
30
40
50
1
2
3
4
5
6
7
8
9
10
11
Chromosome

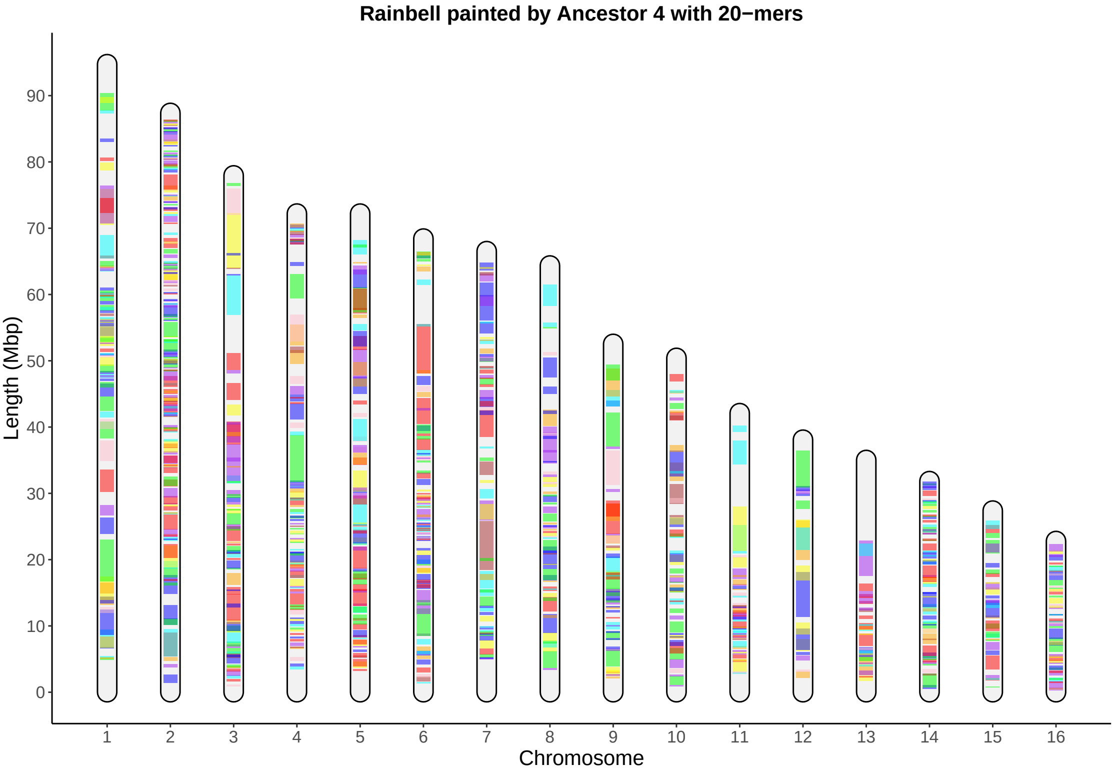

Rainbell painted by Ancestor 4 with 20−mers
Length (Mbp)
0
10
20
30
40
50
60
70
80
90
Chromosome
1
2
3
4
5
6
7
8
9
10
11
12
13
14
15
16

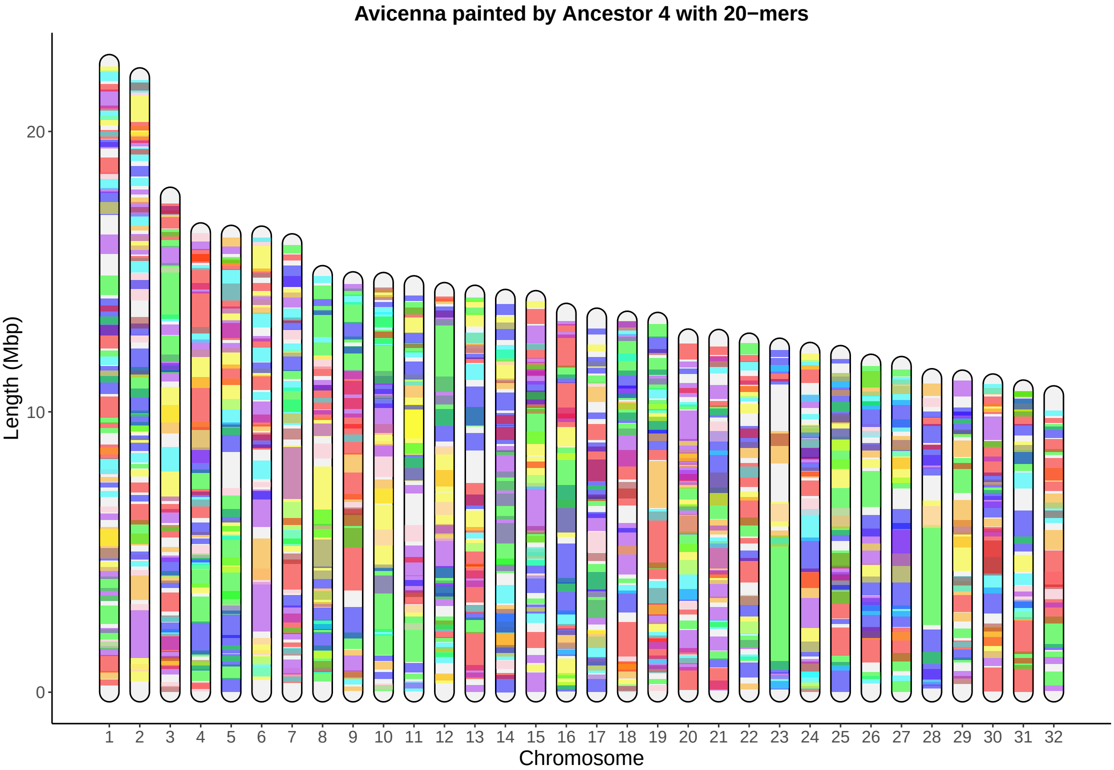

Avicenna painted by Ancestor 4 with 20−mers
Length (Mbp)
20
10
0
1
2
3
4
5
6
7
8
9
10
11
12
13
14
15
16
17
18
19
20
21
22
23
24
25
26
27
28
29
30
31
32
Chromosome

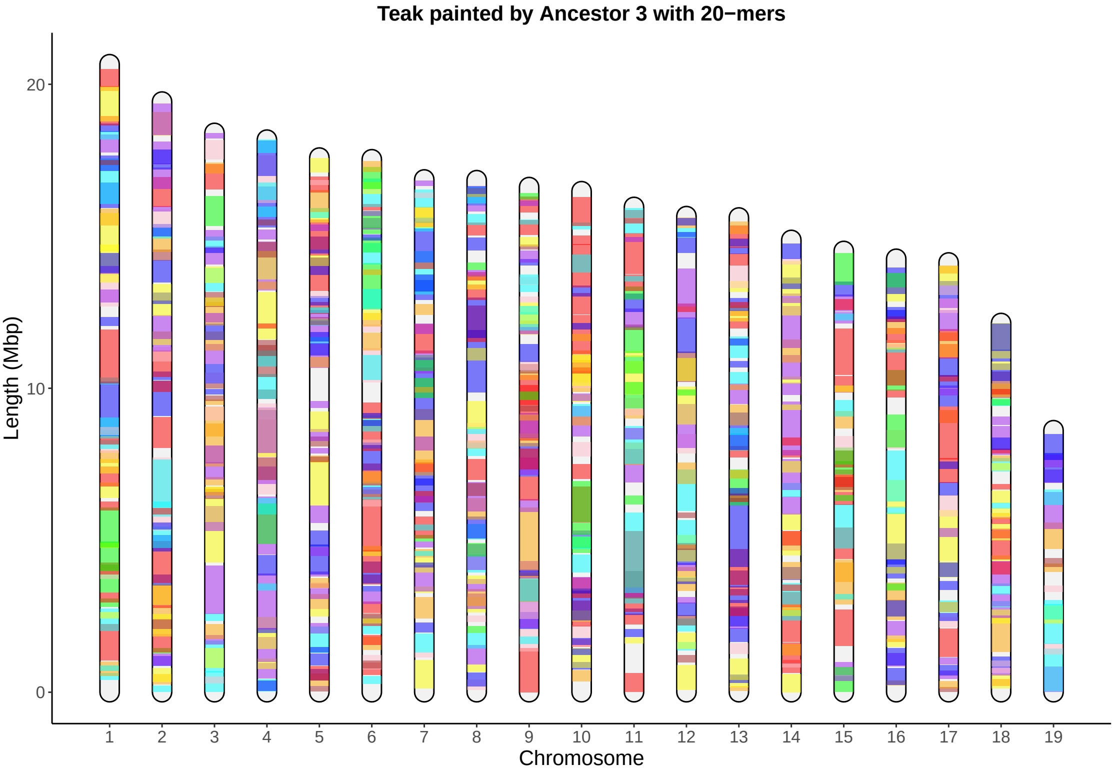
Teak painted by Ancestor 3 with 20−mers
Length (Mbp)
20
10
0
1
2
3
4
5
6
7
8
9
10
11
12
13
14
15
16
17
18
19
Chromosome

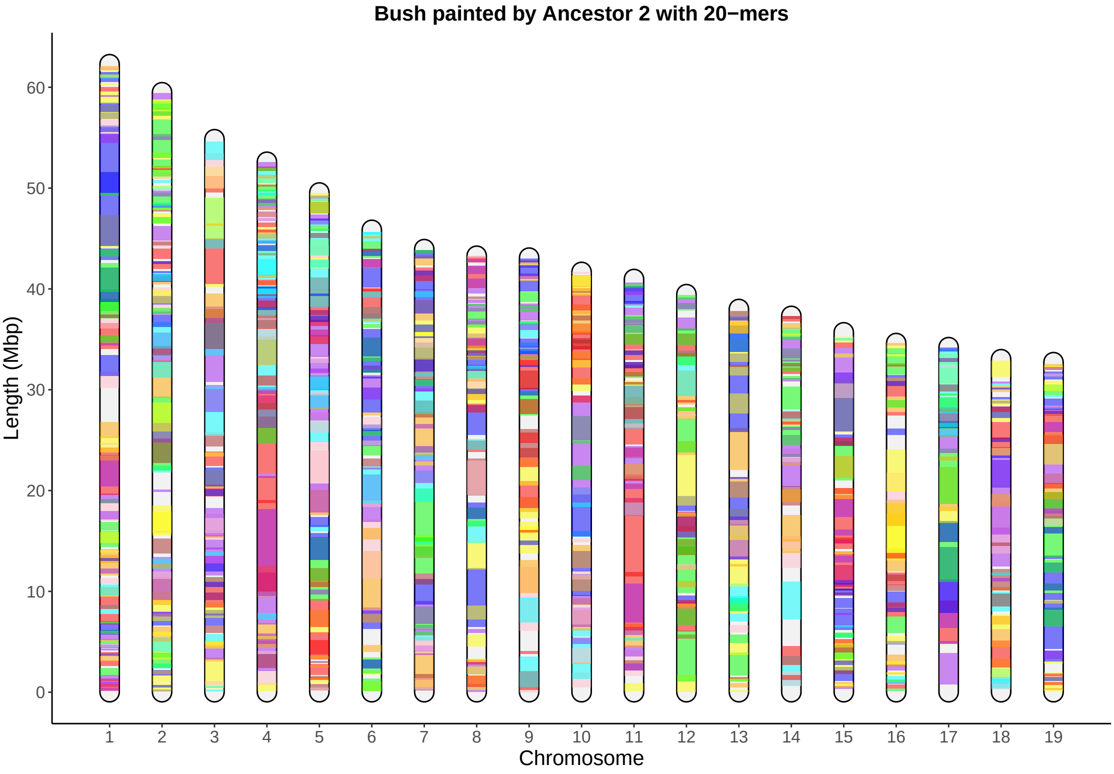

Bush painted by Ancestor 2 with 20−mers
Length (Mbp)
0
10
20
30
40
50
60
Chromosome
1
2
3
4
5
6
7
8
9
10
11
12
13
14
15
16
17
18
19

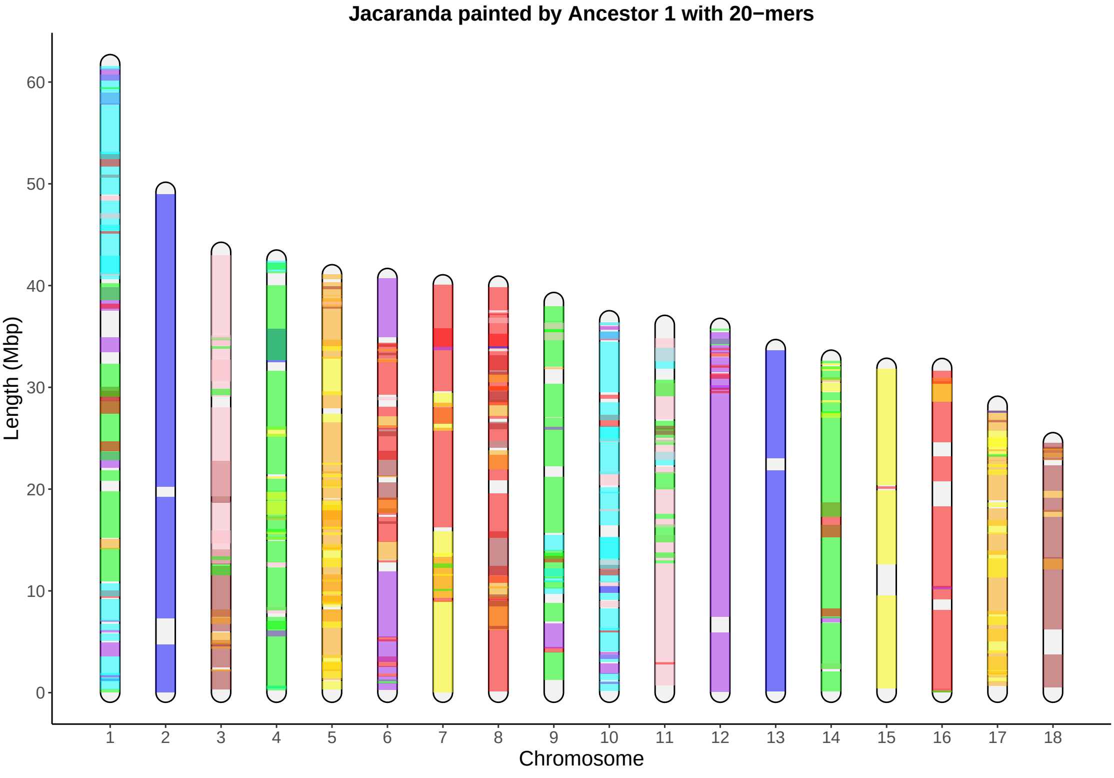
Jacaranda painted by Ancestor 1 with 20−mers
Length (Mbp)
0
10
20
30
40
50
60
Chromosome
1
2
3
4
5
6
7
8
9
10
11
12
13
14
15
16
17
18

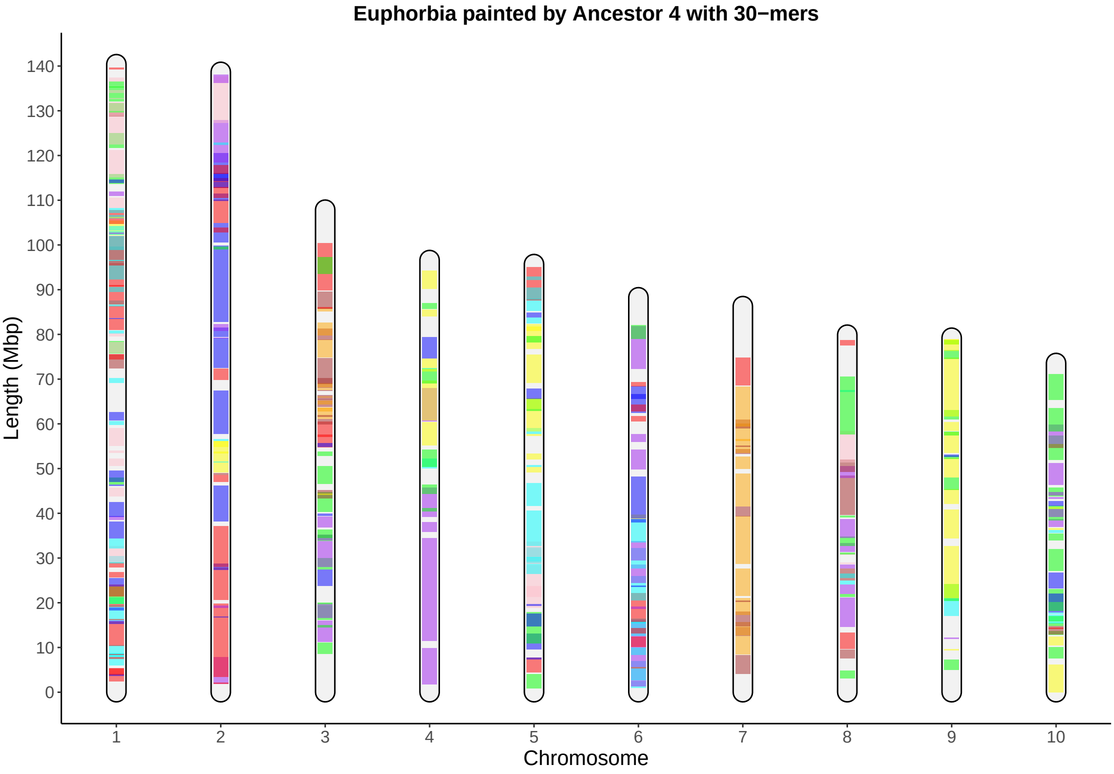

Euphorbia painted by Ancestor 4 with 30−mers
Length (Mbp)
140
130
120
110
100
90
80
70
60
50
40
30
20
10
0
1
2
3
4
5
6
7
8
9
10
Chromosome

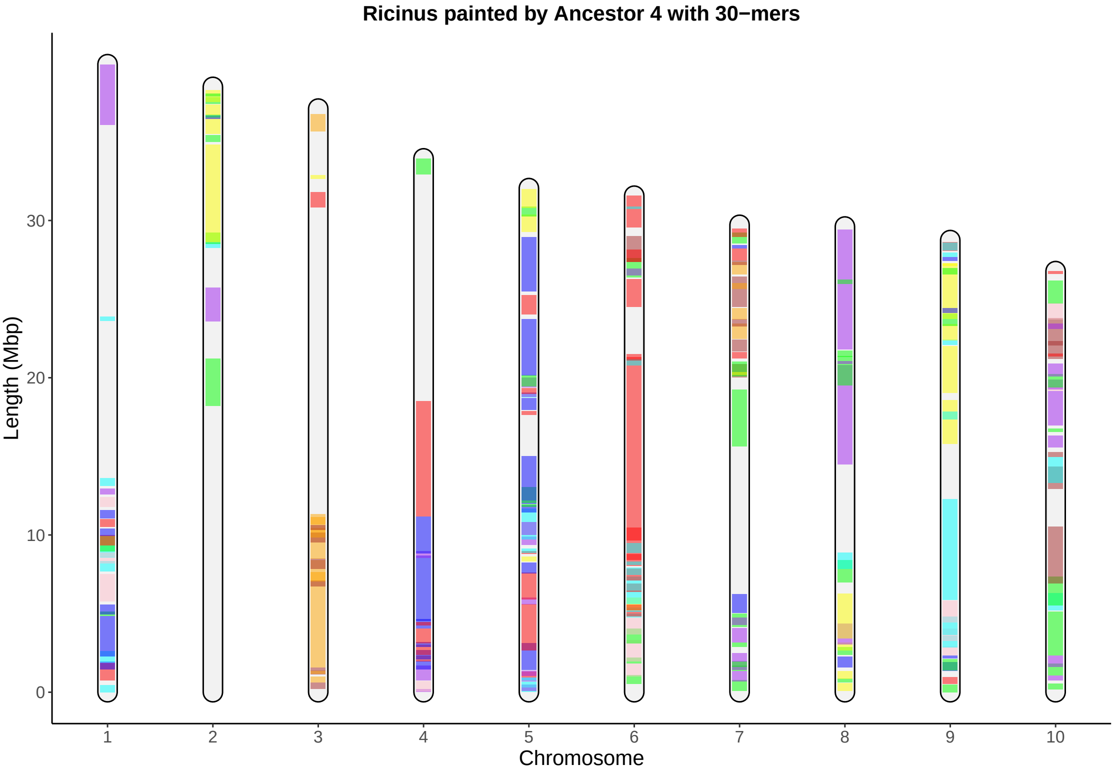

Ricinus painted by Ancestor 4 with 30−mers
Length (Mbp)
0
10
20
30
1
2
3
4
5
6
7
8
9
10
Chromosome

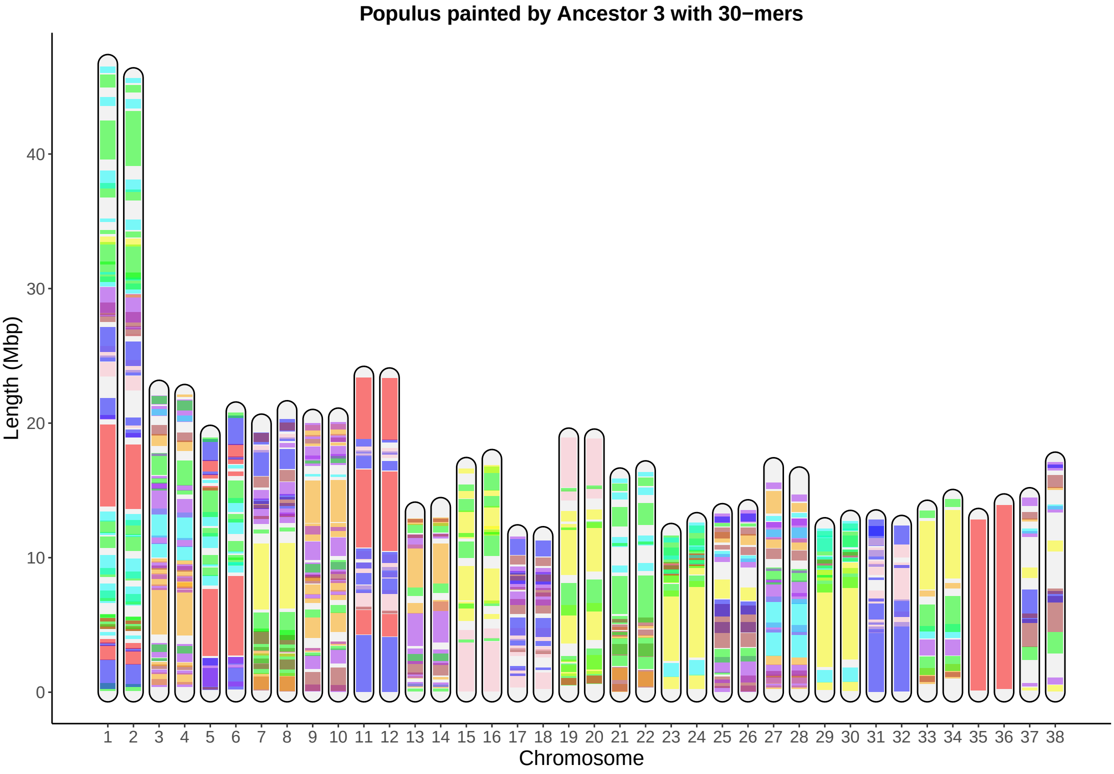

Populus painted by Ancestor 3 with 30−mers
Length (Mbp)
0
10
20
30
40
1 2 3 4 5 6 7 8 9 10 11 12 13 14 15 16 17 18 19 20 21 22 23 24 25 26 27 28 29 30 31 32 33 34 35 36 37 38
Chromosome

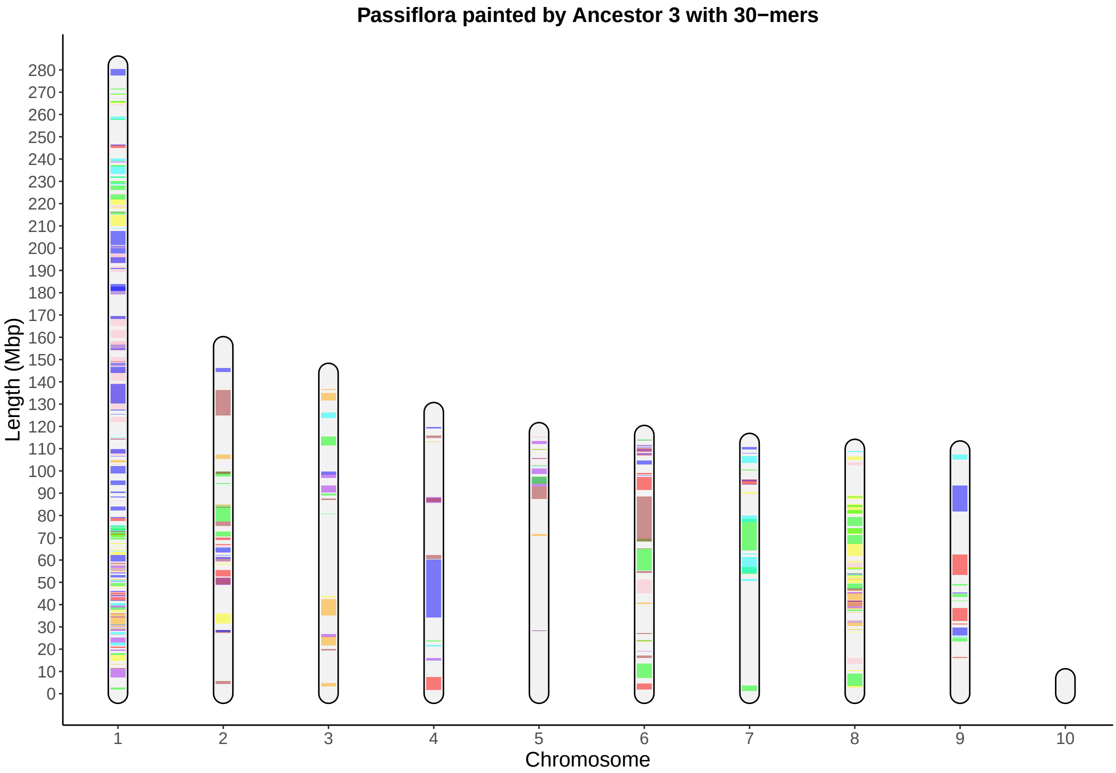

Passiflora painted by Ancestor 3 with 30−mers
Length (Mbp)
280
270
260
250
240
230
220
210
200
190
180
170
160
150
140
130
120
110
100
90
80
70
60
50
40
30
20
10
0
1
2
3
4
5
6
7
8
9
10
Chromosome

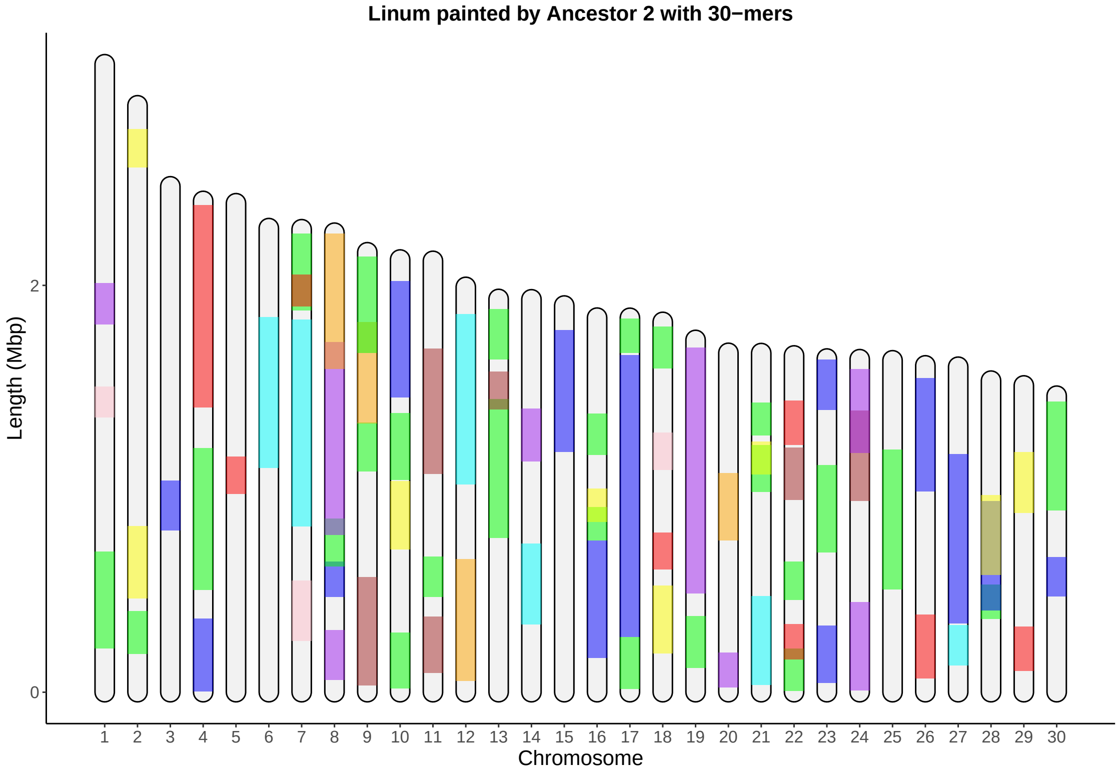

Linum painted by Ancestor 2 with 30−mers
Length (Mbp)
2
0
1
2
3
4
5
6
7
8
9
10
11
12
13
14
15
16
17
18
19
20
21
22
23
24
25
26
27
28
29
30
Chromosome

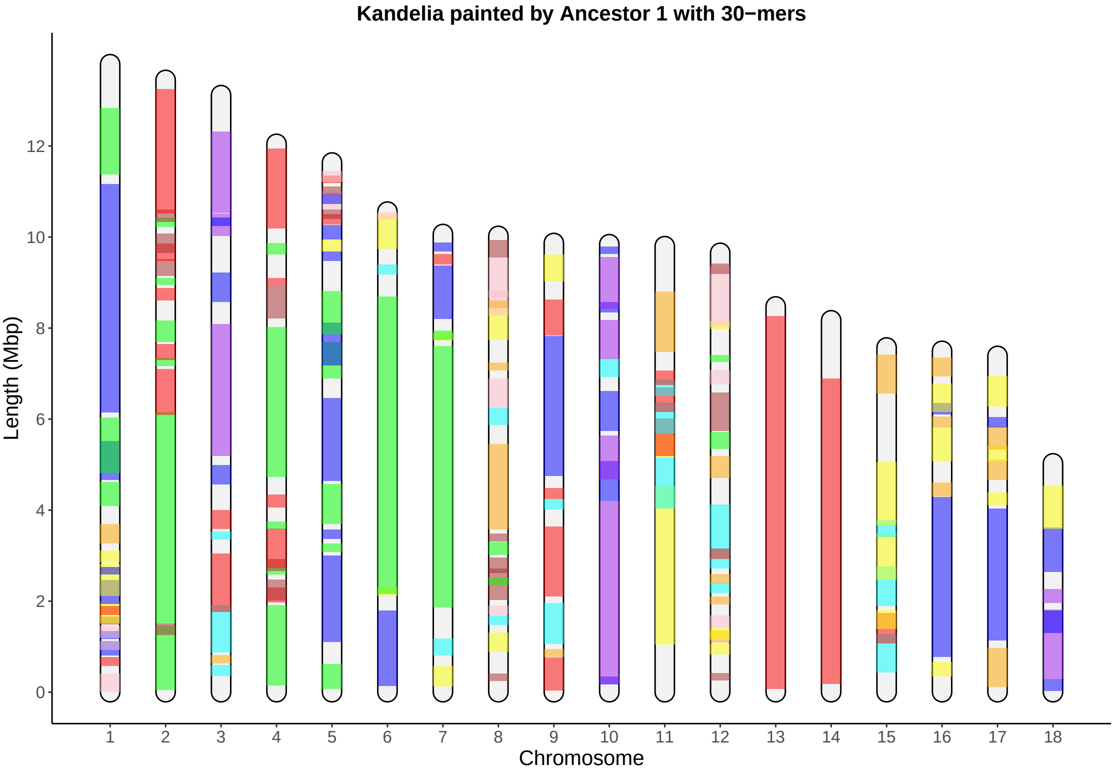
Kandelia painted by Ancestor 1 with 30−mers
Length (Mbp)
0
2
4
6
8
10
12
Chromosome
1
2
3
4
5
6
7
8
9
10
11
12
13
14
15
16
17
18

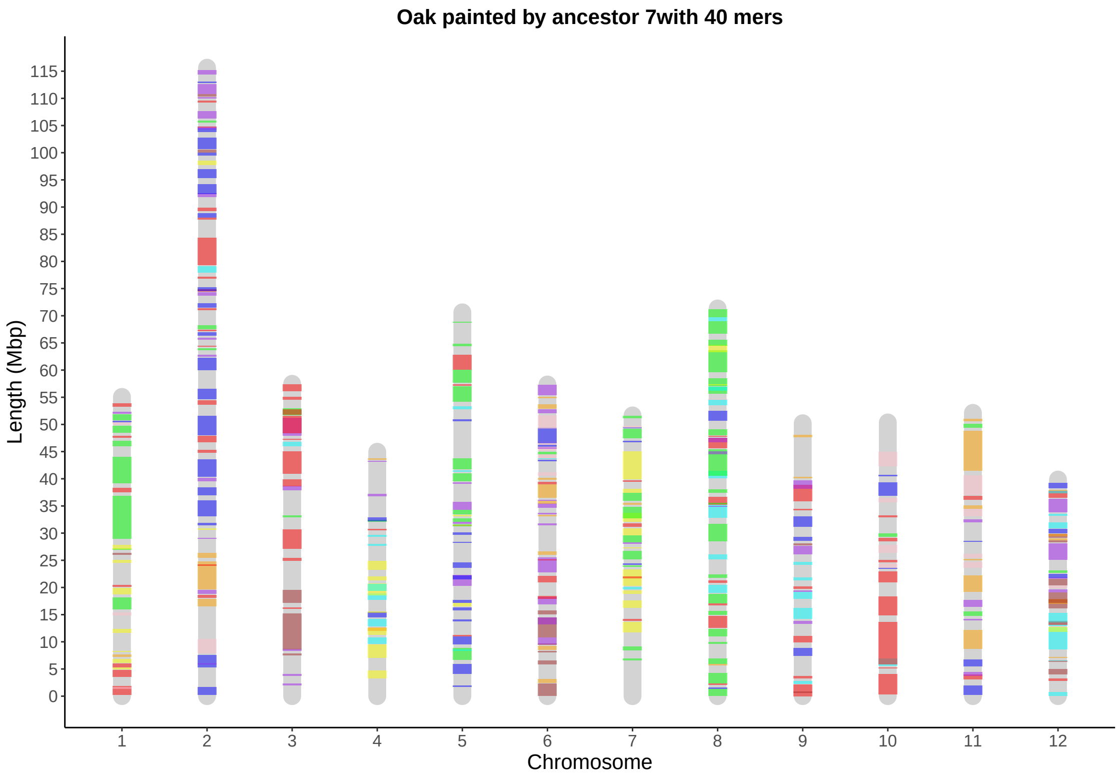

Oak painted by ancestor 7with 40 mers
Length (Mbp)
0
5
10
15
20
25
30
35
40
45
50
55
60
65
70
75
80
85
90
95
100
105
110
115
1
2
3
4
5
6
7
8
9
10
11
12
Chromosome

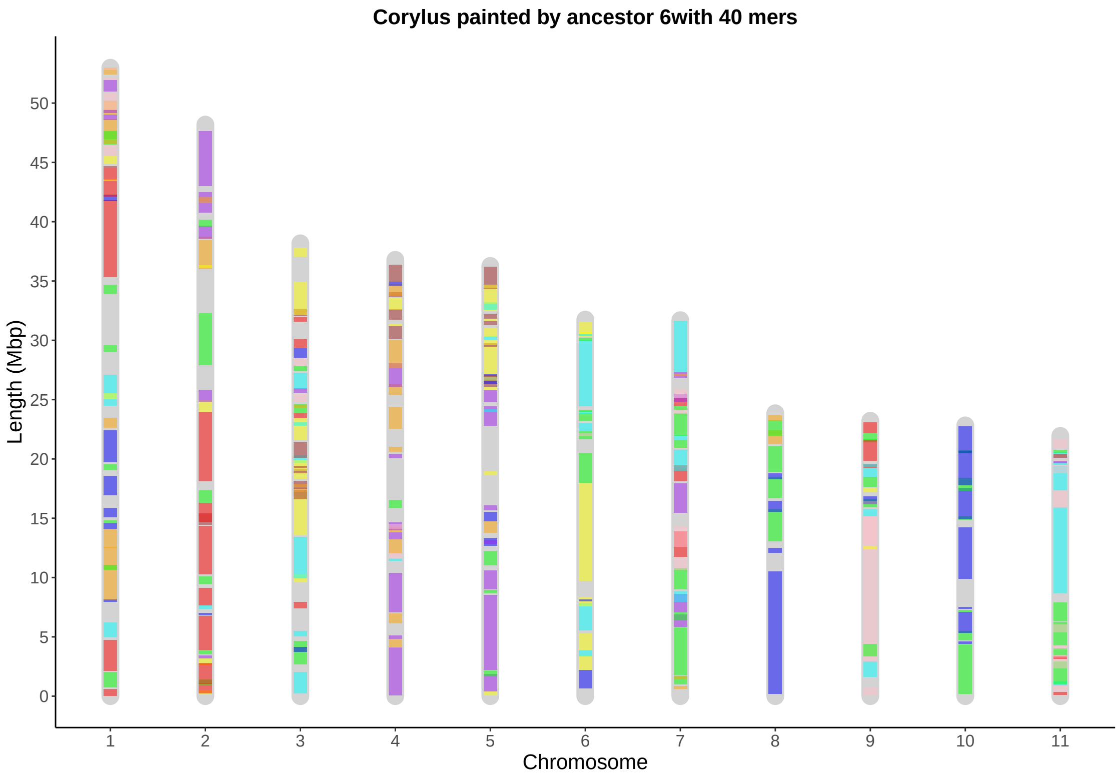

Corylus painted by ancestor 6with 40 mers
Length (Mbp)
0
5
10
15
20
25
30
35
40
45
50
Chromosome
1
2
3
4
5
6
7
8
9
10
11

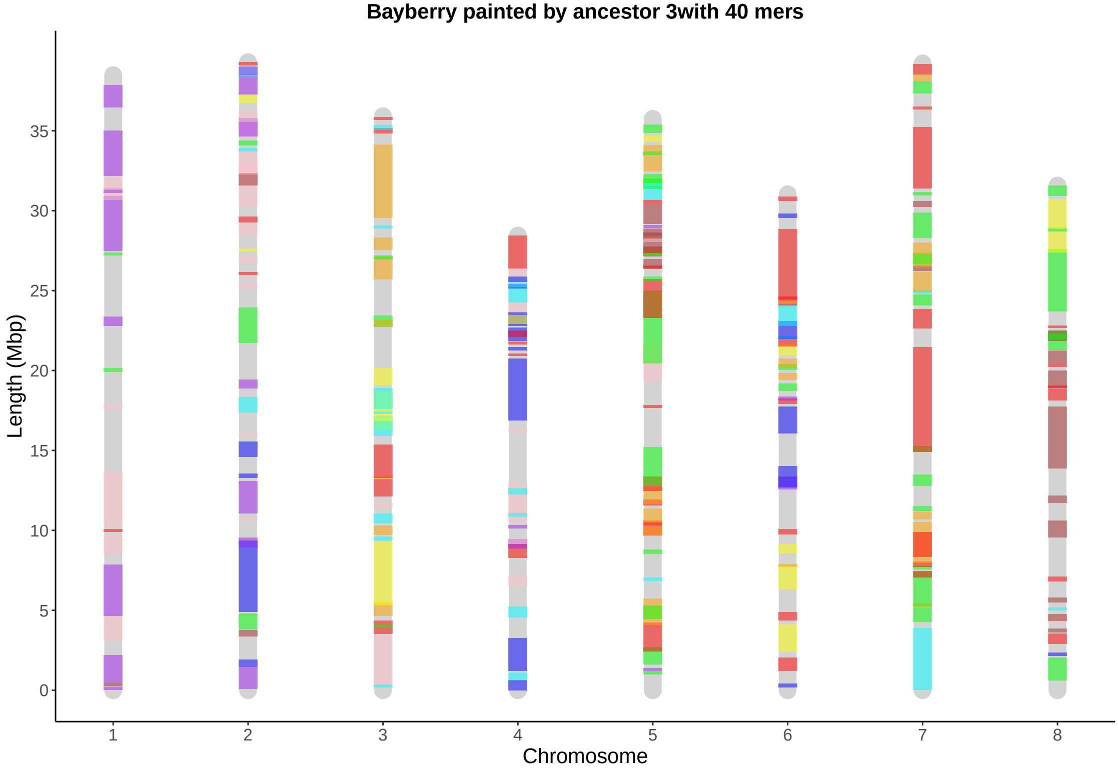

Bayberry painted by ancestor 3with 40 mers
Length (Mbp)
0
5
10
15
20
25
30
35
1
2
3
4
5
6
7
8
Chromosome

Supplement E. PCA clustering $g = 20$

Painting also available for 15-mers, 30-mers and 40-mers.

Fagales Ancestor 1

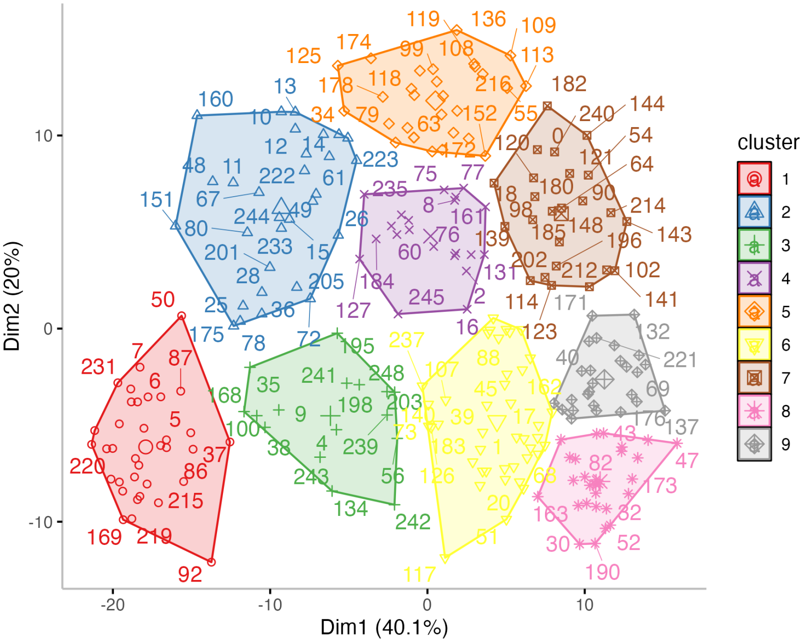

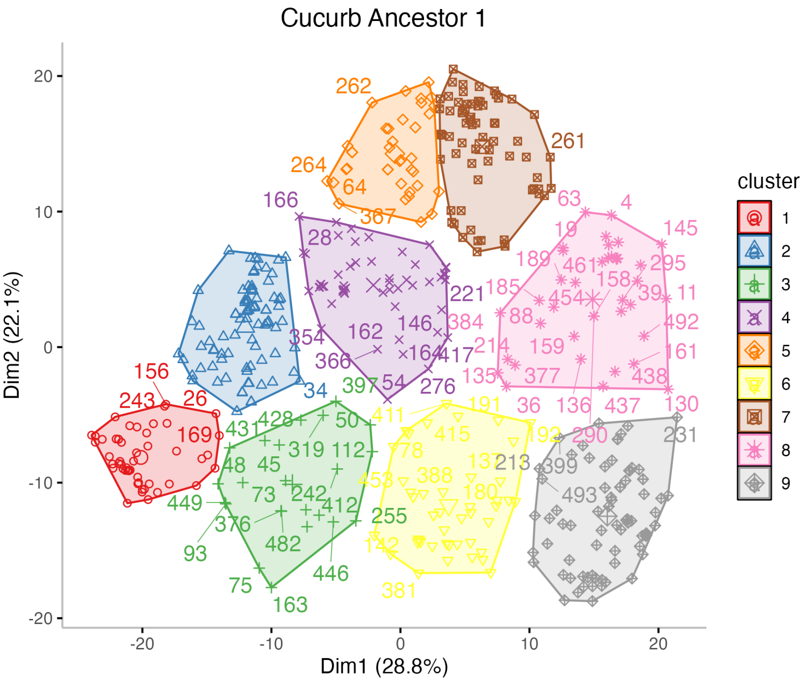

Cucurb Ancestor 1
Dim1 (28.8%)
Dim2 (22.1%)
cluster
1
2
3
4
5
6
7
8
9

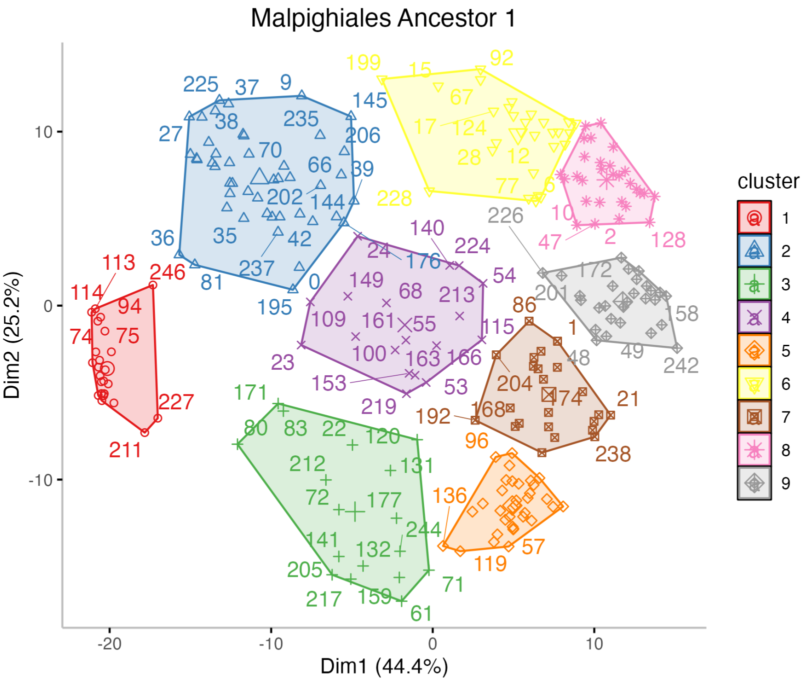
Malpighiales Ancestor 1
Dim1 (44.4%)
Dim2 (25.2%)
-20
-10
0
10
-10
0
10
cluster
1
2
3
4
5
6
7
8
9

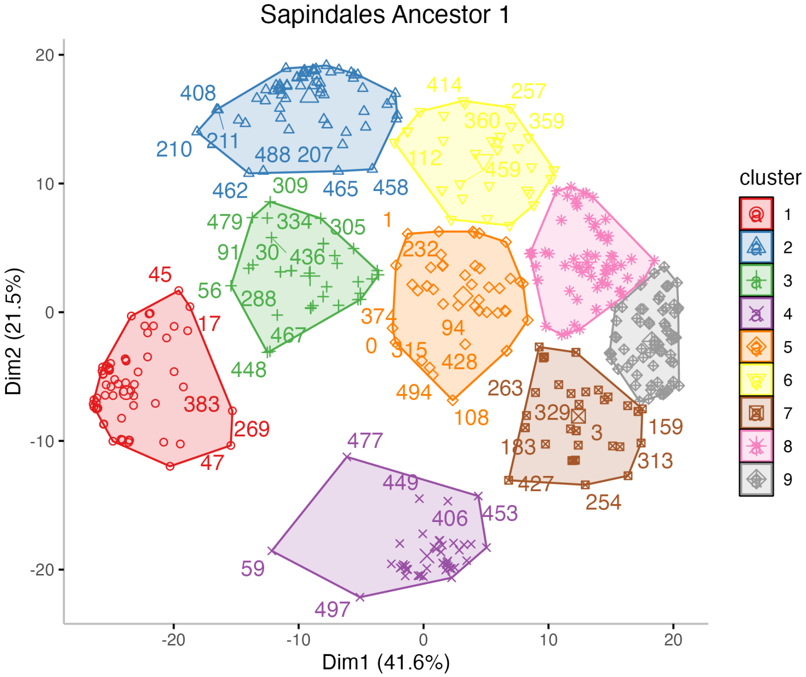

Sapindales Ancestor 1
Dim1 (41.6%)
Dim2 (21.5%)
cluster
1
2
3
4
5
6
7
8
9

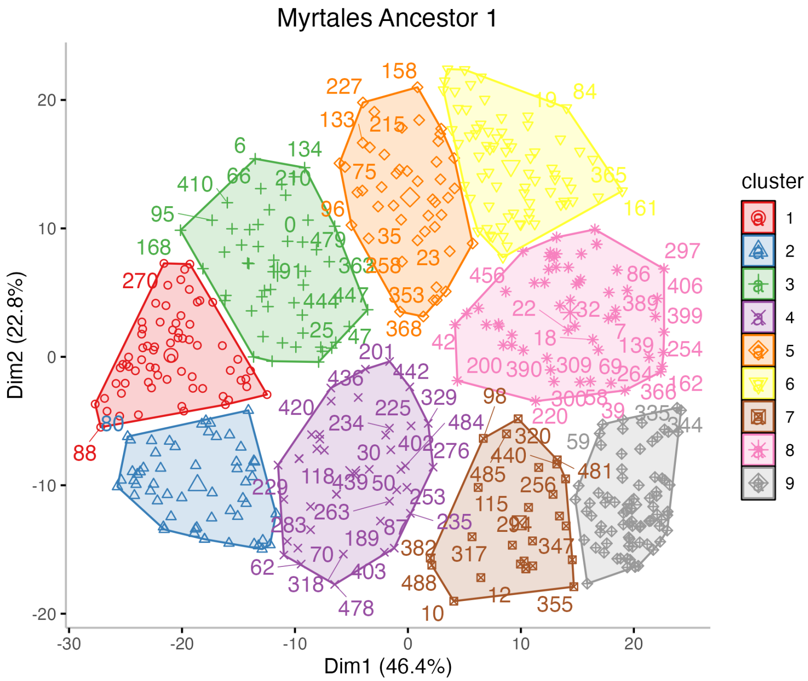

Myrtales Ancestor 1
Dim1 (46.4%)
Dim2 (22.8%)
cluster
1
2
3
4
5
6
7
8
9

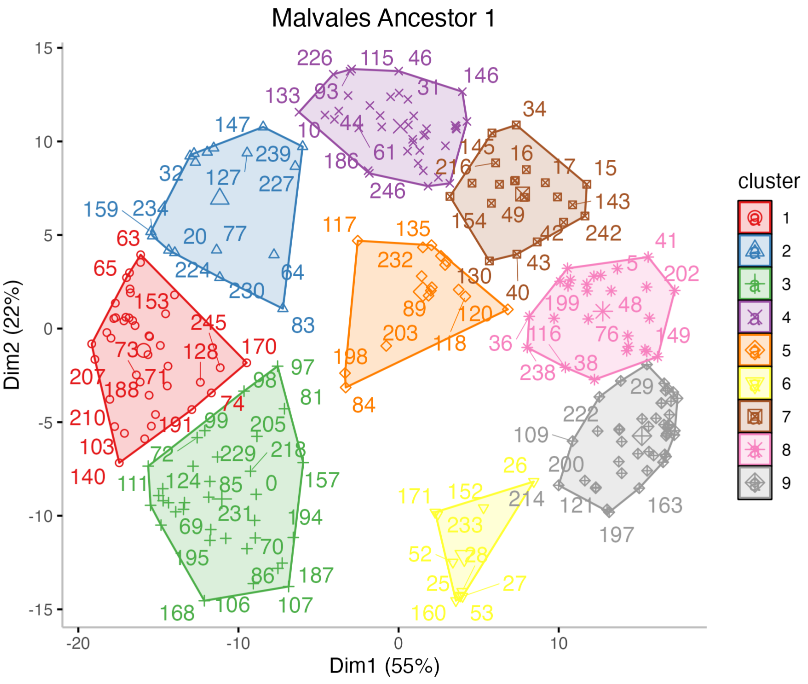
Malvales Ancestor 1
Dim2 (22%)
Dim1 (55%)
15
10
5
0
-5
-10
-15
-20
-10
0
10
cluster
1
2
3
4
5
6
7
8
9

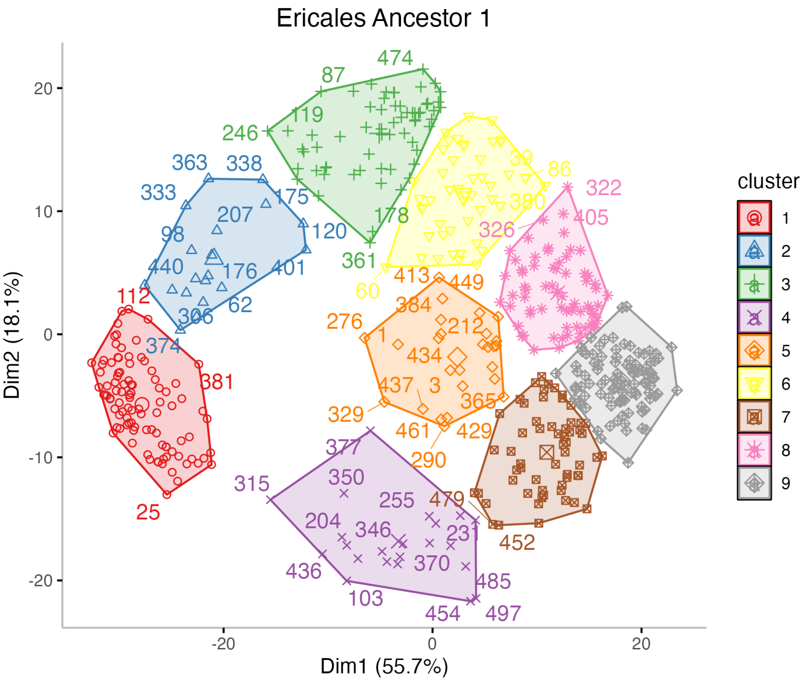
Ericales Ancestor 1
Dim1 (55.7%)
Dim2 (18.1%)
cluster
1
2
3
4
5
6
7
8
9
-20
0
20
-20
-10
0
10
20

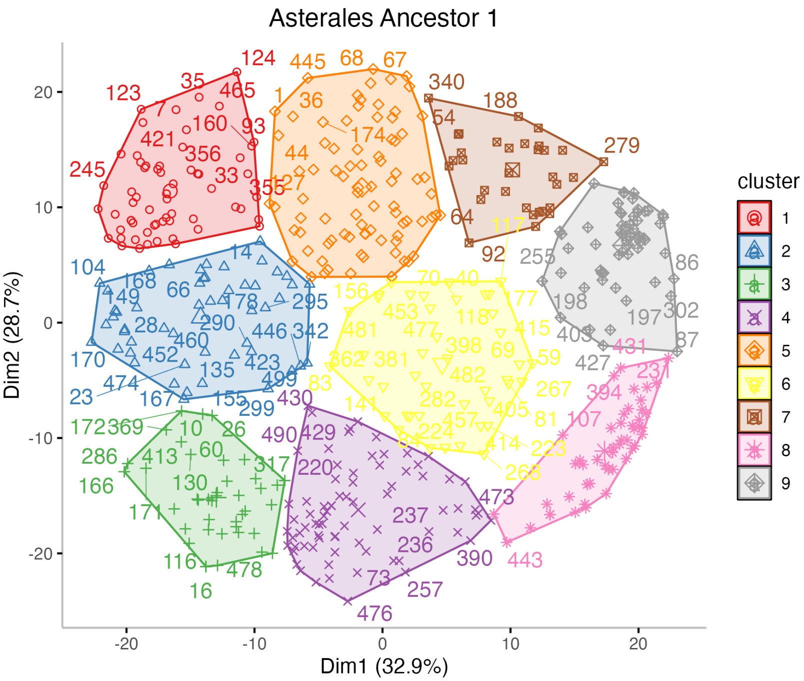
Asterales Ancestor 1
Dim1 (32.9%)
Dim2 (28.7%)
cluster
1
2
3
4
5
6
7
8
9

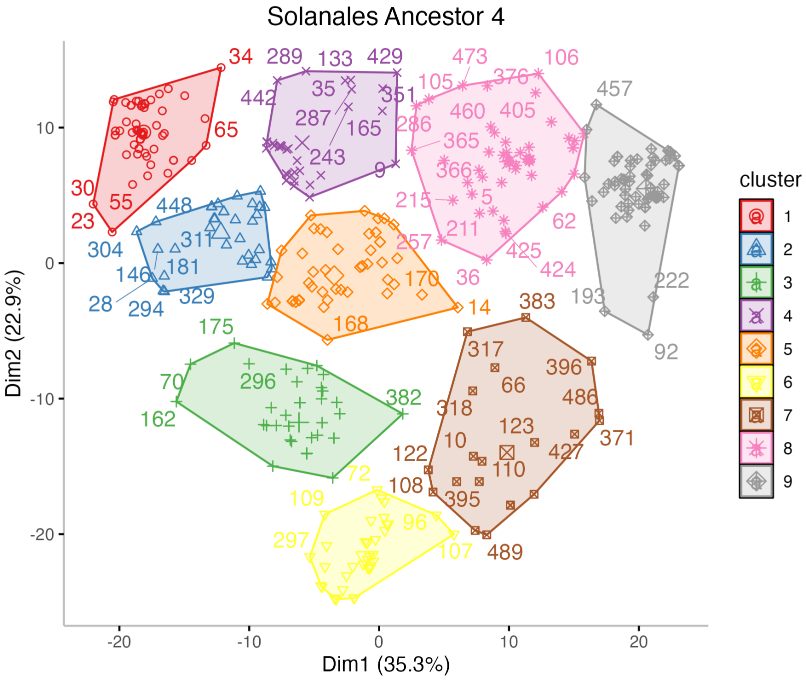
Solanales Ancestor 4
Dim1 (35.3%)
Dim2 (22.9%)
-20
-10
0
10
20
cluster
1
2
3
4
5
6
7
8
9

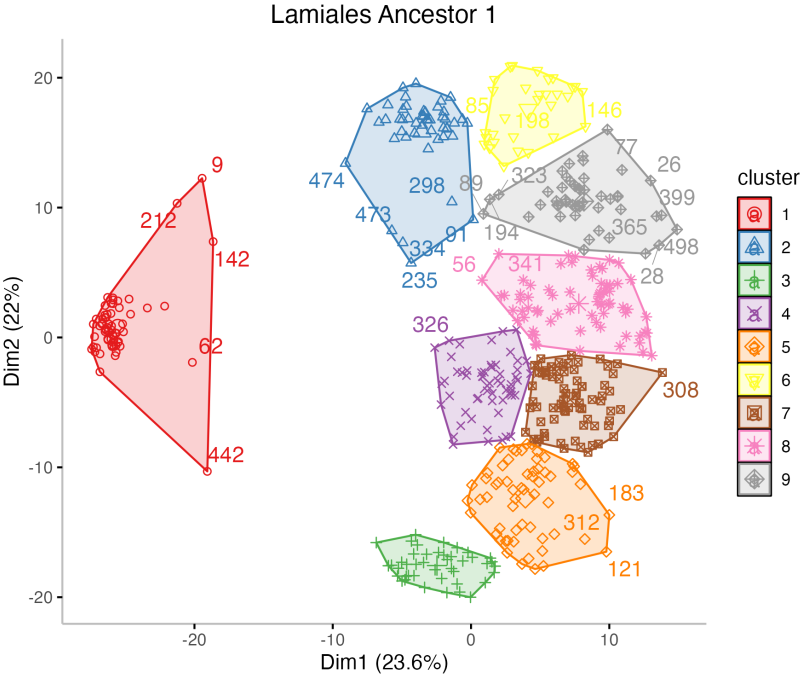
Lamiales Ancestor 1
Dim1 (23.6%)
Dim2 (22%)
cluster
1
2
3
4
5
6
7
8
9

Gentianales Ancestor 1

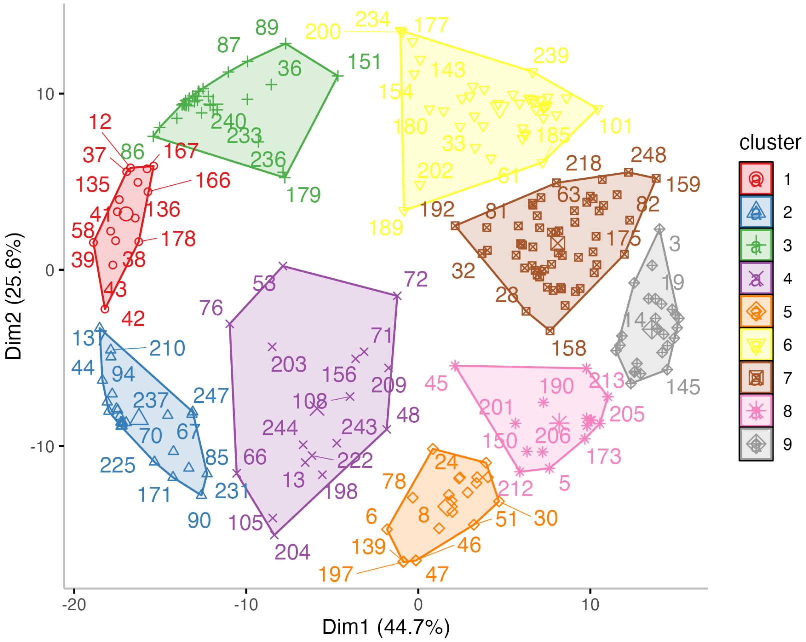

Supplement: Supplementary file 1 — Supplementary Information. [file 41598_2023_33029_MOESM1_ESM.pdf]
